# Extracellular vesicle‐mediated endothelial apoptosis and EV‐associated proteins correlate with COVID‐19 disease severity

**DOI:** 10.1002/jev2.12117

**Published:** 2021-07-03

**Authors:** Balaji Krishnamachary, Christine Cook, Ashok Kumar, Leslie Spikes, Prabhakar Chalise, Navneet K. Dhillon

**Affiliations:** ^1^ Division of Pulmonary and Critical Care Medicine Department of Internal Medicine University of Kansas Medical Center Kansas City Kansas USA; ^2^ Department of Biostatistics & Data Science University of Kansas Medical Center Kansas City Kansas USA

**Keywords:** endothelial injury, inflammation, SARS‐CoV‐2, thrombosis

## Abstract

Coronavirus disease‐2019 (COVID‐19), caused by the novel severe acute respiratory syndrome coronavirus‐2 (SARS‐CoV‐2), has lead to a global pandemic with a rising toll in infections and deaths. Better understanding of its pathogenesis will greatly improve the outcomes and treatment of affected patients. Here we compared the inflammatory and cardiovascular disease‐related protein cargo of circulating large and small extracellular vesicles (EVs) from 84 hospitalized patients infected with SARS‐CoV‐2 with different stages of disease severity. Our findings reveal significant enrichment of proinflammatory, procoagulation, immunoregulatory and tissue‐remodelling protein signatures in EVs, which remarkably distinguished symptomatic COVID‐19 patients from uninfected controls with matched comorbidities and delineated those with moderate disease from those who were critically ill. Specifically, EN‐RAGE, followed by TF and IL‐18R1, showed the strongest correlation with disease severity and length of hospitalization. Importantly, EVs from COVID‐19 patients induced apoptosis of pulmonary microvascular endothelial cells in the order of disease severity. In conclusion, our findings support a role for EVs in the pathogenesis of COVID‐19 disease and underpin the development of EV‐based approaches to predicting disease severity, determining need for patient hospitalization and identifying new therapeutic targets.

## 1 INTRODUCTION

The coronavirus disease‐2019 (COVID‐19) caused by the severe acute respiratory syndrome coronavirus‐2 (SARS‐CoV‐2) (Dashboard WCDC‐ 2020; Wu et al., 2020) is marked by endothelial dysfunction and dysregulated immune responses (Huertas et al., 2020). Like the SARS‐CoV pathogen that led to epidemics in 2002 and 2003, SARS‐CoV‐2 enters cells via binding of its spike protein to angiotensin converting enzyme 2 (ACE2) receptors. ACE2 receptors are abundantly present in pulmonary alveolar type II and endothelial cells, thereby making the lungs and pulmonary vasculature susceptible to SARS‐CoV‐2‐induced inflammation and injury (Zhao et al., 2020). Further, alveolar capillary micro‐thrombi and endothelial damage with evidence of intracellular virus have been noted on post‐mortem analysis of infected lungs (Ackermann et al., 2020).

While there is growing evidence that endothelial injury, vascular remodelling and coagulopathy are key consequences of COVID‐19 infection, it remains unclear how the virus induces these changes. Extracellular vesicles (EVs) carry proteins, coding and non‐coding RNA, DNA fragments and lipids, which facilitate cross‐talk between cells. The transfer of EV cargo plays a significant role in a number of diseases processes, including cardiovascular disease, pulmonary hypertension and various malignancies (Jansen et al., 2017; Meldolesi, 2019). EVs can be released in response to thrombin, shear stress, complement activation and inflammation, among other pathophysiologic triggers. Not only can EVs mediate disease through cellular cross‐talk, but their cargo can also serve as biomarkers of disease status. Sequential analysis of EV contents can provide snapshots of disease progression over time (Urabe et al., 2020). In this study, we analysed alterations in plasma‐derived EVs from patients infected with SARS‐CoV‐2 to investigate the role(s) EVs may play in COVID‐19 disease pathophysiology and to identify potential EV biomarkers of COVID‐19 disease severity and progression.

## 2 MATERIALS AND METHODS

### 2.1 Human samples and data collection

EDTA blood samples were collected from 84 hospitalized subjects 18 years of age or older with a confirmed diagnosis of SARS‐CoV‐2 infection. All subjects were enrolled in the University of Kansas Health System's (TUKHS) COVID‐19 Biorepository and each subject had fasting blood samples collected between 3 and 7 AM at the time of enrolment according to institutional Infection Prevention and Control guidelines. Plasma was isolated within 4 h of blood collection by centrifugation at 2000 g for 15 min at 4°C and then immediately aliquoted and frozen at ‐80°C until further use. The turbidity of plasma was monitored and in case of haemolysis the information was noted and those samples were not used for EV isolation. Demographic information and clinical data were collected and stored in a secure database. Vital signs, oxygen and life support needs and laboratory data were used to calculate acute physiology and early warning scores for patients on lab draw days. Written informed consent for participation in the Biorepository was obtained from all patients or their surrogates. This study and the COVID‐19 Biorepository were approved by the University of Kansas Medical Center Institutional Review Board.

A confirmed case of COVID‐19 infection was defined as a patient with a positive SARS‐CoV‐2 nucleic acid by RT‐PCR. All but one patient tested positive on a nasopharyngeal swab. One patient was presumed positive based on symptoms and reactive serology. Patients were classified by World Health Organization (WHO) Clinical Progression Scale score (Marshall et al., 2020). Specifically, they were grouped by peak score during their hospitalization. Patients were considered asymptomatic if they had a positive SARS‐CoV‐2 RT‐PCR but no COVID‐19 related respiratory symptoms and were hospitalized for another medical reason (Asymptomatic group). These patients were tested for COVID‐19 as part of a hospital‐wide admission or pre‐operative mandate. The remaining patients were categorized as having moderate disease with no oxygen requirement (Moderate‐No O_2_ group), moderate disease requiring supplemental oxygen by simple face mask or nasal prongs (Moderate‐On O_2_ group), or severe disease requiring oxygen delivery by non‐rebreather mask, non‐invasive ventilation, or heated high flow nasal cannula at a minimum (Severe group). Several clinical laboratory parameters were checked every 1 to 3 days as part of centre‐specific standard care for hospitalized COVID‐19‐infected patients. These included white blood cell (WBC) count, lymphocyte count (as percentage of WBC count), creatinine, lactate dehydrogenase (LDH), ferritin, D‐dimer and C‐reactive protein (CRP). Plasma from SARS‐CoV‐2‐positive patients was compared with plasma from uninfected controls (UI‐Control group, n = 15) with matched epidemiological and clinical characteristics (individuals with diabetes, hypertension, etc.). These uninfected EDTA plasma samples obtained from the TUKHS Biospecimen Repository Core Facility (BRCF) were collected before the pandemic and stored in cryovials in a ‐80°C freezer. The non‐fasting EDTA‐blood was centrifuged at 1300 × *g* for 10 min at 4°C within 4 h of collection for plasma isolation. Similarly, a separate set of uninfected EDTA plasma samples from healthy volunteers without any comorbidities, was collected before the COVID‐19 pandemic (Healthy group, n = 6).

#### 2.1.1 Isolation of EVs from plasma samples

The differential ultracentrifugation method with intermediate recovery and intermediate specificity according to MISEV 2018 (Thery et al., 2018) was used to isolate EVs. About 1 ml frozen aliquot of EDTA plasma (never undergone freeze‐thaw cycle before) was thawed at room temperature and centrifuged at 2500 g for 15 min at room temperature to obtain the platelet free plasma (PFP). The PFP was then centrifuged at 20,000 g (Eppendorf, USA) for 15 min at 4°C to isolate the 20K‐pellet large vesicles (LEVs). The 20K pellet was further washed with PBS and centrifuged again at 20,000 g for 15 min at 4°C followed by re‐suspension in 200 μl of PBS (LEVs). After the first 20,000 g spin, the plasma supernatant was filtered through a 0.22 μm filter and subjected to ultracentrifugation at 100,000 g for 70 min at 4°C using a swinging bucket rotor (Thermo Scientific SureSpin 630 (17 ml) Rotor, K Factor 268.0). The pellet was washed with PBS and centrifuged again at 100,000 g using the same rotor for 70 min at 4°C and finally re‐suspended in 500 μl of PBS to obtain small EV (SEV) preparation. In addition, EVs were isolated using another intermediate recovery and intermediate specificity method. The size‐exclusion chromatography (SEC) was performed in small set of samples using qEV original 35 nm columns (Izon Science, Cambridge, MA). The supernatant obtained after removal of 20K‐pellet particles from 0.5 ml of PFP was loaded on the columns rinsed with 0.5 ml of 0.22 μm filtered PBS followed by collection of fractions according to the manufacturer instructions. The fractions were analysed by NanoSight and based on the nanoparticle tracking analysis data four fractions from 7 to 10, rich in EVs were pooled and concentrated using Amicon Ultra‐4 centrifugal filters (10 kDa, Millipore Sigma, USA) to a final volume of 500 μl.

#### 2.1.2 EV characterization

Three different complementary methods were used for characterization of particles per MISEV 2018. **1)** Nanoparticle tracking analysis: LEVs and SEVs were diluted in PBS (1:50) and gently vortexed before being introduced into the sample chamber using a syringe pump. Three video recordings were made for a period of 60 sec each using Nanosight software (NTA 3.4 Build 3.4.003) with settings as follows: detection threshold 6 and camera level 12, followed by manual focusing, which allows for optimum visualization of the maximum number of EVs. The particle concentrations were corrected for the input volume of EV resuspension and dilution necessary for NTA‐reading. **2)** Transmission electron microscopy: A few microliters of LEVs and SEVs were suspended in PBS, adsorbed on the carbon‐coated copper grid by floatation for 20 min and washed with deionized water six times followed by 1% uranyl acetate staining to enhance the contrast. For the IZON SEC samples, the sample was used directly without any dilution and floatation was performed for 2 min followed by washing and staining. The images were acquired using transmission electron microscopy at 100KV emission (JEM‐1400, Jeol, USA) and different size particles were quantitated using Image J. **3)** Western blot analysis: EVs isolated by both methods were lysed in RIPA lysis buffer system supplemented with 200 mM PMSF, protease inhibitor cocktail and 100 mM sodium orthovanadate. The protein estimation was performed using the Pierce BCA protein assay (ThermoScientific). About 20 μg of lysed LEVs or SEVs and 5 μg of SEC‐SEVs were loaded in 10–12% resolving gel and the transfer was performed using the Immobilon membrane. The primary antibodies against various EV markers, such as transmembrane proteins: CD9, CD81, CD63, Integrin β1; cytosolic proteins: Flotillin‐1, TSG101 Alix and negative controls: APOE (lipoprotein marker) and GM130, were diluted in 5% blotting grade blocker in TBST and added on blots for overnight incubation at 4°C. The secondary antibodies used were HRP conjugated goat anti‐mouse IgG or goat anti‐Rabbit IgG antibodies. The blots were developed using the Piercing ECL WB substrate and Super signal West Femto (Thermo Scientific) upon the classic autoradiography film (MidSci). The details of the reagents and antibody source/dilutions are listed in the Supplementary Table 1.

#### 2.1.3 Uptake of labelled EVs by endothelial cells

The human pulmonary microvascular endothelial cells (HPMECs) (ScienCell, USA) were plated on coverslips in a 24‐well plate (2.5 × 10^4^/well) in complete endothelial cell growth medium (ScienCell, USA). After 24 h, the cells were washed with serum‐free endothelial cell media, followed by treatment with and without 10μM of dynasore (MedChemExpress, USA) for 30 min and the addition of 50 μg (approximately 3.8 × 10^4^/cell) of PKH‐67‐labeled SEVs. PKH67 labelling was performed according to the manufacturer's instructions with minor modifications as described before (Xiao et al., 2014). To remove the unbound dye, the labelled EV preparations were transferred to 300 kDa Vivaspin filters and centrifuged at 4000 g for 2 min at 4°C followed by washing three times with PBS. After 16 h of treatment with the PKH‐67 labelled EVs, the cells were washed with PBS, fixed with 4% Formaldehyde in PBS and blocked with 1% BSA containing 0.2% Triton X in PBS. Next, the Alexa Fluor® 594 phalloidin was added to the cells and incubated for 20 min. The cells were then washed three times with PBS, mounted with ProLong Gold AntiFade Reagent with DAPI and imaged using a Nikon‐80i microscope. Alternatively, SEVs labelled using ExoGlow‐Membrane™ EV Labeling Kit (System Biosciences, USA) were used to check the uptake of EVs by endothelial cell. Briefly, 2 μl of labelling dye was diluted in the 12 μl of reaction buffer. To this, 50 μg of SEVs were added, mixed and incubated for 30 min at room temperature. To remove the free unlabelled dye, 35 μl of ExoQuick‐TC was added to the sample and incubated at 4°C for 30 min, followed by centrifugation at 10,000 rpm for 10 min. The EVs were re‐suspended in PBS and added to the HPMECs pre‐treated with or without dynasore followed by visualization of EV uptake as described above.

#### 2.1.4 Caspase 3/7 assay

For the apoptosis analysis HPMECs (3 × 10^3^ cells/well) were plated on a 96‐well plate (Corning USA). After 24 h, the complete media was removed, and the cells were washed and then treated with 3 μg large or small EVs (, ranging from 2.5‐4.6 × 10^4^/cell) across groups Supplementary Table 2) or equal number of LEVs/SEVs (3.3 × 10^4^ EVs/cell) in serum‐free endothelial cell media. Twenty‐four hours post‐treatment, caspase 3/7 assay was performed using Cell Meter™ Caspase 3/7 activity assay kit according to the manufacturer's instructions (AAT Bioquest, USA). For apoptosis analyses of HPMECs in response to plasma treatment in the presence and absence of EV uptake inhibitors, cells were pre‐treated with heparin (Sigma Aldrich, USA) or dynasore (MedChemExpress, USA) for 30 min followed by treatment with 3 μg protein equivalent of 50 times diluted plasma, followed by caspase 3/7 assay as described above.

#### 2.1.5 Annexin V imaging

Cells were plated on coverslips in a 24‐well plate in complete endothelial cell growth medium (ScienCell, USA). After 24 h, the cells were washed with serum‐free endothelial cell media, followed by treatment with 10 μg of EVs. After 24 h treatment, cells were washed with ice‐cold PBS, followed by the addition of 20 μl of FITC‐Annexin‐V (Thermofisher Scientific, USA) for 15 min. The stained cells were finally mounted ProLong Gold AntiFade Reagent with DAPI and imaged using a Nikon‐80i microscope.

#### 2.1.6 Proximity extension analysis (PEA)

LEVs and SEVs from different groups were lysed using the lysis buffer as previously described (Larssen et al., 2017). Fifty microliters of the lysed sample at an equal concentration (1 μg/μl for LEVs or 0.5 μg/μl for SEVs) was added in a randomized order to a 96‐well PCR plate and sent to Olink Proteomics (Boston, USA) for the analysis. The LEV and SEV cargo was analysed using the Olink Inflammatory and Cardiovascular II and III panels. PEA‐based Olink platform is a multi‐plex DNA‐coupled immunoassay‐based targeted proteomic approach in which each target protein is detected by a pair of unique oligonucleotide‐labelled antibodies. When these oligonucleotide probes come in close proximity on binding to the protein, they polymerize to form a target sequence that is later quantified by RT‐PCR. Samples were randomly plated, and quality control of assay and individual samples was monitored using four internal controls. The results were expressed as normalized protein expression (NPX) expressed on a log2 scale, with a high NPX value indicating higher protein expression. Some of the NPX values in certain groups were observed to be below the limit of detection (LOD) however based on Olink's recommendation all the NPX data values were used to identify statistically significant differences across groups. According to the company, ‘using data below LOD does not generally increase false positives in statistical tests, and since LOD estimates are relatively conservative (3 standard deviations away from the real lower level of quantification) there may still be useful signal below LOD. The data below LOD may have a higher noise to signal ratio, and may not be quite as linear as the portion of the assay measurement curve above LOD, but this shouldn't substantively alter assay orderings when there is a meaningful biological difference. The likelihood of generating spurious statistically significant differences is consequently quite low.’ The samples that failed technical criteria of the assay were excluded.

#### 2.1.7 Tissue factor activity assay

TF activity was measured using Human Tissue Factor (TF) Chromogenic AssaySense Activity Kit (AssayPro, USA) compatible for EDTA plasma samples according to the manufacturer's instructions. Briefly, 30 μl of LEVs ‐ and SEVs were incubated with human Factor VII and X for 30 min at 37°C, followed by the addition of Factor Xa substrate and reading at 405 nm immediately for background readings, which was then followed by incubation at 37°C. Absorbance of chromophore product was measured every 5 min for a total 35 min. TF activity was calculated from the change in absorbance using the standard curve generated by the regression analysis of the four‐parameter curve.

#### 2.1.8 Immunocapturing of EVs

To examine whether some of the cytokine markers are on the surface of SEVs, EVs were captured using specific biotinylated antibodies linked to streptavidin magnetic beads using EXOFLOW2‐BASICA‐SP kit (System Biosciences, USA). The magnetic streptavidin Exo‐Flow 2.0 beads were mixed with 1 μg of the biotin labelled anti‐human CD218a (IL‐18Rα), anti‐human CD126 (IL‐6Rα) or mouse IgG1 antibodies in 250 μl of blocking buffer and incubated at 4°C for 1 h in a rotating rack. After washing the magnetic beads to remove unbound antibodies, 250 μl of blocking buffer was again added to the beads and incubated at room temperature for 30 min followed by washing and addition of 100 μg of SEVs containing blocking buffer and overnight incubation at 4°C in a rotating rack. The experiments were performed in duplicates, so that the captured EVs were subjected to both Western blotting and flow cytometry. After washing, the beads were lysed using RIPA lysis buffer and loaded on the gel for Western blot analysis of EV markers. For flow cytometry, the beads were washed and incubated in blocking buffer II provided by the manufacturer for 1 h at room temperature with rotation. After washing, the beads were resuspended in 240 μl of EV stain buffer and 10 μl of S/P green dye and placed in rotation for 1 h at room temperature followed by washing and analysis of beads resuspended in 300 μl of PBS using BD LSR II flow cytometer.

#### 2.1.9 ELISA

Tissue Factor and EN‐RAGE ELISAs (Abcam, USA) were performed on the EV protein extract according to the manufacturer's instructions.

#### 2.1.10 Statistical analyses

The demographic and clinical characteristics for the disease status groups were summarized with median and interquartile range for the continuous variables and with frequencies and percentages for categorical variables, Table 1. The differences in the continuous variables across the groups were assessed using analysis of variance (ANOVA) followed by post hoc comparisons using Tukey's test. Normality assumption of the data were examined by histogram and boxplots prior to performing the ANOVA analyses. Log transformation was used for the outcomes not satisfying the normality assumption. The categorical outcomes across the groups were assessed using Fisher exact test. Differences in the clinical variables between the genders were assessed using t‐tests or Wilcoxon Rank Sum tests. The differences in the protein expressions among the five groups (Uninfected, Asymptomatic, Moderate‐No O_2_, Moderate‐On O_2_, and Severe) were assessed using ANOVA analyses followed by pairwise post hoc comparison using Tukey's test for each protein. Spearman's correlation analyses were carried out to assess the relationship between each protein with age, BMI, WBC count, LDH, ferritin, CRP, creatinine, D‐dimer, absolute lymphocyte count, length of hospitalization (LH) and disease severity score for all symptomatic patients and then by separate groups. All correlation analyses with length of hospitalization were carried with and without excluding the two extreme observations. Multiple testing adjustments were carried out using Benjamini and Hochberg's false discovery rate (FDR) method. The statistical analyses were carried out using the statistical software R version 4.0.0 (Core Team, 2020) For the nanoparticle tracking analysis, cell‐culture experiments, TF activity assays and ELISAs, statistical analyses were carried out using Graph Pad Prism 9. One‐way ANOVA was performed among the groups followed by Bonferroni's multiple comparison test. The differences in the NPX values or TF activity between survived and deceased groups were assessed using unpaired t‐test (Mann‐Whitney test).

**TABLE 1:** Demographics & characteristics of patients included in analysis

				On Oxygen		
	Uninfected	Asymptomatic	Moderate Disease: Without Oxygen	Moderate Disease: With Oxygen	Severe Disease	*P*‐value
	(n = 15)	(n = 9)	(n = 20)	(n = 20)	(n = 35)	
**Age (median, IQR)**	**51 (34.0 – 61.0)**	**39.0 (37.0‐71.0)**	**49.5 (34.5‐59.5)**	**64.0 (50.5‐81.8)**	**63.0 (53.8‐71.0)**	0.0027
**Sex (total, %)**						0.9722
Male	7 (46.7%)	5 (55.6%)	9 (45.0%)	11 (55%)	18 (51.4%)	
Female	8 (53.3%)	4 (44.4%)	11 (55.0%)	9 (45.0%)	17 (48.6%)	
**Race (total, %)**						0.2039
Asian	0 (0%)	0 (0.0%)	1 (5.0%)	0 (0.0%)	0 (0.0%)	
Black or African American	5 (33.3%)	3 (33.3%)	5 (25.0%)	6 (27.3%)	5 (14.3%)	
White	9 (60.0%)	6 (66.7%)	8 (40.0%)	8 (40.0%)	18 (51.4%)	
Other	1 (6.7%)	0 (0.0%)	6 (30.0%)	6 (30.0%)	12 (34.3%)	
**Ethnicity (total, %)**						0.0392
Hispanic	1 (6.7%)	0 (0.0%)	6 (30.0%)	6 (30.0%)	14 (40.0%)	
Non‐Hispanic	14 (93.3%)	9 (100.0%)	14 (70.0%)	14 (70.0%)	21 (60.0%)	
**BMI (median, IQR)**	**25.32 (23.3‐29.6)**	**23.4 (22.2‐ 37.6)**	**29.7 (24.7‐32.9)**	**33.0 (26.6‐38.5)**	**33.8 (27.4‐41.8)**	0.0022
**BMI classification (total, %)**						
Below 18.5 (underweight)	0 (0.0%)	0 (0.0%)	1 (5.0%)	0 (0.0%)	0 (0.0%)	
18.5 to 24.9 (normal)	6 (40.0%)	6 (66.7%)	4 (20.0%)	3 (15.0%)	4 (11.4%)	
25.0 to 29.9 (overweight)	6 (40.0%)	0 (0.0%)	5 (25.0%)	4 (20.0%)	8 (22.9%)	
30.0 to 34.9 (class I obesity)	1 (6.7%)	0 (0.0%)	7 (35.0%)	4 (20.0%)	6 (17.1%)	
35.0 to 39.9 (class II obesity)	2 (13.3%)	1 (11.1%)	3 (15.0%)	4 (20.0%)	5 (14.3%)	
40.0 + ‐ (class III obesity)	0 (0.0%)	2 (22.2%)	0 (0.0%)	5 (25.0%)	12 (34.3%)	
**Comorbidities (total, %)**						
HTN	4 (26.7%)	2 (22.2%)	8 (40.0%)	12 (60.0%)	18 (51.4%)	0.1737
Diabetes (type 1 or 2)	3 (20.0%)	1 (11.1%)	8 (40.0%)	5 (25.0%)	19 (54.3%)	0.0398
Coronary artery disease	1 (6.7%)	0 (0.0%)	1 (5.0%)	4 (20.0%)	4 (11.4%)	0.5425
Chronic renal insufficiency	1 (6.7%)	2 (22.2%)	4 (20.0%)	6 (30.0%)	4 (11.4%)	0.3235
Hyperlipidaemia	5 (33.3%)	2 (22.2%)	5 (25.0%)	7 (35.0%)	14 (40.0%)	0.7886
Heart failure (systolic or diastolic)	1 (6.7%)	1 (11.1%)	1 (5.0%)	2 (10.0%)	5 (14.3%)	0.8747
COPD	1 (6.7%)	2 (22.2%)	0 (0.0%)	1 (5.0%)	1 (2.9%)	0.1381
**WHO Peak Score (Median, IQR)**	**N/A**	**4 for all**	**4 for all**	**5 for all**	**7.0 (6.0‐10)**	
**Mortality (total, %)**	**N/A**	**0 (0.0%)**	**0 (0.0%)**	**0 (0.0%)**	**11.0 (31.4%)**	
**Treatment**						
Antimicrobials		0 (0.0%)	5 (25.0%)	3 (15.0%)	8 (22.9%)	
Steroids		3 (33.3%)	4 (20.0%)	8 (40.0%)	34 (97.1%)	
Non‐steroid immune suppressants		0 (0.0%)	0 (0.0%)	1 (5.0%)	11 (31.4%)	
Anti‐viral therapy		0 (0.0%)	0 (0.0%)	12 (60.0%)	32 (91.4%)	
Convalescent plasma		1 (11.1%)	0 (0.0%)	10 (50.0%)	24 (68.6%)	
Anticoagulant (therapeutic only)**		2 (22.2%)	4 (20.0%)	2 (10.0%)	18 (51.4%)	
**Time from symptom onset to hospital admission (days) (median, IQR)**	**N/A**	**N/A**	**7.0 (3.0‐11.0)**	**6.5 (4.0‐10.0)**	**6.0 (4.0‐9.0)**	0.9319
**Time from symptom onset to first draw (days) (median, IQR)**	**N/A**	**N/A**	**9.0 (5.8‐14.0)**	**9.5 (6.0‐12.3)**	**11.0 (8.0‐15.0)**	0.1882
**Length of hospitalization (days) (median, IQR)**	**N/A**	**N/A**	**3.0 (2.8 – 4.3)**	**8.0 (5.8 ‐10.5)**	**17.0 (13.0‐23.0)**	1.1e‐8
**Lab data (within 24** **h of first draw) (median, IQR)**						
White blood cell count, count x 1000/μl		7.7 (5.8‐8.4)	5.8 (4.1‐8.2)	5.7 (4.5‐10.4)	10.0 (7.3‐14.2)	0.0011
Lymphocytes, percentage		27.5 (22.8‐33.3)	23.5 (13.0‐29.0)	20.0 (8.5‐26.5)	9.0 (5.0‐11.0)	1.6e‐5
Creatinine, mg/dl		0.7 (0.7‐0.9)	0.9 (0.6‐1.2)	0.89 (0.7‐1.2)	0.9 (0.8‐1.3)	0.5724
Lactate dehydrogenase, units/L			214.5 (170.5‐287.0)	394.5 (335.0‐470.2)	416.0 (347.0‐551.0)	0.0005
Ferritin, ng/ml			365.0 (246.5‐736.5)	416.0 (300.0‐562.2)	515.0 (282.0‐879.0)	0.5016
D‐dimer, ng/ml			674.0 (418.5‐951.5)	592.0 (425.0‐676.0)	1388.0 (820.0‐3885)	1.8e‐4
C‐reactive protein, mg/dl			3.1 (2.1‐6.9)	8.93 (5.4‐15.2)	9.5 (2.8‐18.3)	0.0307
**Number of samples used for various assays**						
*Nanoparticle Tracking Analysis*	15+6*	9	20	20	21	
*LEVs Olink Analysis (n)*	6*	6	13	14	9	
*SEVs Olink Analysis (n)*	15+ 6*	9	15	15	15	
*Apoptosis assay using EVs (n)*	15+5*	9	15	15	15	
*Apoptosis assay using Plasma (n)*	8				8	
*Tissue factor activity assay (n)*	15		15	19	35	
*EN‐RAGE ELISA, UC‐SEVs*	10		10	10	10	
*SEC‐EVs: Nanoparticle Tracking Analysis, EN‐RAGE ELISA, TF ELISA, TF activity*	5*		5	5	5	

*Additional 6 un‐infected samples from healthy volunteers were used.

**Patients received at least 24 hours of therapeutic anticoagulation during hospitalization. All patients not receiving therapeutic anticoagulation received an escalated dose of Enoxaparin for venous thromboembolism prophylaxis.

#### 2.1.11 Data availability

We have submitted relevant details related to EV methodology to the EV‐TRACK knowledgebase (EV‐TRACK ID: EV210166) (Van Deun et al., 2017).The NPX values of the PEA Olink analyses are provided as a Supplementary material.

## 3 RESULTS

### 3.1 Demographic and clinical characteristics

Of the 84 hospitalized patients with blood samples included in our analysis, 9 had no respiratory symptoms (Asymptomatic), 20 had moderate disease but were not hypoxic (Moderate‐No O_2_), 20 had moderate disease and were hypoxic (Moderate‐On O_2_) and 35 had severe COVID‐19 pneumonia (Severe) (Table 1). Although the median age of patients on oxygen support was significantly higher compared to other groups (*P* = 0.0027), no difference was observed between patients in the Moderate‐On O_2_ and Severe groups. Median time from symptom onset to hospital admission was 6 days. Median time from symptom onset to baseline lab draw was 10 days with no statistically significant differences across the groups. The median length of hospitalization (LH) for the Moderate‐No O_2_ group was 3 days compared to 17 days for the Severe group. The length of hospitalization for two patients from the Severe group were 77 and 99 days; however, for the remaining patients, the range was 2 to 35 days. Fifty‐one percent of patients were males, and 27% of patients included in the study were identified as Hispanic. More than 50% of patients met criteria for class I, II, or III obesity, and the difference in median BMI between groups was statistically significant (*P* = 0.0022) with patients requiring oxygen support having a median BMI of 33. The most common comorbidities among these patients were hypertension (42.4%), diabetes mellitus (type 1 or 2) (35.4%), dyslipidaemia (32.3%), and chronic renal insufficiency (17.2%). There was a statistically significant difference (*P* = 0.0398) in the median number of diabetic patients across the groups with more than 50% of patients in the Severe group having diabetes and only one patient in the Asymptomatic group being diabetic. Interestingly, patients from the Moderate‐No O_2_ and Severe groups had a higher percentage of diabetes (40% and 54.3%, respectively) compared to patients in the Moderate‐On O_2_ group (25%). There were no significant gender differences in comorbid conditions among the groups. Thirty‐eight percent of patients infected with SARS‐CoV‐2 were current or former smokers. Fifty‐eight percent of infected patients had no history of tobacco, alcohol, or drug abuse. Of the 35 patients with severe disease, 16 were mechanically ventilated and 11 died during hospitalization.

Among asymptomatic patients included in our analysis, reasons for hospitalization included post‐operative C*lostridium difficile* infection, hyperemesis gravidarum, sickle cell pain crisis, acute variceal haemorrhage, heart failure exacerbation with newly reduced left ventricular ejection fraction, post‐operative pelvic abscess, gunshot wound, osteomyelitis and pathologic thoracic compression fracture.

In terms of treatment, the accepted management of COVID‐19‐infected patients has evolved over the course of the pandemic. For example, after publication of the results of the RECOVERY trial in the *New England Journal of Medicine* in July 2020 (Group et al., 2020), hypoxemic COVID‐19‐positive patients at TUKHS began to receive corticosteroids as standard of care. Patients also have been enrolled in a variety of clinical trials designed to address the dysregulated immune and prothrombotic responses seen in COVID‐19 infection. Table 1 lists the treatments that patients received for at least 24 h of their hospitalization, including antimicrobial therapy (including the anti‐malarial agent, hydroxychloroquine); corticosteroids; nonsteroidal immune suppressants; antiviral therapy; convalescent plasma; and/or therapeutic anticoagulation. Therapies administered as part of a clinical trial were not included in this analysis. Importantly, all patients not receiving therapeutic anticoagulation received an escalated dose of enoxaparin for venous thromboembolism (VTE) prophylaxis unless they had an absolute contraindication. Among patients in the Severe group, 22.9% received antimicrobial therapy, 97.1% received corticosteroids, 31.4% received a non‐steroidal immune suppressant and 91.4% received antiviral therapy (remdesivir). Nearly 70% were treated with convalescent plasma, and approximately 50% received therapeutic anticoagulation, compared to only 10 to 20% of patients in other groups. Seventeen percent of patients in the Severe group were on therapeutic anticoagulation prior to admission.

Regarding laboratory data collected from symptomatic COVID‐19 patients within 24 h of baseline lab draw, median CRP (*P* = 0.0307) and LDH (*P* = 0.0005) levels increased with progression of disease severity (Table 1). Median D‐dimer levels were significantly higher in patients in the Severe group compared to patients with moderate disease (*P* = 1.8 × 10^−4^). There also was a significant difference in median WBC count in patients from the Severe group compared to those in the Moderate groups (*P* = 0.0011) though WBC count values remained within the normal range. Patients in the Severe group had significant lymphopenia compared to patients in other groups (*P* = 1.6 × 10^−5^). However, no significant differences were observed in baseline creatinine or ferritin levels among the groups.

As illustrated in Supplementary Figure 1, length of hospitalization (LoH) positively correlated with age LDH, D‐dimer and WBC count, but negatively correlated with lymphocyte count. These correlations and statistical significances remained similar with (data not shown) and without including extreme LoH observations ; patients with 77 and 99 days of hospitalization. However, the statistical significance of the positive correlation of LoH with CRP was altered after excluding the extreme observations (*P* = 0.03 including and *P* = 0.06 excluding the two outliers). No significant correlation was observed between length of hospitalization and BMI, creatinine or ferritin levels. As one would expect, length of hospitalization correlated with increasing disease severity as defined by peak WHO Clinical Progression Scale score (R = 0.6839, *P* = 2.59 × 10^−11^). There were no significant gender differences in age, BMI, or laboratory parameters, with the exception of ferritin levels, which were significantly higher in males (*P* = 0.0025).

### 3.2 Characterization of plasma‐derived large and small EVs

**FIGURE 1:**
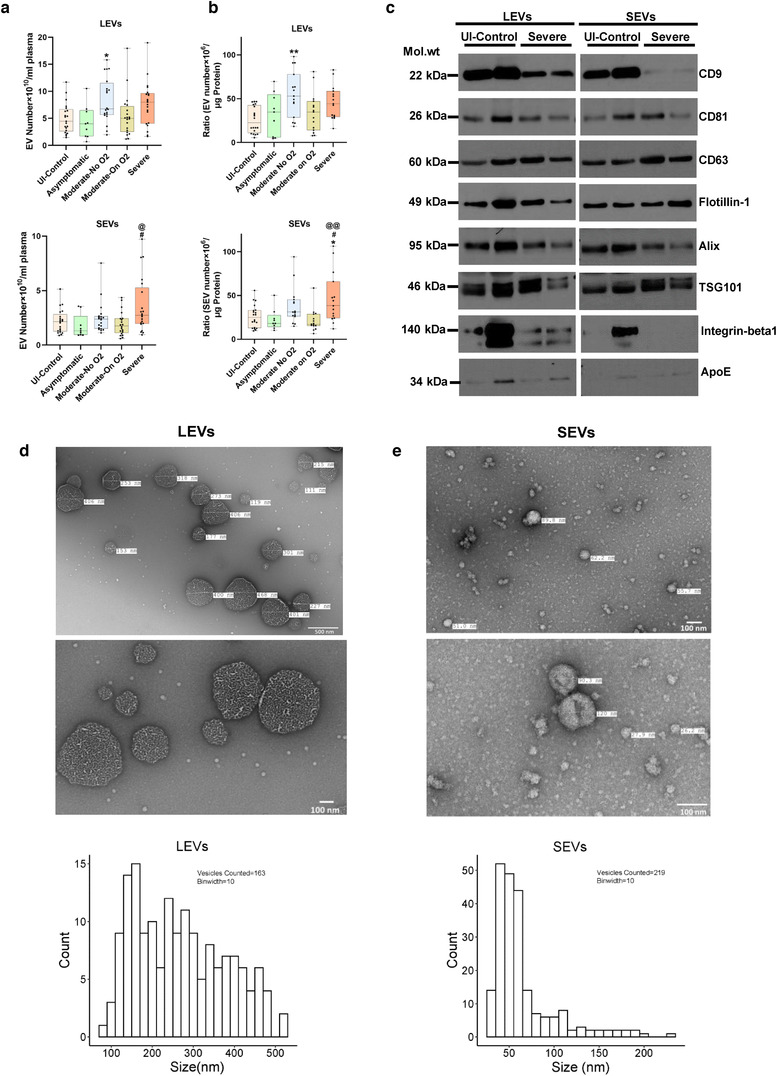
Characterization of extracellular vesicles isolated from plasma of COVID‐19 infected and uninfected individuals. Extracellular vesicles (EVs) were isolated from 1.0 ml of platelet free EDTA‐plasma from Uninfected controls (n = 21), Asymptomatic (n = 9), Moderate‐No O2 (n = 20), Moderate‐On O2 (n = 20) and Severe (n = 21) group COVID‐19 patients by centrifuging at 20,000 g for 15 min (20K pellet large EVs) followed by ultracentrifugation twice at 100,000 g for 70 min (100K pellet small EVs). a) Total large EVs (LEVs, top panel) and small EVs (SEVs, bottom panel) in each group based on nanoparticle tracking analysis. Data represents number of EVs/ml of final EV suspension obtained from 1 ml of EDTA plasma. b) EV to protein ratio in each group Uninfected Control (n = 20), Asymptomatic (n = 9), Moderate‐No O2 (n = 15), Moderate‐On O2 (n = 15), Severe (n = 15). c) Western blot analysis of EV markers in LEVs and SEVs from un‐infected controls (n = 2) and Severe COVID‐19 (n = 2) patients. d‐e) Representative images and particle size distribution of LEVs (d) and SEVs (e) based on Transmission electron microscopy (TEM). Approximately 10–15 TEM images of LEVs and SEVs each were counted using the Image J software. Box plots depicts median and IQR and whiskers represent minimum to maximum points. * *P* < 0.05, ** *P* < 0.01 vs UI‐Control, # *P* < 0.05 vs. Asymptomatic, @ *P* < 0.05, @@ *P* < 0.01 vs Moderate‐On O2

Both large (20K‐pellet) and small (100 K‐pellet) EVs isolated from the plasma of all SARS‐CoV‐2‐positive patients and healthy uninfected controls were compared for total number and size using nanoparticle tracking analysis. An increase in total number of LEVs was observed in the Moderate‐No O_2_ and Severe groups compared to the other groups (Figure 1a, top panel). However, an increase in SEVs was only observed in the Severe group when compared to other COVID‐19‐positive and ‐negative groups (Figure  1a, bottom panel). The EV particle/protein ratio showed the same trend in total number of LEVs and SEVs across the groups (Figure 1b). Although the nanoparticle analyses showed a similar distribution of particle size in both LEVs and SEVs (Supplementary Figure 2A), TEM analysis (Figure 1d & 1e) indicated the presence of larger‐size particles ranging from 100–400 nm in size in the LEVs and a higher number of smaller‐sized particles in the range of 30–100 nm in SEVs, as expected. The TEM images showed an aggregation of particles in SEVs, which may have been counted as larger‐size particles by nanoparticle tracking analysis. Western blot analysis confirmed the presence of the well‐characterized EV‐linked tetraspanins, CD9, CD81 and CD63, in both LEVs and SEVs (Figure 1c). Interestingly, a decreased pattern of CD9 expression was observed in both LEVs and SEVs from patients with Severe disease when compared to uninfected controls, though this needs to be confirmed in a larger sample size. Other EV markers, such as Flotillin‐1, Alix and TSG101, were also present in both LEVs and SEVs. Integrin β1, commonly associated with microvesicles was observed in all four samples of LEVs. However, ApoE positivity suggested the presence of lipoproteins in the EV preparation (Brennan et al., 2020), relatively more in LEVs compared to SEVs. The GM130, another negative marker for EVs, was absent in both LEVs and SEVs (Supplementary Figure 2B).

### 3.3 Effect of plasma‐derived EVs from COVID‐19 patients on the pulmonary endothelium

**FIGURE 2:**
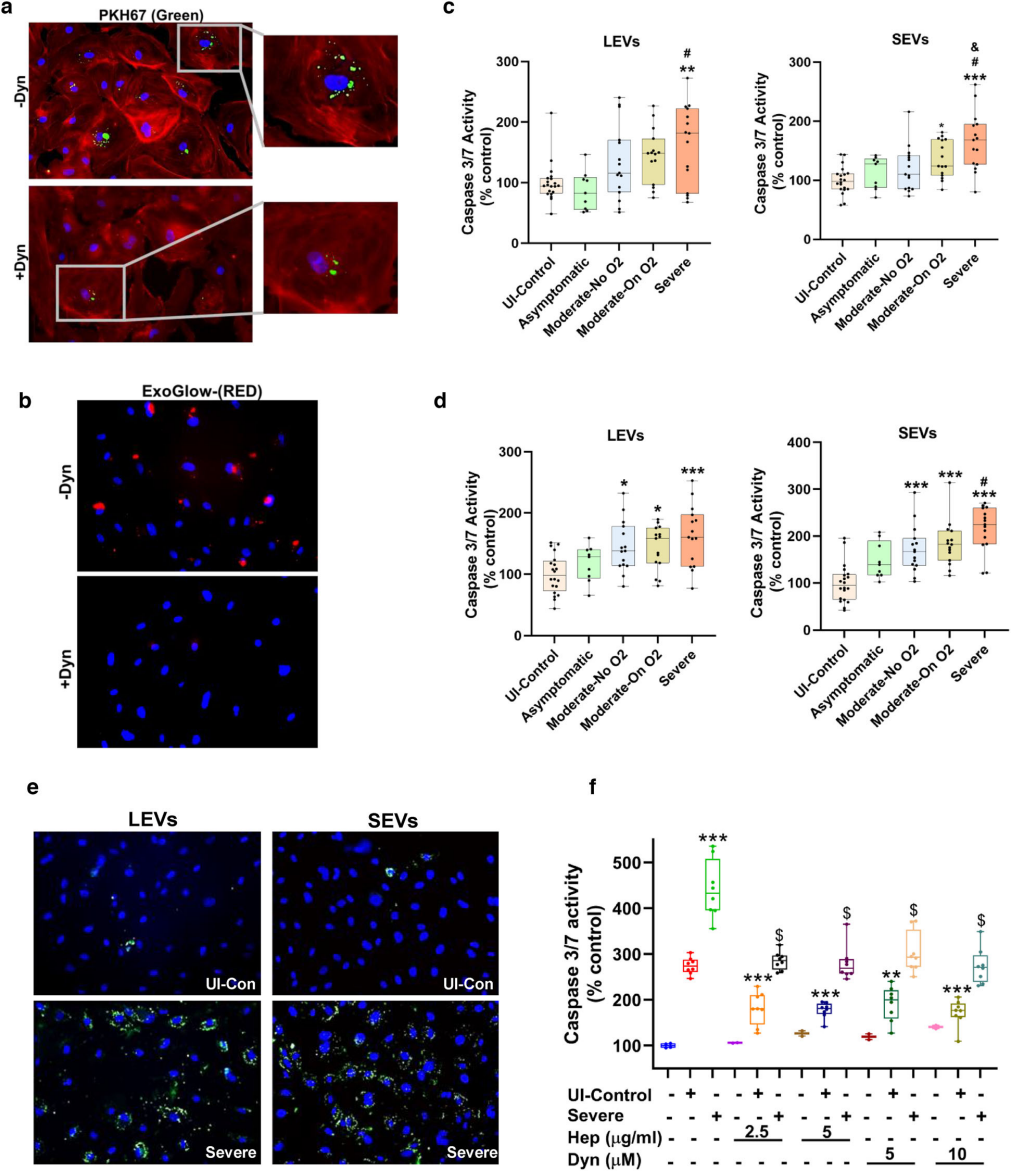
Circulating EVs from COVID‐19 patients induce endothelial apoptosis in the order of disease severity. a‐b) Uptake of PKH67 (green) (a) and ExoGlow‐Membrane (red) (b) labelled SEVs by HPMECs in the presence and absence of EV uptake inhibitor: dynasore (10 μM) After treating cells with 50 μg of labelled SEVs for 16 h, cells were fixed and mounted with DAPI containing ProLong Gold Antifade reagent. Cells treated with PKH67 labelled EVs were additionally stained with Alexa Fluor® 594 phalloidin to visualize actin filaments. Magnification 20X. c‐d) Caspase 3/7 assay on HPMECs treated with LEVs and SEVs from Uninfected Control (n = 20), Asymptomatic (n = 9), Moderate‐No O2 (n = 15), Moderate‐On O2 (n = 15), Severe (n = 15) group patient followed by Caspase 3/7 assay after 24 h. HPMECs plated in 96 well plate were treated with either EVs equivalent to 3 μg protein (c) or equal number of EVs (100 × 10^6^ EVs) (d) per well. e) Representative images of Annexin‐V staining of HPMECS treated with 10 μg of LEVs and SEVs. After 24 h of treatment, cells were stained with FITC‐ Annexin V for 15 min, washed and mounted with DAPI containing ProLong Gold Antifade reagent. Magnification 20X. f) Apoptosis analyses of HPEMCs after exposure to EDTA plasma from Uninfected Control (n = 8) and Severe COVID‐19 patients (n = 8) in the presence and absence of EV uptake inhibitors: Heparin (2.5 and 5 μg/ml) and Dynasore (5 and 10 μM). Cells were pretreated with inhibitors for 30 min followed by treatment with plasma (equivalent to 3 μg protein). Caspase 3/7 assay was later performed after 24 h. * *P* < 0.05, ** *P* < 0.01, *** *P* < 0.001 vs UI‐Control, # *P* < 0.01, vs. Asymptomatic, & *P* < 0.01 vs Moderate‐No O2. f) ** *P* < 0.01, *** *P* < 0.001 vs UI‐Control and $ *P* < 0.001 vs Severe

We next tested the effect of plasma‐derived 20K‐ and 100K‐pellet EVs on the apoptosis of pulmonary endothelial cells. For this, we first checked the uptake of EVs from COVID‐19 patients by HPMECs in the presence and absence of the EV uptake inhibitor, dynasore. Both PKH‐67 as well as ExoGlow labelled EVs were taken up by HPMECs as shown in Figure 2a & 2b, while this uptake was inhibited on pretreatment of cells with the dynasore. The addition of LEVs (equivalent to 3 μg of EV protein) from the Severe group to HPMECs resulted in significantly increased caspase 3/7 activity when compared to the treatment of cells with the same amount of EVs from the Healthy and Asymptomatic groups (*P* < 0.05). An increased trend in caspase 3/7 activity also was observed in HPMECs treated with LEVs from the Moderate group when compared with HPMECs treated with LEVs from the Healthy and Asymptomatic groups; however, this increase was not significant and was less than the increase observed in the Severe group (Figure 2c, ‐left panel). The addition of 3 μg 100K‐pellet SEVs to HPMECs also significantly increased caspase 3/7 activity in the Severe group when compared to all other groups (Figure 2c, ‐right panel). Furthermore, the comparison of adding an equal number of LEVs or SEVs (3 × 10^4^ EVs /cell) from various groups to the HPMECs also showed maximum increased caspase 3/7 activity in the Severe group (Figure 2d). Augmentation of apoptosis by LEVs and SEVs was further confirmed by FITC‐Annexin‐V staining as shown in Figure 2e.

We also determined the effect of plasma on endothelial apoptosis in the presence and absence of two EV uptake inhibitors: heparin and dynasore (Figure 2f). Heparin inhibits the endocytosis of EVs by binding to heparan sulfate proteoglycans (Franzen et al., 2014; Mrowczynski et al., 2018), while dynasore is known to inhibit clathrin‐ and caveolin‐dependent endocytosis of EVs (Chiba et al., 2018; Toribio et al., 2019). The addition of plasma samples derived from the Severe group resulted in significantly increased caspase activity compared to the plasma from uninfected controls. COVID‐19‐negative control plasma from individuals with and without comorbidities also resulted in some apoptosis when compared with untreated cells. However, the addition of plasma samples to HPMECs pretreated with heparin (2.5 and 5 μg/ml) or dynasore (5 and 10 μM) significantly mitigated the apoptosis mediated by plasma from both groups. This further confirms a role for circulating EVs in endothelial injury.

### 3.4 Analysis of LEV and SEV protein cargo for inflammatory and cardiovascular biomarkers

**FIGURE 3:**
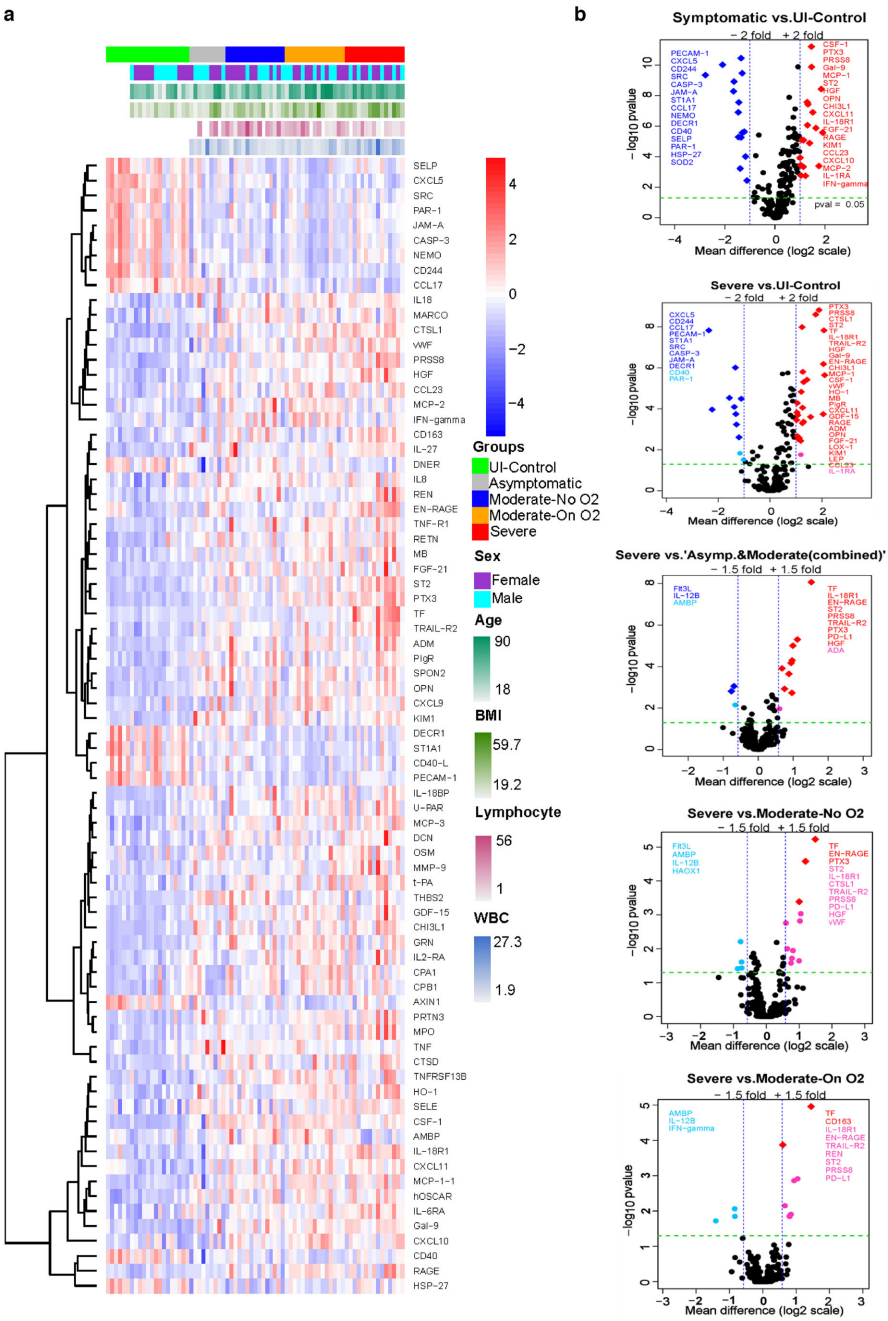
Proximity Extension Analysis (PEA) of circulating SEVs from COVID‐19 patients. EDTA plasma from Uninfected‐Control (n = 21), Asymptomatic (n = 9), Moderate‐No O2 (n = 15), Moderate‐On O2 (n = 15), Severe (n = 15) group patients was used for the comparison of inflammatory and cardiovascular related protein cargo in SEVs by employing Olink's multiplex PEA platform. a) Heat map showing hierarchical clustering of significant differences in EV protein cargo across the groups (*P* < 0.001). b) Volcano plots show pairwise post hoc comparisons of differences in EV protein cargo between various groups. The plot was constructed using −log_10_ (*P* value) against the mean difference. The green dotted horizontal line represents *P* = 0.05. The proteins with mean difference of either +/‐ 2 or +/‐1.5 fold and significance of *P* = > 0.05 are listed. In all the plots, the up‐regulated and down‐regulated proteins with FDR less than 0.05 are represented by red and blue diamond symbols, respectively whereas the upregulated and downregulated proteins with FDR greater than 0.05 are represented by red and blue circle symbol, respectively

The Olink platform that uses multi‐plex DNA‐coupled immunoassay‐based targeted proteomic approach was used to detect the inflammatory and cardiovascular related protein cargo in both LEVs and SEVs. Olink analysis was first performed on 20K‐pellet LEVs from five different groups (Healthy, n = 6; Asymptomatic, n = 6; Moderate‐No O_2_, n = 13; Moderate‐On O_2_, n = 14; and Severe, n = 9). For the analysis of 100K‐pellet SEVs we also included Uninfected (UI) controls (n = 15) with matched epidemiological and clinical characteristics to identify COVID‐19‐related alterations in EVs (UI‐controls, n = 15+6; Asymptomatic, n = 9; Moderate‐No O_2_, n = 15; Moderate‐On O_2_, n = 15; and Severe, n = 15). Supplementary Figures 3A and 4A show the Venn diagram of the number of statistically significant differentially altered proteins in LEVs and SEVs for each of the Asymptomatic, Moderate‐No O_2_, Moderate‐On O_2_ and Severe subjects with respect to healthy/un‐infected controls. The full list of altered inflammatory and cardiovascular proteins in LEVs and SEVs from all groups are presented as heatmaps showing the pattern of protein presence according to disease severity (Supplementary Figures 3B & 4B). The heatmap of differences in the protein cargo using statistical significance of *P* < 0.001 for SEVs, shown in Figure 3a, highlights alterations in proinflammatory and prothrombotic markers, as well as endothelial injury‐ and angiogenesis‐related proteins, with increasing disease severity.

Volcano plots were created to show the significant differences in EV protein cargo in the pairwise post hoc comparisons. Proteins with ≥1.5‐ or 2‐fold changes (*P* < 0.05 with and without FDR 0.05 cutoff) in SEVs are listed in Figure 3b and Supplementary Figure 3C, while proteins in LEVs are listed in Supplementary Figure 4C. Given that the 20K‐pellet LEV Olink analyses in subjects with COVID‐19 were compared with EV cargo from healthy volunteers without any comorbidities while the SEV analyses included additional uninfected controls with matched comorbidities. The comparison of differences in EV protein cargo from all symptomatic COVID‐19 patients with the uninfected healthy controls in LEVs showed a higher number of proteins with a greater than 2‐fold difference (Supplementary Figure 4C). However, the comparison of EV protein cargo in SEVs between the Severe and UI Control groups resulted in a higher number of up‐ and down‐regulated proteins that passed the FDR cutoff of 0.05 (Figure 3b). Among the proteins that were significantly altered in symptomatic patients when compared to uninfected controls, several also were altered in Asymptomatic patients admitted to the hospital for other reasons. Some of these common proteins included: follistatin (FS), human osteoclast‐associated immunoglobulin‐like receptor (hOSCAR), IL‐27 and IL‐6 receptor subunit alpha (IL‐RA) in the LEVs; and colony stimulating factor (CSF)‐1, Chitinase‐3‐like protein 1 (CHI3L1) and IL‐1 receptor antagonist (IL‐Ra) in SEVs. There also were lower levels of IL‐17 receptor A (IL17RA), CD244, CXCL5 and PECAM1 in the SEVs when compared with uninfected controls. Comparison of altered EV protein cargo in LEVs and SEVs from the Severe group to EV‐linked proteins from other COVID‐19‐positive groups (including the Asymptomatic group) showed upregulation of proteins involved in coagulopathy, inflammation, endothelial injury and tissue remodelling in the Severe group.

**FIGURE 4:**
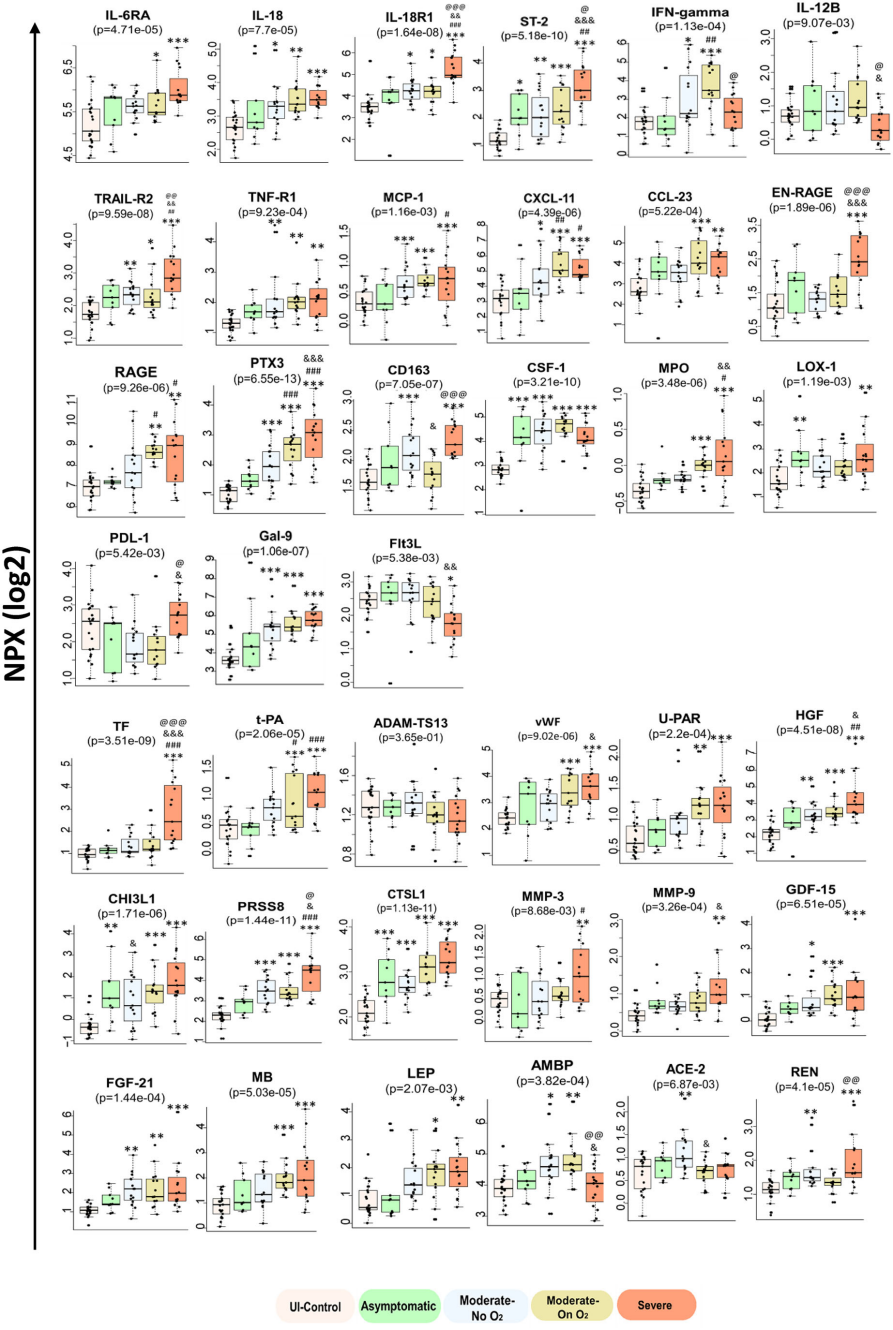
Alterations in the selected inflammation, thrombosis and tissue remodelling related proteins in SEVs indicate COVID‐19 disease severity. SEVs from Uninfected‐Control (n = 21), Asymptomatic (n = 9, WHO score 4), Moderate‐No O2 (n = 15, WHO score 4), Moderate‐On O2 (n = 15, WHO score 5), Severe (n = 15, WHO score 6–10) were analysed using Olink inflammatory and Cardiovascular II and III panels. Box‐whisker plots indicate normalized protein expression (NPX) expressed in a log2 scale, Boxes span from quartile 1 and 3 with median showing in the middle and whiskers extend to 1.5 times the IQR from the box.* *P* < 0.05, ** *P* < 0.01, *** *P* < 0.001 vs. UI‐control, # *P* < 0.05, ##p < 0.01, ###p < 0.001 vs. Asymptomatic, & *P* < 0.05, && *P* < 0.01, &&& *P* < 0.001 vs. Moderate‐No O2, @ *P* < 0.05, @@ *P* < 0.01, @@@ *P* < 0.001 vs. Moderate‐On O2

Both LEVs and SEVs were noted to be enriched in proinflammatory cytokines, including IL‐18 and IL‐18R1, whose levels correlated with increasing severity of disease in both sets of EVs (Figure 4 and Supplementary Figure 5). IL‐18R1 levels clearly differentiated patients with severe disease from others infected with COVID‐19. In addition to IL‐18R1, levels of another IL‐1R subfamily receptor, ST2 (IL‐1RL1), were higher in SEVs from patients in the Severe group. Other significant findings included higher levels of IL‐6; the IL‐6 inducer, Oncostatin‐M (OSM); and IL‐6 receptor subunit alpha (IL‐6RA), as well as IL‐8, IL‐27 and tumour necrosis factor (TNF)‐receptor superfamily proteins in both sets of EVs. Levels of TNF‐related apoptosis‐inducing ligand receptor 2 (TRAIL‐R2), also known as Death Receptor 5, were significantly higher in both LEVs and SEVs from patients in the Severe group compared to other COVID‐19‐positive patients. TNF receptor 1 (TNF‐R1) levels also increased with the severity of illness in both sets of EVs. In addition, TNF receptor superfamily member (TNFRSF) 13B and TNF ligand superfamily member (TNFSF) 13B were noted to be higher in EVs from symptomatic COVID‐19 patients. However, TNFRSF10A (also known as TRAIL‐R1 or DR4), TNFRSF10C (TRAIL‐R3) and TNFSF14 were significantly upregulated in patients with Moderate‐On O_2_ and/or Severe disease only in LEVs (Supplementary Figures 4C and 5). In parallel, TNF‐related activation‐induced cytokine (TRANCE) was significantly down‐regulated in LEVs from the Moderate‐On O_2_ and Severe groups compared to those from the Asymptomatic group. Furthermore, elevated levels of chemokines, such as MCP‐1, MCP‐3, CXCL10 and CCL23 (MIP‐3), were also found in both LEVs and SEVs from the Moderate‐On O_2_ and/or Severe groups, whereas increased levels of CXCL9 and CXCL11 were seen only in SEVs. Interestingly, levels of IFN‐γ were found to be higher in EVs from the plasma of patients in the Moderate‐On O_2_ group compared to other groups, while IFN‐γ levels were significantly lower in both 20K‐ and SEVs from the Severe group. In parallel, levels of IL‐12B, a known inducer of IFN‐γ production, were found to be lower in the Severe group compared to the other COVID‐19‐positive groups.

EN‐RAGE (extracellular newly identified receptor for advanced glycation end products binding protein), a proinflammatory molecule mainly secreted by neutrophils or monocytes, was remarkably high in LEVs and SEVs from the Severe group compared to all other groups (Figure 4 and Supplementary Figure 5). Also known as S100‐A12 and calgranulin C, EN‐RAGE acts as a damage‐associated molecular pattern (DAMP) protein that, on binding to either RAGE or TLR4 on endothelial cells or monocytes, promotes expression of cellular adhesion molecules in addition to inducing a proinflammatory axis. Interestingly, RAGE was also found to increase with severity of illness in both sets of EVs. Pentraxin‐3 (PTX3), which is associated with proinflammation and endothelial injury similar to classical pentraxin (CRP), was also significantly upregulated in SEVs in all symptomatic COVID‐19 patients with levels increasing with severity of illness.

It is important to note that circulating EVs are not only heterogeneous in size and cargo, but also in their cellular origin. CD163, a macrophage activation marker, was present at significantly higher levels in both LEVs and SEVs from the Severe group compared to those from the Moderate‐On O_2_ group. The macrophage colony stimulating factor (CSF‐1) was also significantly elevated in the Moderate and Severe groups when compared to uninfected controls; however, this elevation was lower in EVs from the Severe group compared to EVs from the Moderate‐On O_2_ group (Figure 4 and Supplementary Figure 5). We observed higher levels of granulocyte marker: carcinoembryonic antigen‐related cell adhesion molecule (CEACAM) 8, also known as CD66b, in LEVs from the Severe group but not in EVs from other COVID‐19‐positive patients. In addition, SEVs from the Severe group contained increased levels of Lectin‐like Oxidized LDL receptor 1 (LOX1) and myeloproxidases (MPOs), which are mainly associated with neutrophils. Notably, SEVs from patients with severe disease also had significantly higher levels of T‐cell exhaustion markers, PDL‐1 and Galectin‐ 9 (Gal 9), and lower levels of FMS‐like tyrosine kinase 3 ligand (Flt3L), which is known to promote the proliferation and differentiation of dendritic cells and activation of T‐cells. Finally, levels of epithelial cell adhesion molecule (Ep‐CAM) and E‐selectin (SELE) found exclusively in epithelial and endothelial cells, respectively, were significantly higher in SEVs from patients with severe disease, albeit a less than 1.5‐fold increase compared to the uninfected controls.

Significant alterations were observed in a number of proteins directly or indirectly involved in thrombosis and coagulation in both LEVs and SEVs from patients with COVID‐19. EV‐linked Tissue Factor (TF) was found to clearly differentiate patients with severe disease from all other COVID‐19‐infected groups and was invariably ranked high across all pairwise‐comparisons in both LEVs and SEVs (Figure 4 and Supplementary Figure 5). In addition to TF, tissue plasminogen activator (t‐PA) was also increased in patients with moderate and severe disease but without any significant changes in plasminogen activator inhibiter (PAI). Alternatively, ADAMTS13 (also known as von Willebrand factor (vWF)‐cleaving protease), which is an inhibitor of thrombus formation, was found to be lower in LEVs from patients in the Severe group when compared to uninfected controls (Supplementary Figure 5). Correspondingly, higher levels of vWF were noted in both LEVs and SEVs from patients on oxygen support. Furthermore, we observed higher levels of urokinase‐type plasminogen activator receptor (uPAR) in the SEVs from patients on oxygen support but not in LEVs. Membrane‐bound uPAR is known to promote fibrinolysis by activating uPA, while increased levels of soluble uPAR promote thrombosis (Mahmood et al., 2018). Hepatocyte growth factor (HGF) and Chitinase‐3‐like proteins 1 (CHI3L1) were also significantly increased in both LEVs and SEVs from patients in the Severe group compared to those in the healthy groups. In addition to being markers of inflammation and angiogenesis, HGF is associated with thrombosis and pulmonary embolism, while CHI3L1 is known to promote coagulation by inducing the expression of TF (Shan et al., 2018). Prostasin (PRSS8), a serine protease that not only regulates the epithelial sodium channel but is also expressed in endothelial and smooth muscle cells (Zhu et al., 2008), was identified as one of the most upregulated proteins in patients from the Severe group when compared to the UI Control group in both sets of EVs (Figures 3b & 4; Supplementary Figures 4C & 5). Interestingly, prostasin has recently been reported to directly or indirectly convert coagulation Factor VII to its active form (Khandekar et al., 2020).

Levels of many other proteases involved in tissue remodelling, such as cathepsin L (CTSL1), cathepsin D (CTSD), carboxy peptidases (CPA1 and CPB1) and matrix metalloproteinases (MMP9 and MMP3), were also elevated in EVs from infected patients (Figure 4). Other angiogenesis‐related proteins in addition to CH13L1, HGF and Gal9, such as growth differentiation factor 15 (GDF15), osteopontin (OPN) and fibroblast growth factor 21 (FGF21) were also noted to be increased in SEVs from COVID‐19‐positive patients. Interestingly, we also observed a significant increase in ACE2 levels in SEVs from patients in the Moderate‐No O_2_ group compared to patients in the UI Control group as well as lower ACE2 levels in EVs from patients in both the Moderate‐On O_2_ and Severe groups compared to patients in the Moderate‐No O_2_ group. This same trend was observed in LEVs, though not significant. Levels of renin (REN), another important component of the renin‐angiotensin‐aldoserone (RAAS) system, showed a higher trend in SEVs from patients with severe disease. The myocyte protein, myoglobin (MB) and adipokine leptin (LEP) were among other important upregulated proteins in EVs from patients in the Severe and/or Moderate groups, while lower levels of α1‐microglobulin/bikunin precursor (AMBP) and agouti related protein (AGRP) were seen in EVs from these same groups. These proteins could potentially be biomarkers of the cardiovascular repercussions seen in COVID‐19.

### 3.5 Correlation of significantly altered EV‐linked proteins with clinical parameters

**FIGURE 5:**
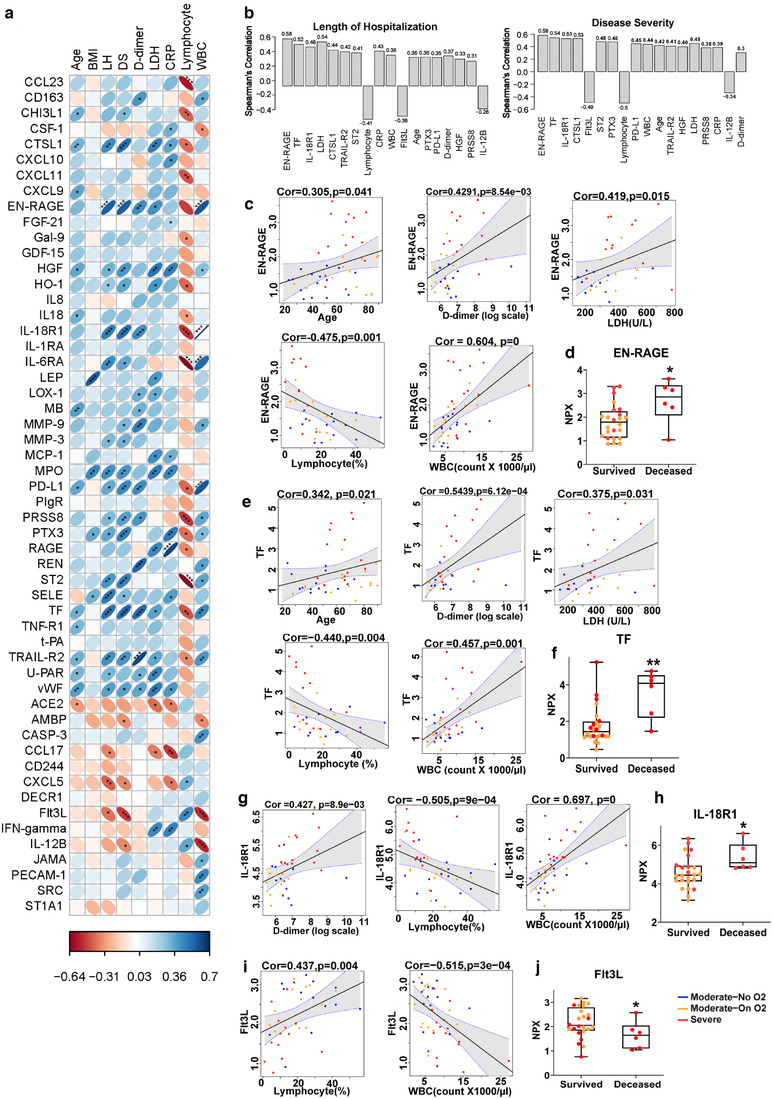
Correlation of significantly altered coagulation and inflammatory markers in SEVs from COVID‐19 patients with their demographic and clinical parameters. a) Correlogram generated from Spearman's correlation analysis represents correlation of selected significantly altered EV‐linked proteins (NPX, Log2) with age, BMI, length of hospitalization (LH), disease severity (DS) based on WHO Clinical Progression Scale score, D‐dimer (ng/ml), lactate dehydrogenase (LDH, units/L), C‐reactive protein (CRP, mg/dl) and lymphocytes (%) and WBC count (count x1000/μl) in symptomatic COVD‐19 patients (n = 45). Protein analytes are listed alphabetically with proteins that appeared at higher levels in SEVs from COVID‐19 patients shown first. The right inclined ellipse represents positive correlation and left inclined represents negative correlation. Slimmer and darker colour of ellipse represents stronger correlation (*P < 0.05, ** *P* < 0.01, *** *P* < 0.001). b) Comparison of Spearman correlation of EV‐linked proteins and clinical parameters with length of hospital stay and Disease Severity. The bars representing correlation values are arranged in increasing order of p‐values. The numbers on top or bottom of bars show absolute correlation values. Individual spearman correlation plots of EN‐RAGE (c), TF (e), IL‐18R1 (g) and Flt3L (I) showing correlation with other established parameters of COVID‐19 outcomes. Comparison of EV linked levels of EN‐RAGE (d), TF (f), IL‐18R1 (h) and Flt3L (j) in patients on oxygen support who survived (n = 24) with patients on oxygen support who died (n = 6) * *P* < 0.05, ** *P* < 0.01 vs Survived

For the analysis of the correlation of proteins with clinical lab parameters, we included only symptomatic patients due to lack of complete clinical data for most asymptomatic patients. Correlation matrices shown in Figure 5a and Supplementary Figure 7A were generated by performing Spearman's correlation analysis of the significantly altered proteins in the LEVs and SEVs by age, BMI, LH, disease severity and clinical parameters. As mentioned in the statistical methods section, to avoid the effect of two extreme observations in the LoH on correlation, two patients from the Severe group with 77 and 99 days of hospitalization stay were excluded from the Spearman's Rank correlation analysis. Nevertheless, excluding these observations did not alter the results or conclusions obtained from the analyses when including all observations (data not shown). The right‐inclined blue ellipses in the correlogram represent the positive correlation while the left‐inclined ellipses represent the negative correlation of some of the selected proteins. The shape of the ellipse and intensity of the colour represents the strength of the correlation. Thinner and darker ellipses represent higher correlation. The higher levels of IL‐6, CXCL‐10 and MCP‐3 in LEVs from symptomatic COVID‐19‐infected patients appeared to have the strongest correlation with LDH and CRP (Supplementary Figures 7D, E & G), while levels of CTSL1, EN‐RAGE, HGF, IL‐18R1, TF and TRAIL‐R2 showed significant positive correlation with LH and disease severity in both LEVs and SEVs. Additionally, EN‐RAGE, TF and TRAILR2 had a strong positive correlation with D‐dimer, while HGF and CTSL1 positively correlated with CRP along with all showing positive correlation with age, and LDH in SEVs. Of these markers, EN‐RAGE, followed by TF and IL‐18R1, showed the highest correlation with disease severity and length of hospitalization compared to other significantly altered proteins in SEV cargo. To state differently, this correlation was stronger than the correlation of LDH, CRP, D‐Dimer, or age (all of which are clinical or laboratory parameters often tracked during hospitalization) with disease severity or length of hospitalization (Figure 5b). Furthermore, levels of EN‐RAGE, TF and IL‐18R1 in SEVs from baseline blood draws (collected, on average, 10 days from symptom onset) were significantly higher in patients who died compared to those on supplemental oxygen (Moderate‐On O_2_ and Severe groups) who survived (Figures 5c‐h).

PD‐L1, significantly upregulated in SEVs from critically ill patients, showed positive correlation with age, D‐dimer, length of hospitalization and disease severity. In addition to TF, most other EV‐linked coagulopathy markers, including vWF, uPAR and ADAMTS13, also correlated with D‐dimer, length of hospitalization and age in both LEVs and SEVs. However, the strongest correlation with D‐dimer was observed with TRAIL‐R2 in SEVs. Many other molecules in SEVs such as ST2, PRSS8, CD163, IL‐18R1, IL‐6RA, RAGE, MB, MMP3 and MPO that showed a positive correlation with length of hospitalization did not show any correlation with age. Leptin, MPO, PTX3 and tPA were the only proteins from these selected significantly altered EV signatures that correlated with BMI. Finally, higher levels of most of EV‐linked markers in both LEVs and SEVs were negatively correlated with lymphocyte count and positively correlated with WBC count. WBC and lymphocyte counts showed the strongest correlation with IL‐18R1 and ST2, respectively, in SEVs from symptomatic COVID‐19 patients (Figure 5a, Figure 5c and Supplementary Figure 6C). Conversely, the lower levels of FIt3L and IL‐12B in SEVs from patients with severe disease were negatively correlated with WBC count, peak score and length of hospitalization but positively correlated with lymphopenia (Figure 5i and Supplementary Figure 6H). The levels of Flt3L and IL‐12B in SEVs could segregate patients on oxygen support who survived from the patients on supplemental oxygen who later died (Figure 5j and Supplementary Figure 6P).

### 3.6 Tissue factor activity analysis of LEVs and SEVs

**FIGURE 6:**
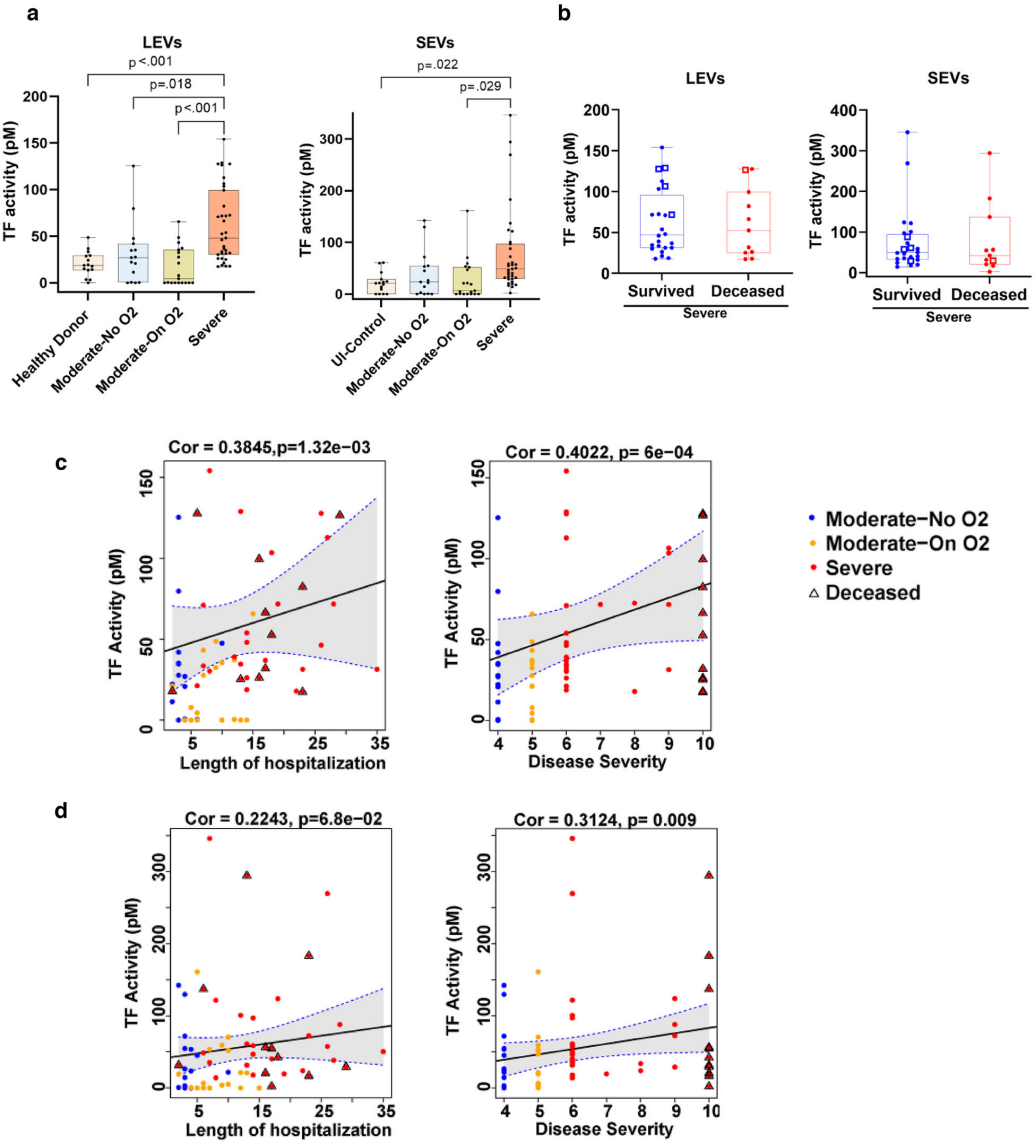
Tissue Factor activity in circulating EVs increases with increase in COVID‐19 disease severity. a) Both LEVs and SEVs from Uninfected Control (n = 15), Moderate‐No O2 (n = 15), Moderate‐On O2 (n = 19) and Severe (n = 35) groups were analysed for TF activity using AssaySense human Tissue Factor (TF) chromogenic activity kit. b) Comparison of EV‐TF activity between deceased and survived patients from the ‘Severe’ group with WHO score ≥6 (n = 35). The square represents patients with a clinically evident pro‐thrombotic state. c‐d) Spearman's correlation of TF activity linked to LEVs(c) and SEVs (d) with length of hospitalization and disease severity of COVID‐19 patients. For correlation two extreme length of hospitalization observations were excluded. The triangle represents deceased patients

TF has previously been reported to be associated with microparticles or larger vesicles in many studies (Bern, 2017; Nielsen et al., 2018), but only a few groups have reported significant presence of TF in smaller vesicles or SEVs (Che et al., 2017). Therefore, we proceeded to confirm our Olink findings by measuring the TF activity in both sets of plasma‐derived EVs isolated from all patients included in the Olink analyses, as well as an additional 20 patients with severe disease and 4 patients in the Moderate‐On O_2_ group. As depicted in Figure 6a, TF activity in LEVs from patients in the Severe group was significantly increased compared to TF activity in EVs from the UI Control group and patients with moderate COVID‐19 disease. Similar to our Olink findings, patients in the Severe group also showed increased TF activity in SEVs when compared with COVID‐19‐negative controls. Interestingly, within the Severe group no significant differences in EV TF activity were observed in patients who later died of COVID‐19 complications compared to patients who survived (Figure 6b). Specifically, EV‐TF activity levels were markedly higher in LEVs from five patients (four survived and one died) who demonstrated clinical signs of a prothrombotic state with the development of strokes, VTE, splenic infarct and/or vision changes (cotton wool spots on ophthalmologic exam) during their hospitalization. The increased TF activity in LEVs showed higher positive correlation with disease severity and length of hospitalization than SEVs (Figures 6c‐d).

### 3.7 Analyses of TF and EN‐RAGE levels in EVs isolated by size‐exclusion chromatography

**FIGURE 7:**
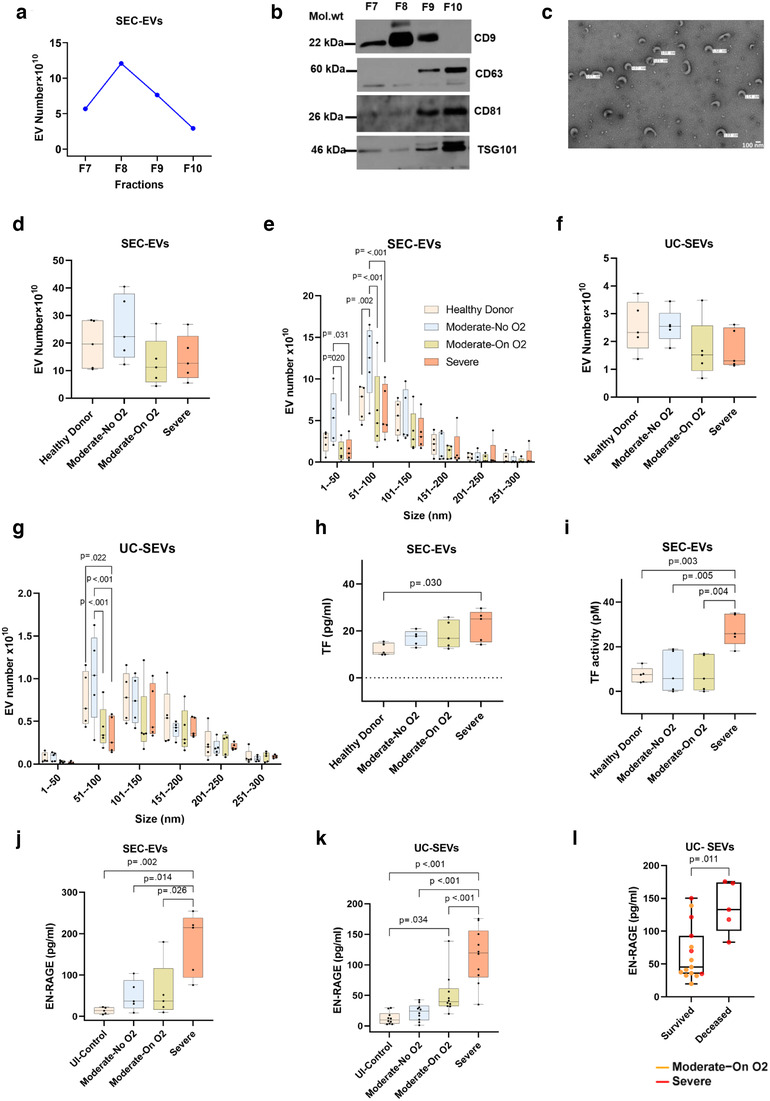
Analysis of Tissue factor and EN‐RAGE levels in EVs isolated using size –exclusion chromatography (SEC). Extracellular vesicles were isolated using 0.5 ml of EDTA‐plasma from Moderate‐No O2 (n = 5), Moderate‐On O2 (n = 5) and Severe (n = 5) group COVID‐19 patients as well as un‐infected healthy controls (n = 5). Platelet free plasma was centrifuged at 20,000 g for 15 min to remove LEVs and supernatant was then loaded on Izon's qEV original columns followed by collection of fractions. a) Total particle number in each individual fraction (fraction #7‐10) using nanoparticle tracking analysis. b) Western blot analysis of EV markers in individual fractions. c) Representative TEM image of SEC‐EVs. d‐e) Total particle numbers (d) and size distribution of EVs (e) in 7–10 pooled fractions across groups. f‐G) Total number (f) and size distribution (g) of SEVs when isolated using ultracentrifugation method (UC‐SEVs) from the same set of individuals. h‐i) Tissue factor protein levels (h) Tissue factor activity (i) in SEC‐EVs. j‐k) EN‐RAGE levels in j) SEC‐EVs and k) UC‐EVs. l) Comparison of EV linked EN‐RAGE levels between patients on oxygen support who survived (n = 15) and who died (n = 5)

We independently validated the findings of SEV‐linked increased levels of TF and EN‐RAGE in EVs isolated using IZON size exclusion columns (SEC‐EVs) from randomly selected samples (n = 5/group). As illustrated in Figure 7a, the particle number started increasing from fraction 7, reaching a maximum in fraction 8 and then declined. The Western blot confirmed the presence of EV markers, such as CD9, CD63, CD81 and TSG101, in these fractions (Figure 7b). Furthermore, the TEM analysis of EVs isolated using the SEC column method (Figure 7c) showed that the majority of EVs fall between 50–150 nm in size. The nanoparticle tracking analysis of the pooled 7–10 fractions showed the same trends in EV number and size distribution (Figure 7d‐e) in each group as was observed for SEVs isolated by ultra‐centrifugation from the corresponding samples (Figures 7f‐g). Similar to the SEVs isolated by ultracentrifugation method, SEC‐EVs also showed significantly higher levels of TF, as well as higher functional TF activity in the Severe group when compared to the other groups (Figures 7h‐i). In addition, we performed an EN‐RAGE ELISA on the protein extract of SEVs isolated by both ultracentrifugation and SEC methods and found a significant increase in both sets of SEVs isolated from the Severe group compared to other symptomatic COVID‐19 patients and un‐infected controls (Figure 7j‐ k). Finally, similar to Olink findings ELISA findings also showed significant differences in EN‐RAGE levels between the patients on supplemental oxygen who survived from those who died (Figure 7l).

## 4 DISCUSSION

In this comprehensive study, we compared inflammatory and cardiovascular disease‐related protein cargo of EVs from the plasma of hospitalized COVID‐19 patients with varying levels of disease severity. Our results reveal enrichment of proinflammatory, procoagulation and tissue‐remodelling markers in circulating EVs, distinguishing symptomatic COVID‐19 patients from uninfected controls and delineating patients with moderate disease from the critically ill. Alterations in EV cargo correspond to enhanced apoptosis of pulmonary vascular endothelial cells. Among all proteins analysed, EN‐RAGE levels in SEVs showed the strongest correlation with length of hospitalization and disease severity. Furthermore, significantly higher TF activity in LEVs appears to distinguish patients from the Severe group from those in the Moderate‐On O_2_ group.

Extracellular vesicles play a key role in the pathogenesis of various diseases, including acute respiratory distress syndrome (ARDS), chronic obstructive pulmonary disease (COPD), pulmonary hypertension (PH) and sepsis (Mohan et al., 2020). Furthermore, EVs released by virus‐infected cells can transfer viral proteins, viral receptors and proinflammatory cargo to recipient cells, thereby contributing to the spread of viral infection and progression of tissue injury (Mack et al., 2000). In a recent study, exosomes released by epithelial cells transduced with lentivirus overexpressing SARS‐CoV‐2 genes were shown to transfer viral proteins to recipient cardiomyocytes leading to an increase in the expression of inflammatory genes (Kwon et al., 2020). SARS‐CoV‐2 enters cells via the binding of its spike protein to angiotensin converting enzyme 2 (ACE2) receptors leading to downregulation of ACE2 receptors and, consequently, reduced levels of soluble ACE2 (Verdecchia et al., 2020) and possibly EV‐linked ACE2. ACE‐2 loaded EVs were recently reported to prevent the infection of SARS‐CoV‐2 pseudovirus in epithelial cells (Cocozza et al., 2020). Interestingly, our OLink analysis indicated higher levels of ACE2 on circulating EVs in Moderate‐No O_2_ group patients compared to patients on oxygen support. Correspondingly, plasma renin, a hormone responsible for initiating the RAAS cascade and regulating blood pressure, was also elevated in EVs from patients with severe COVID‐19 infection compared to healthy controls.

We found that EVs from severely ill COVID‐19 patients enriched with members of the TNF superfamily and their receptors, IL‐6‐family proteins, IFN‐γ‐inducible chemokines and IL‐1‐family proteins can clearly distinguish COVID‐19 patients with severe disease from patients with moderate or mild disease. This supports previous reports of elevated circulating levels of these inflammatory cytokines and chemokines in critically ill COVID‐19 patients (Costela‐Ruiz et al., 2020; Lee et al., 2020; Leisman et al., 2020). Levels of IL‐18R1, ST2 and TRAIL‐R2 in EVs from symptomatic patients specifically correlated with increasing disease severity and LH. While delayed or impaired type I IFN signaling has been implicated in severe COVID‐19 infection, the role of the type II IFN signaling pathway is less clear with published findings showing mildly increased IFN‐γ levels in the serum and bronchoalveolar lavage fluid of patients with non‐severe infection but not in those with severe disease (Jamilloux et al., 2020; Lagunas‐Rangel & Chavez‐Valencia, 2020). Our data revealed higher levels of IFN‐γ and IL‐12B, an inducer of IFN‐γ, in the EVs from patients in the Moderate‐On O_2_ group compared to the Severe group.

Numerous reports suggest encapsulation of cytokines and chemokines in EVs with the ability to elicit biological effects in recipient cells (Barnes & Somerville, 2020; Fitzgerald et al., 2018; Giloteaux et al., 2020). The encapsulated cytokines may be released by EVs upon attaching to the recipient cell surface, thereby increasing local concentration for efficient binding to cell surface receptors and downstream signalling (Fitzgerald et al., 2018). Conversely, cytokines/chemokines may be attached to the surface of EVs, leading to cell‐specific targeting of EVs based on binding with a particular cytokine receptor (Buzas et al., 2018). TGFβ‐1 was found to be linked to the EV surface through the heparan sulfate proteoglycans (Shelke et al., 2019). We recently reported higher levels of TGFβ‐1‐loaded plasma‐derived EVs in HIV‐infected individuals with pulmonary hypertension (Krishnamachary et al., 2021) with the ability to increase smooth muscle and endothelial dysfunction. Furthermore, transferring of cytokine receptors via EVs to the vascular or uninfected cells may potentiate the spread of cytokine storm to multiple organs (Quah et al., 2008). We were able to capture higher levels of 100K‐pellet SEVs using IL‐6RA antibodies compared to IL‐18R1 antibodies as shown by flow cytometry and analysis of EV markers by Western blot (Supplementary Figure 8). IL‐18R1 is a transmembrane protein; however, IL‐6RA is present in both soluble and membrane‐bound forms (Briso et al., 2008; Gandhapudi et al., 2015). It could be that IL‐6RA attaches to the surface of circulating EVs upon release by cells (Genschmer et al., 2019).

Of all the proinflammatory markers, levels of EN‐RAGE in both LEVs and SEVs showed the strongest correlation with disease severity and LoH and clearly distinguished critically ill patients and fatal cases from those who survived. This correlation appeared even stronger than parameters currently used for prognostication, such as D‐dimer, LDH, or CRP (Terpos et al., 2020). This suggests potentially greater ability of these EV‐linked biomarkers to predict disease prognosis and progression when compared with the established predictors of COVID‐19 outcomes. In addition, in SEVs, augmented levels of the EN‐RAGE receptor, RAGE, had the strongest correlation with CRP levels among all the significantly altered EV‐linked markers. These proteins, which are implicated in lung fibrosis and remodelling and sepsis‐related ARDS, have now been reported to be enhanced in COVID‐19 disease as well (Arunachalam et al., 2020; Oczypok et al., 2017). EN‐RAGE, on binding to either RAGE or TLR4, promotes expression of proinflammatory cytokines and cell adhesion molecules in endothelial cells and monocytes via activation of the MAP‐kinase/NF‐kB signalling cascade (Ligthart et al., 2014; Oesterle & Bowman, 2015; van de Logt & Day, 2013). Therefore, EN‐RAGE‐loaded circulating EVs may be involved in a feed‐forward loop resulting in the cytokine surge and endothelial injury associated with SARS‐CoV‐2 infection.

Microvascular thromboses similar to those seen in sepsis‐induced coagulopathy and disseminated intravascular coagulopathy have been described and implicated in COVID‐19 (Connors & Levy, 2020); however, newer data suggest that a unique mechanism may promote thromboses in early COVID‐19 infection (Giloteaux et al., 2020). EVs have been shown to release or present TF and procoagulant phospholipids on their surface promoting clot formation and accelerating fibrin polymerization (Bern, 2017; Nielsen et al., 2018). The presence of EV‐linked TF in circulation has been associated with the development of venous thromboembolism (Khorana et al., 2008; Zwicker et al., 2009). We report augmented EV TF levels/activity in patients with severe disease (WHO Clinical Progression Scale score ≥5), of whom more than half received therapeutic anticoagulants in addition to antiviral therapy, steroids and/or convalescent plasma. EV‐linked TF levels significantly correlated with disease severity and LoH regardless of anticoagulation or other COVID‐19 therapy included in our analysis. Additionally, these EVs had decreased ADAMTS13 levels but were enriched in other molecules involved in haemostasis and immuno‐thrombosis: tPA, vWF, uPAR, HGF, CHI3L1 and PRSS8. Interestingly, EV‐linked TRAILR2 showed the highest correlation with D‐dimer levels among all altered coagulation and proinflammatory markers. TRAILR2 has previously been reported to be present in cancer cell‐derived EVs (Setroikromo et al., 2020) and is known to induce the release of procoagulant microparticles by endothelial cells in response to thrombin (Simoncini et al., 2009).

Even though EV TF levels may only be a fraction of total TF in circulation, EV TF is suggested to synergize with inflammation‐ and endothelial injury‐mediated increases in TF levels in overcoming the thrombotic threshold (Date et al., 2017). EV TF effects may be potentiated by adhering to neutrophil extracellular traps (NETs) and concentrating at the site of clot formation. The role of neutrophils and the dysregulated immune response in the pathophysiology of COVID‐19 and other forms of ARDS is becoming increasingly recognized (Middleton et al., 2020; Tomar et al., 2020; Zuo et al., 2020). ‘Developing neutrophils’ have recently been shown to cross‐talk with Type II pneumocytes, specifically via CEACAM8‐CEACAM6, and appear to be significantly increased in patients with ARDS. These cells express CEACAM8, MMP9 and MPO, all of which were significantly increased in EVs from the patients with severe COVID‐19 disease. Furthermore, EN‐RAGE, which was markedly increased in plasma EVs from patients in the Severe group, is also secreted by neutrophils and monocytes (Bagheri, 2017; Foell et al., 2003).

Literature suggests activated monocytes are the main source of TF‐loaded EVs (Matsumoto et al., 2017) and a key contributor to EN‐RAGE levels in circulation (Oggero et al., 2019). The higher levels of CD163, TF and EN‐RAGE in EVs from patients with severe disease compared to those from the Moderate‐On‐O_2_ group corroborate the pivotal role of monocyte/macrophages in COVID‐19 disease severity. Monocyte differentiation antigen CD14 and soluble CD163, a marker for monocyte activation, have been shown to be elevated in patients with COVID‐19 and may be potential biomarkers of disease progression (Gomez‐Rial et al., 2020; Messner et al., 2020). EVs derived from macrophages are rich in cytokines and proteases, which could cause destruction of alveolar walls and pulmonary vasculature (Cordazzo et al., 2014; Li et al., 2010, 2013; Sharma et al., 2018). Both the soluble as well as the EV‐associated forms of CD‐163 have previously been detected in the circulation of septic patients, with a predominance of EV‐associated CD163 in septic patients compared to healthy individuals (Etzerodt et al., 2017). Furthermore, T‐cell exhaustion marker PD‐L1 was noted to be significantly elevated in SEVs and correlated significantly with increasing disease severity. Many studies have established the role of PD‐L1‐linked EVs in promoting an immunosuppressive environment and have suggested macrophages and dendritic cells may be a potential source of EV‐PD‐L1 (Chen et al., 2018; Ricklefs et al., 2018). Although future studies are needed to identify the cellular origins of circulating LEVs and SEVs, we speculate a pivotal role of monocyte/macrophage‐ and neutrophil‐derived EVs in COVID‐19‐related lung injury. Post‐mortem analysis of lung sections from COVID‐19 patients showed infiltration of mononuclear cells and neutrophils on haematoxylin and eosin staining, as well as endothelial cell apoptosis by caspase‐3 staining (Li et al., 2020; Varga et al., 2020). Importantly, we also show here that exposure of HPMECs to EVs isolated from patients with COVID‐19 results in apoptosis.

While our results support potential roles of circulating EVs in the pathophysiology of COVID‐19, our study is limited by its targeted approach and relatively small sample size. Given the exploratory nature of the study with limited sample size, the statistical models were not adjusted for variables such as Age, BMI, gender etc. However, we explored each of those variables separately in association with both clinical and molecular variables. Additional analyses using untargeted proteomics approach and validation using independent larger cohorts is warranted. Longitudinal analysis of EV cargo in detail will also assist in further elucidating how the roles of various EV cargo changes over time and in relation to the evolution of COVID‐19 infection. We describe the association of elevated EV proinflammatory, procoagulation and tissue‐remodelling markers with the severity of the disease. Future studies are needed to investigate the SARS‐CoV‐2‐mediated changes in the loading of EV cargo and to establish the causative role of these EVs in the disease. Although our Olink data revealed a demarcation in TF levels between COVID‐19 patients on oxygen support and those with fatal disease, there was no difference in EV TF activity observed in patients who survived or died within the Severe group. This supports recent findings that therapeutic anticoagulation does not appear to benefit or improve mortality in those with the most severe disease, warranting further study and highlighting the heterogeneity and complexity of the pathogenesis of COVID‐19. Thrombotic activity may not be the primary driver of fatal cases, but instead the combined effects of cytokine storm, immune dysregulation and endothelial injury, all of which our findings support, could play a much more pivotal role.

In conclusion, this unique study suggests a pivotal role of EVs and their cargo in the inflammation, coagulopathy and endothelial injury seen in COVID‐19 disease. Targeting a combination of several EV proteins may be more efficient than a single plasma‐derived protein when designing a biomarker or therapeutic target for this widely variable disease. These EV proteins could be used to predict prognosis in hospitalized patients and delineate hospitalization and treatment needs in outpatients with early and mild/moderate disease.

## 5 GEOLOCATION INFORMATION

University of Kansas Medical Center, 3901 Rainbow Blvd, Kansas City, KS‐66160, USA

## CONFLICT OF INTEREST

The authors report no conflict of interest.

## Supporting information

Supplementary information

Supplementary information

Supplementary information
