# Supplementary material for: Extracellular vesicle‐mediated endothelial apoptosis and EV‐associated proteins correlate with COVID‐19 disease severity
Source: J Extracell Vesicles. 2021 Jul 3;10(9):e12117. doi: 10.1002/jev2.12117 (PMC8254805; doi:10.1002/jev2.12117)
Supplement: Supplementary file 1 — Supplementary information [file JEV2-10-e12117-s002.docx]

**SUPPLEMENTARY MATERIAL**

**Extracellular vesicle-mediated endothelial apoptosis and EV-associated proteins correlate with COVID-19 disease severity**

Balaji Krishnamachary^1*^, Christine Cook^1*^, Ashok Kumar^1^, Leslie Spikes ^1^, Prabhakar Chalise^2^, Navneet K. Dhillon^1^

^1^Division of Pulmonary and Critical Care Medicine, Department of Internal Medicine, University of Kansas Medical Center, Kansas City, KS

^2^ Department of Biostatistics & Data Science, University of Kansas Medical Center, Kansas City, KS

* Authors contributed equally

Correspondence and requests for reprints should be addressed to Navneet K. Dhillon, Division of Pulmonary and Critical Care Medicine, Department of Medicine, Mail Stop 3007, University of Kansas Medical Center, 3901 Rainbow Blvd, Kansas City, KS 66160, Tel: (913) 945-6018, Fax: (913) 588-4098, Email: ndhillon@kumc.edu

**Running Title:** Extracellular vesicles and COVID-19

**Supplementary Figures and Figure Legends**

**
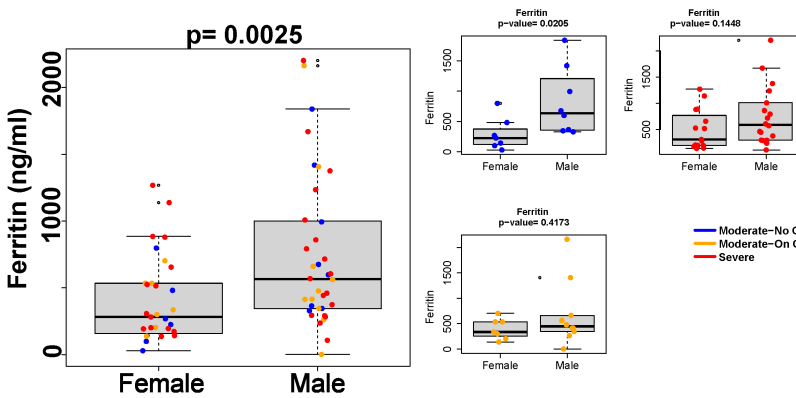

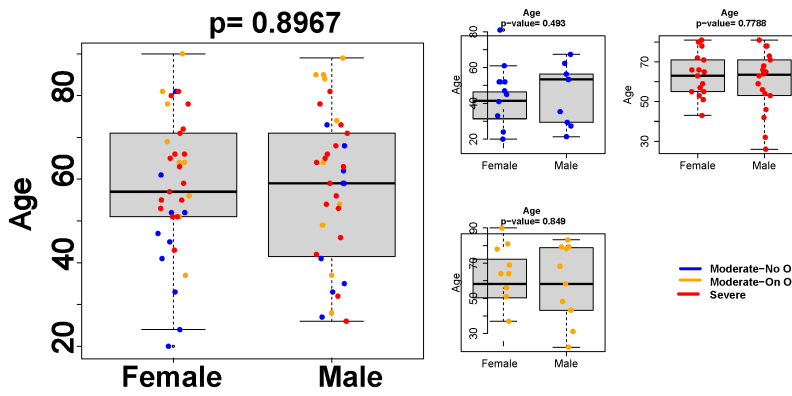

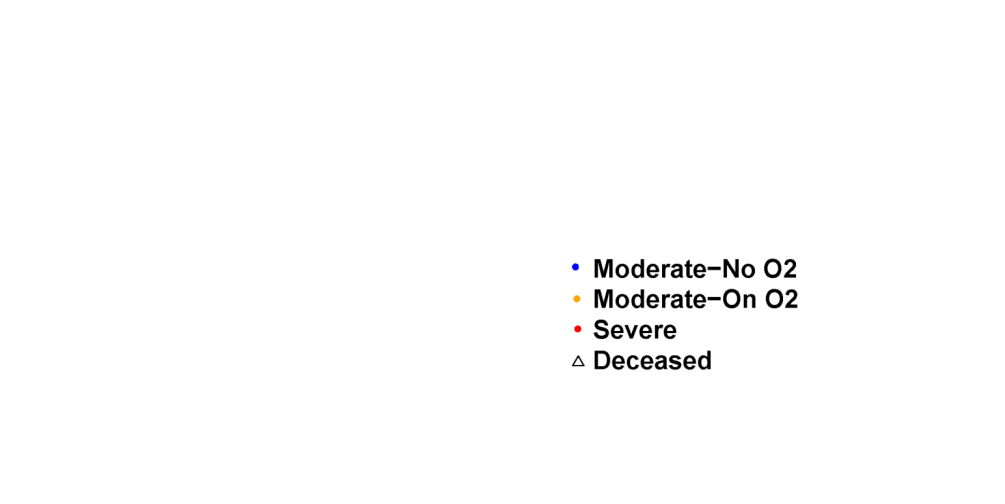

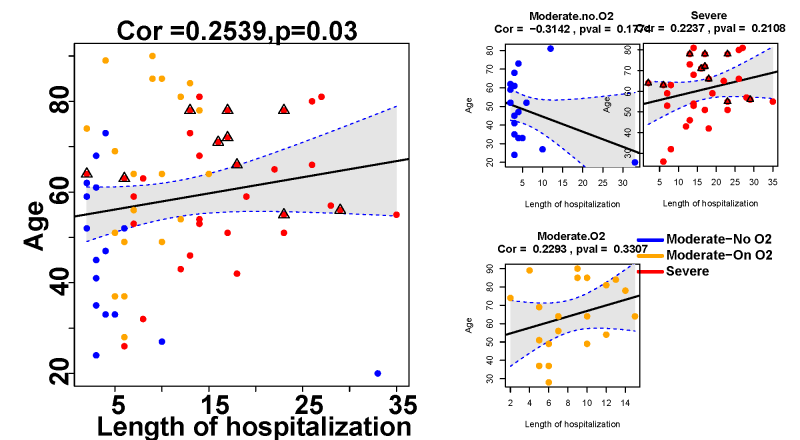

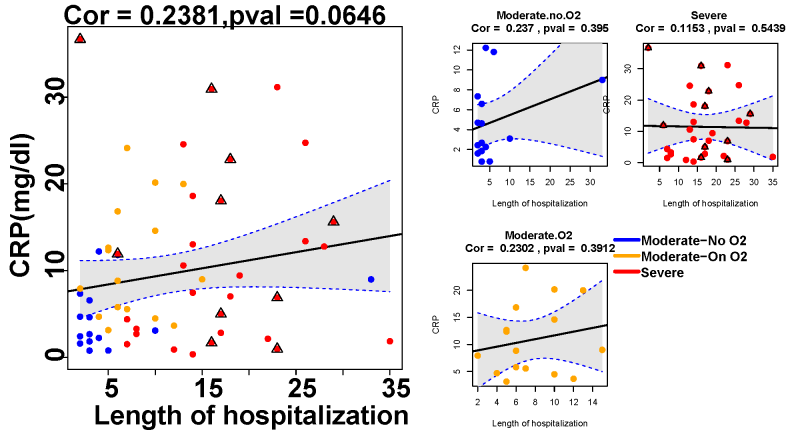

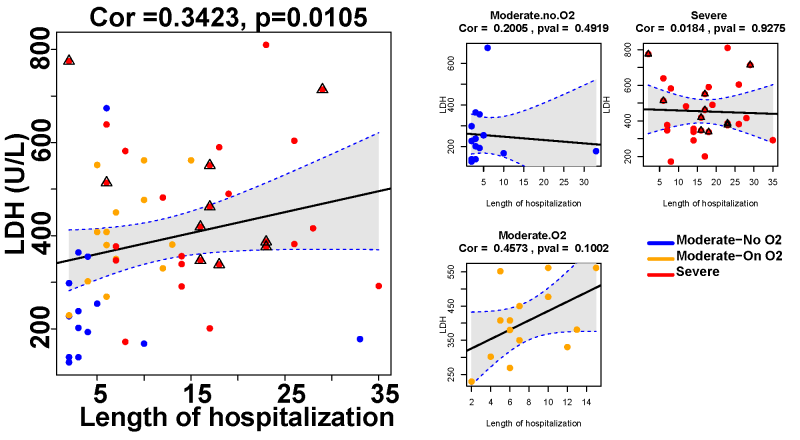

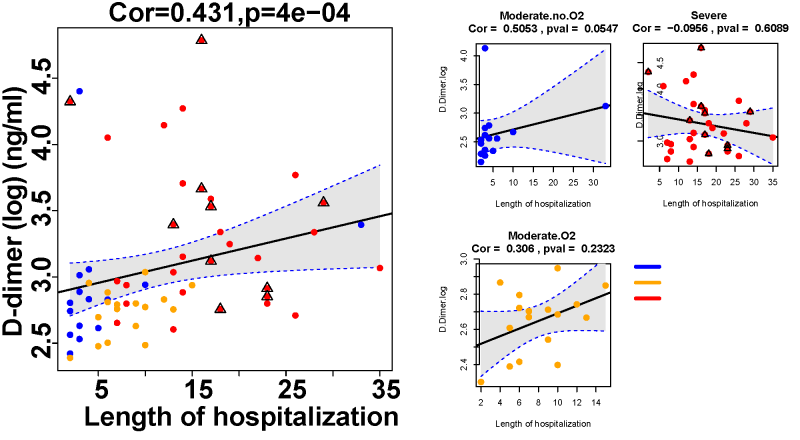

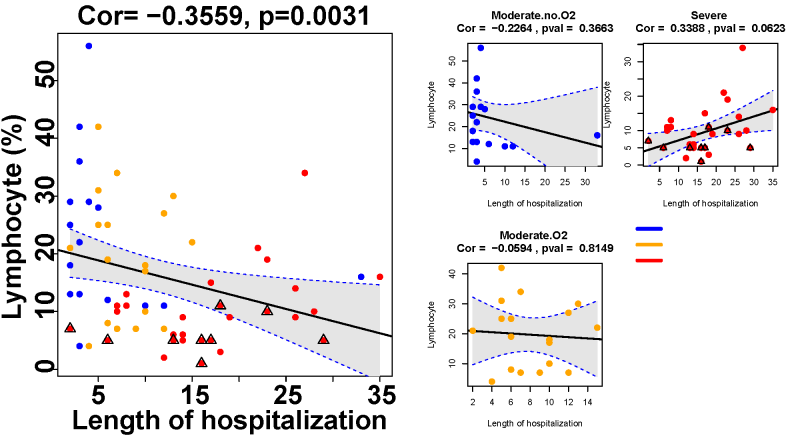

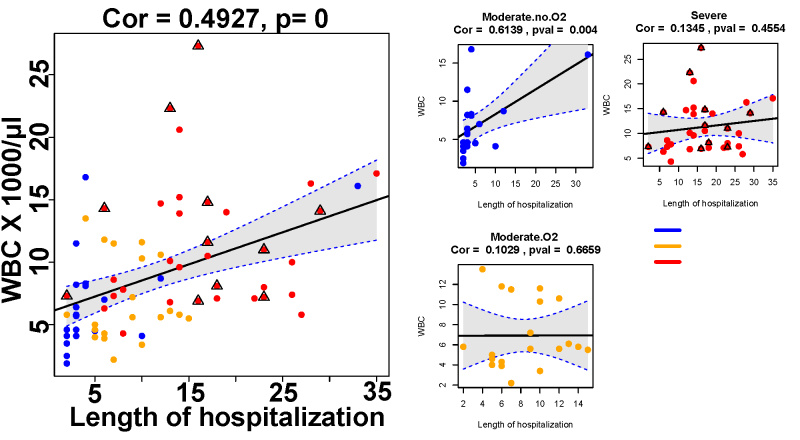

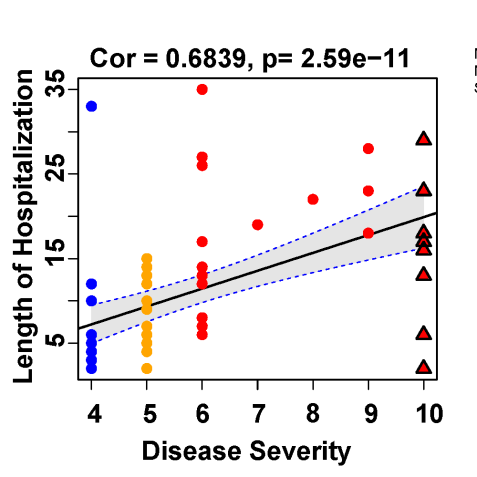
**

**A)**

**B)**

**C)**

**D)**

**Supplementary Figure 1: Correlation analysis of the demographical and clinical data of the COVID-19 patients**: For the correlation analysis, Spearman's Rank correlation was performed for the samples from Moderate-No O2 (n=20), Moderate-On O2 (n=20) and Severe (n=35) group. A) Age and other clinical parameters such as CRP, LDH, D-dimer, Lymphocyte and WBC count were significantly positively correlated with length of hospitalization B) Length of hospitalization was positively correlated with the disease severity. C) Wilcoxon Rank Sum test showed no significant difference in Age between Female and Male patients. D) Among all the clinical parameters significant gender differences was only observed in ferritin levels.


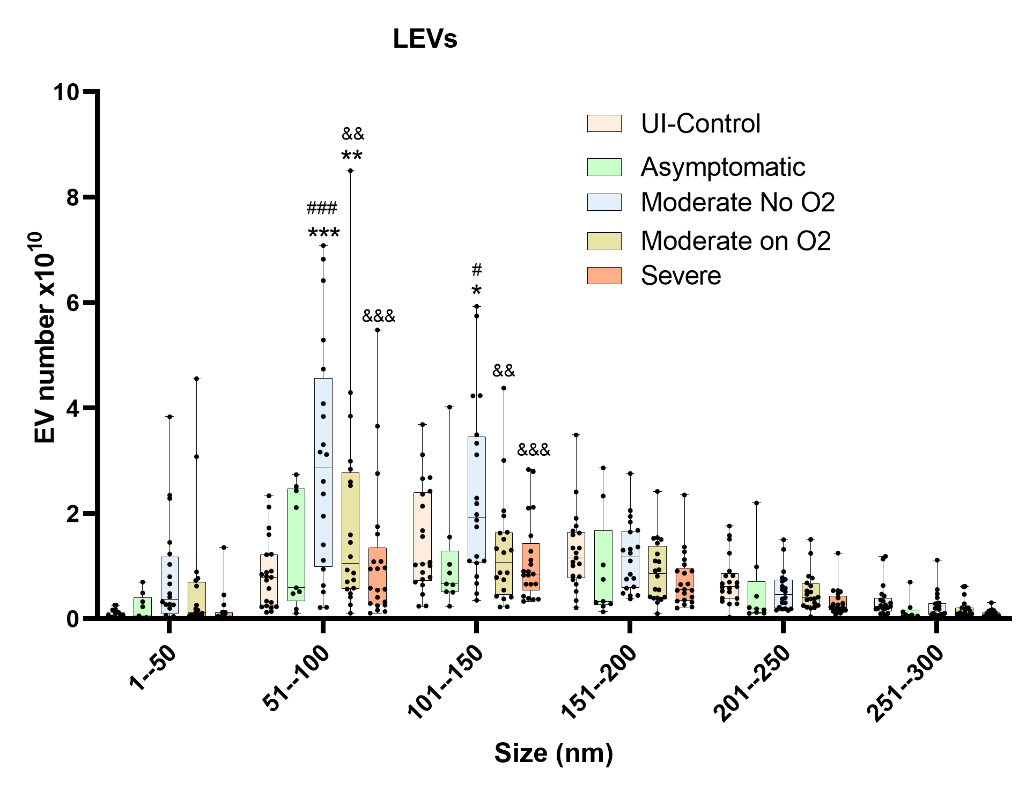


**A)**


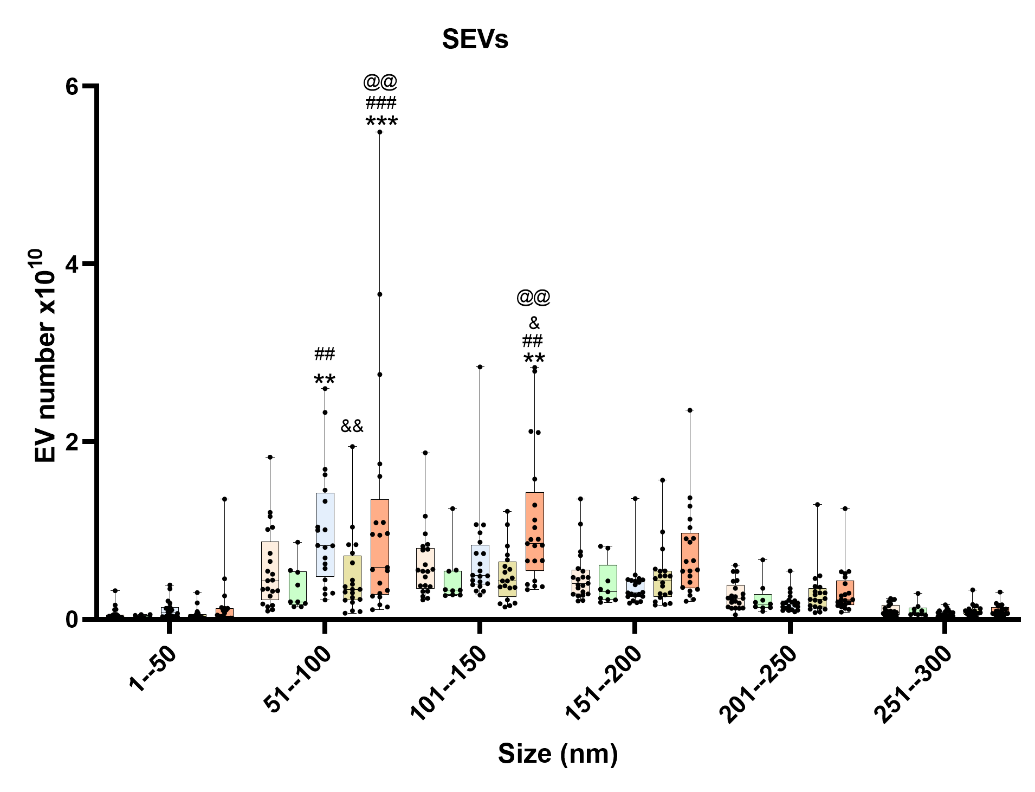


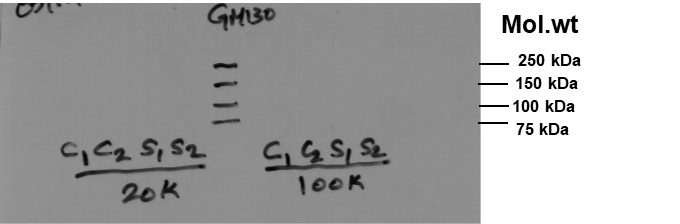


**B)**

**Supplementary Figure 2: Size distribution of LEVs and SEVs**: **A)** Particle size distribution of LEVs (Top panel) and SEVs (Bottom panel) by nanoparticle tracking analysis.Data represents number of EVs/ml of final EV suspension obtained from 1ml of EDTA plasma. Uninfected controls (n=21), Asymptomatic (n=9), Moderate-No O2 (n=20), Moderate-On O2 (n=20) and Severe (n=21). **B)** The representative western blot of LEVs and SEVs from un-infected controls (n=2) and Severe COVID-19 (n=2) patients developed using GM130 primary antibody. * p<0.05, **p<0.01, ***p<0.001 vs. UI-Control, # p<0.05, ## p<0.01, ### p<0.001 vs. Asymptomatic, & p<0.05, && p<0.01, &&& p<0.001 vs. Moderate-No O2, @@ p<0.001 vs. Moderate-On O2.


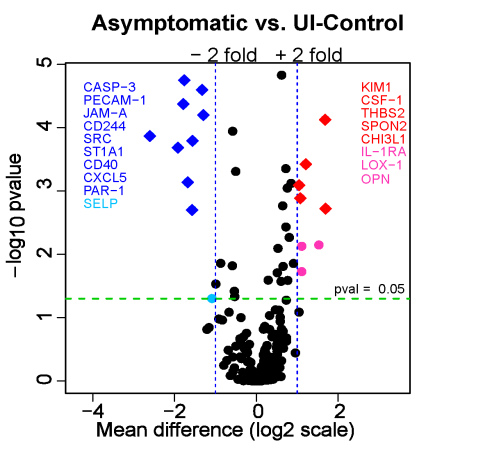

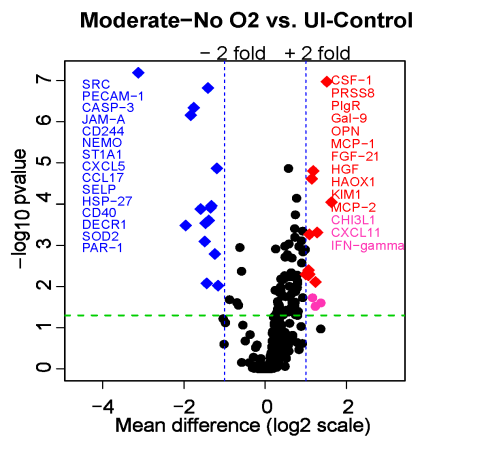

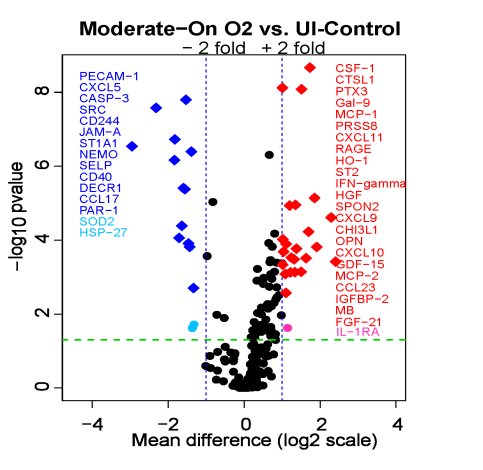

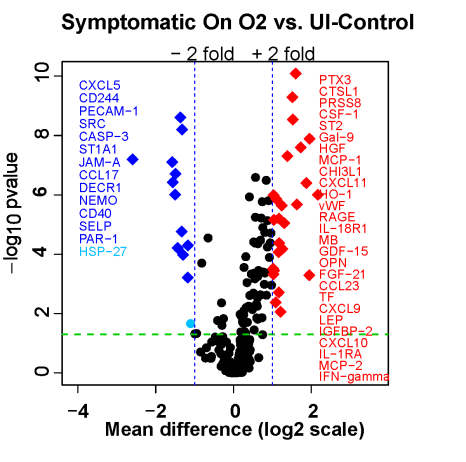

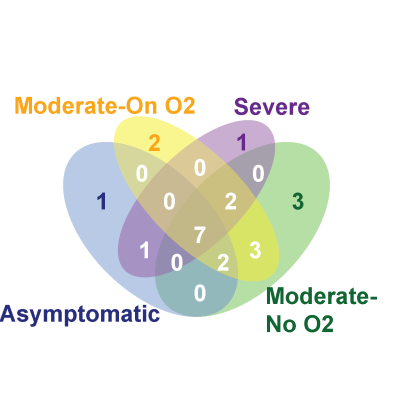

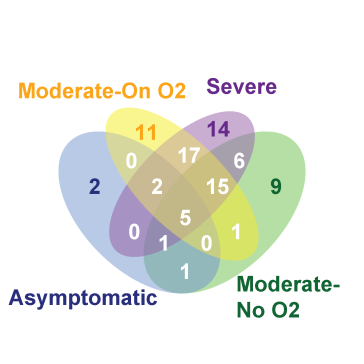

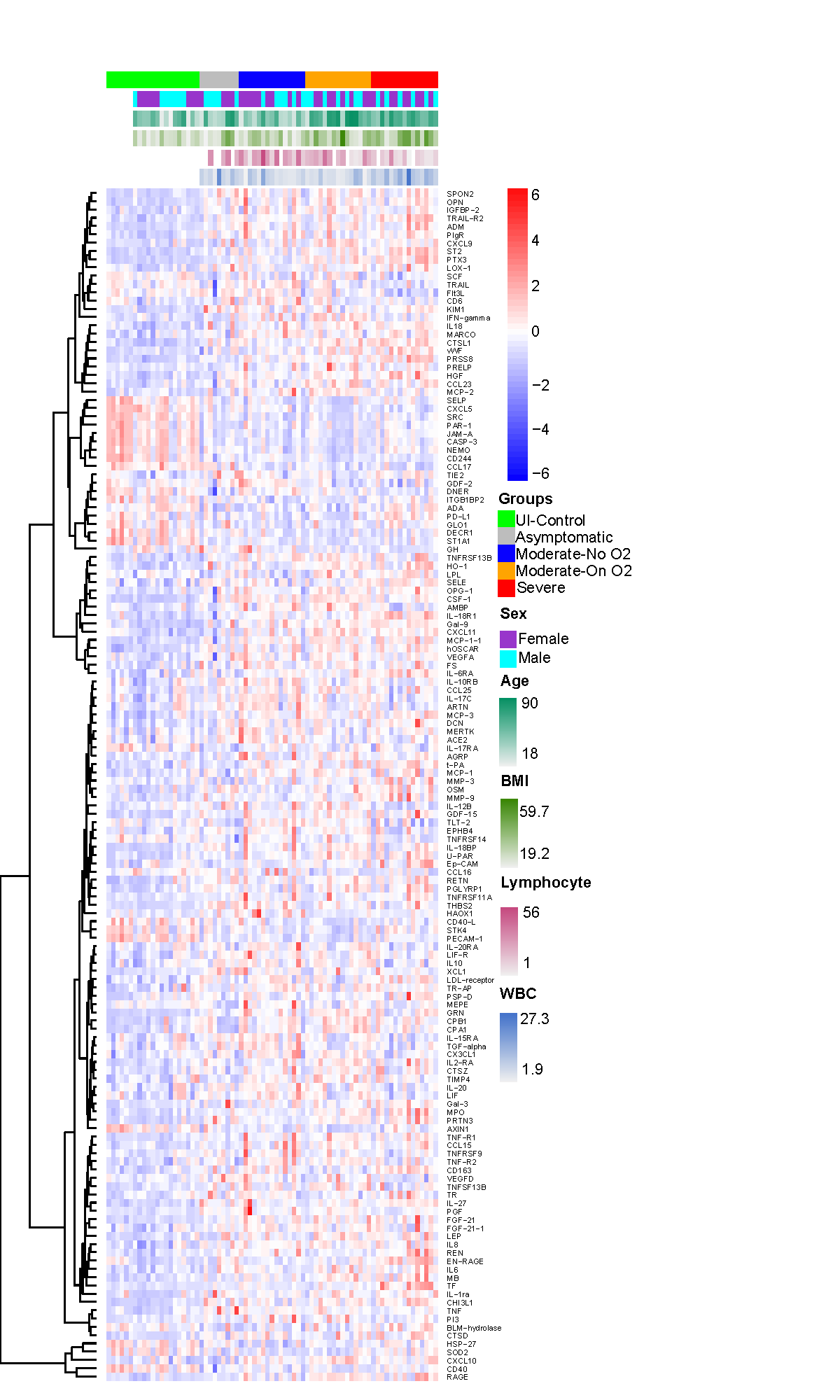


**C)**

**B**)

**A)**

**Upregulated**

**Downregulated**

**Supplementary Figure 3: Comparison of cardiovascular and inflammatory protein cargo in SEVs from COVID patients.** UI-Control (n=21), Asymptomatic (n=9), Moderate-No O2 (n=15), Moderate-On O2 (n=15), Severe (n=15) group patients were included for multi-plex Olink analyses of cardiovascular and Inflammatory cargo in SEVs. **A)** Venn diagram showing number of common and unique differentially expressed up-regulated and down-regulated proteins in different groups when compared with un-infected controls (FDR<0.05). **B)** Heat map shows the full list of differentially expressed proteins across groups (p<0.05). **C)** Volcano plots show pairwise post hoc comparisons of differentially expressed proteins in Asymptomatic, Moderate-No O2, Moderate-On O2, Severe groups when compared with un-infected controls. The green dotted horizontal line represents p=0.05. The proteins with mean difference of 2 folds and significance of p=>0.05 are listed. The up-regulated and down-regulated proteins with FDR less than 0.05 are represented by diamond symbols and proteins with FDR greater than 0.05 are represented by circles.


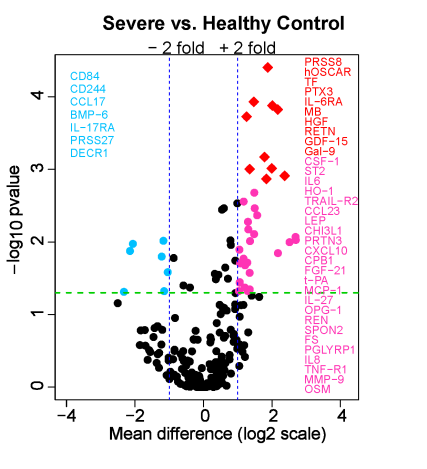

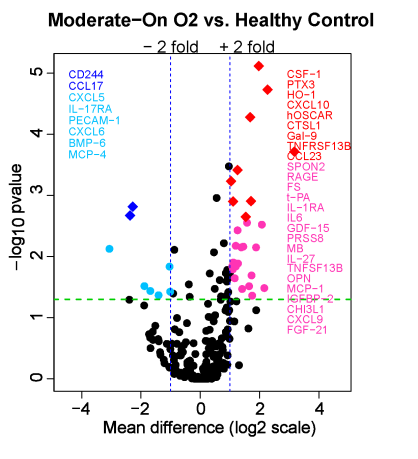

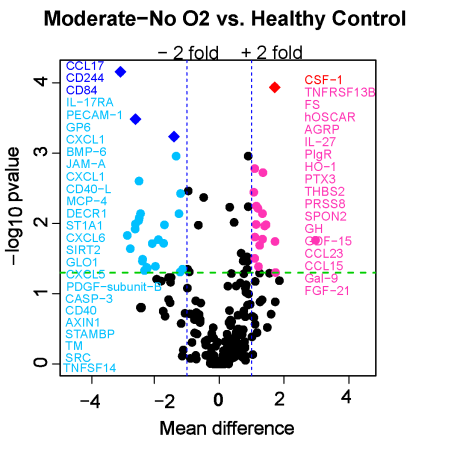

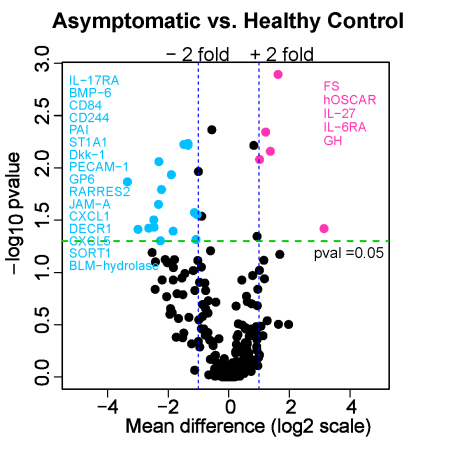

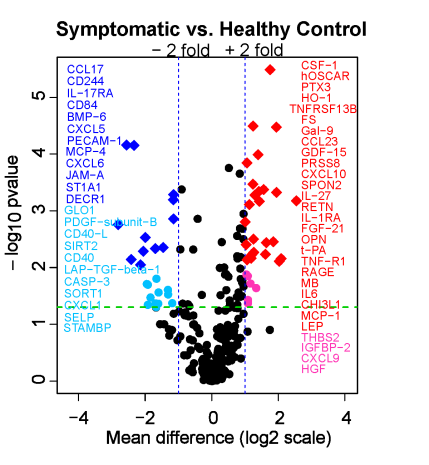

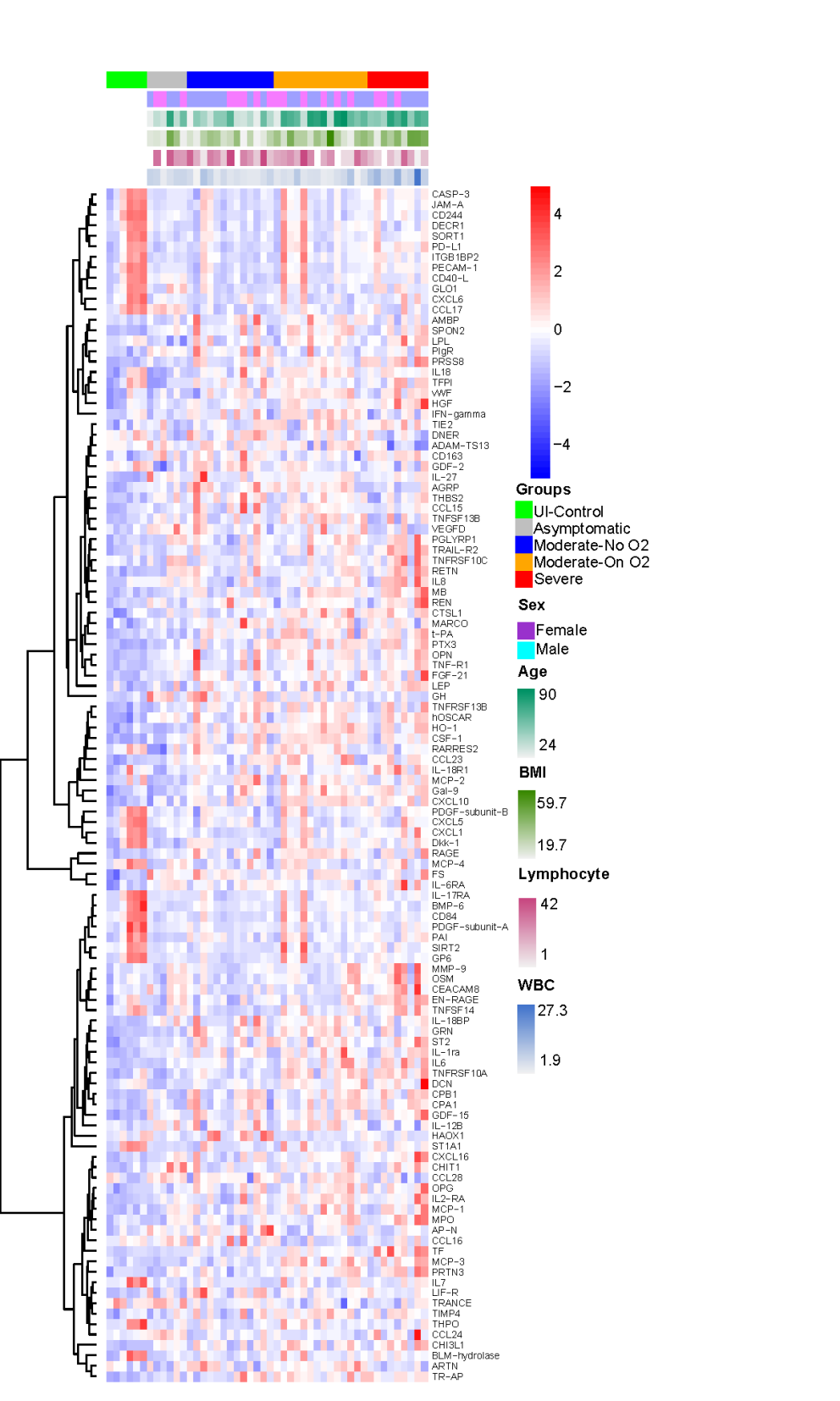

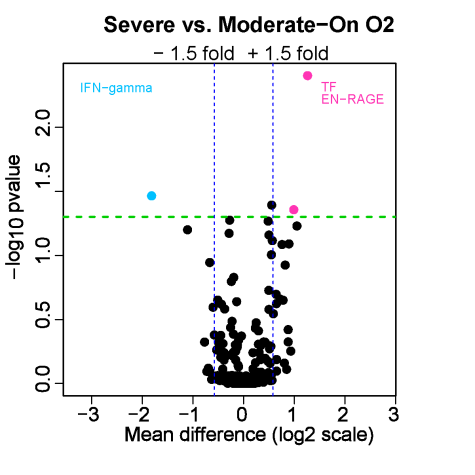

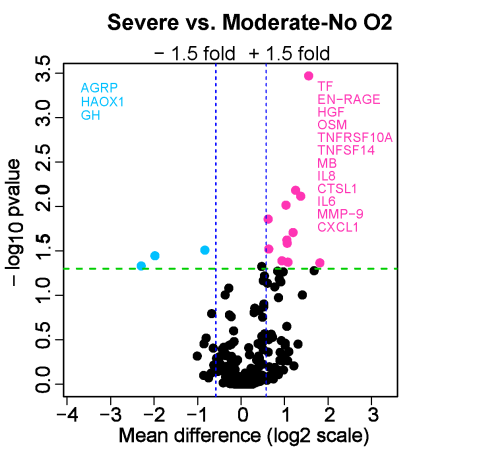

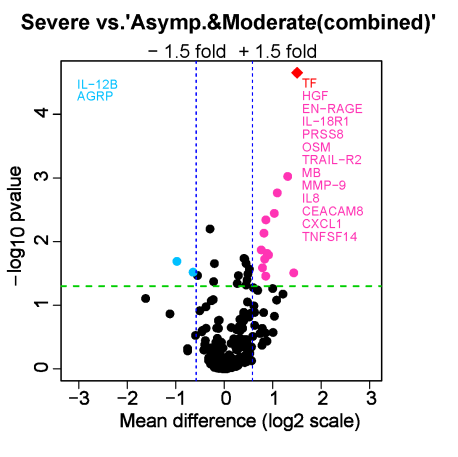

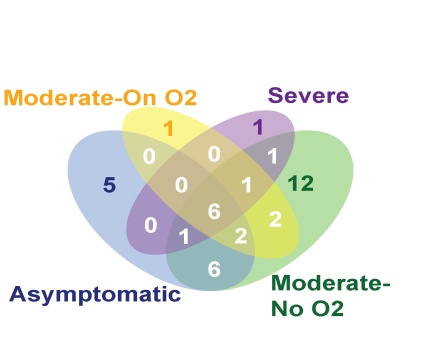

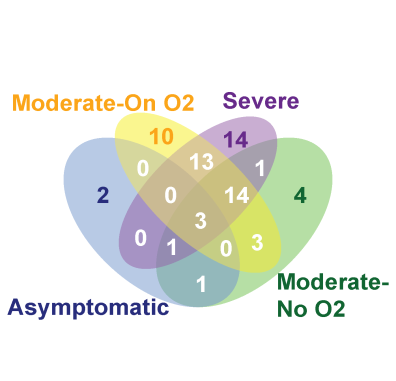


**B)**

**C)**

**A)**

**Downregulated**

**Upregulated**

**Supplementary Figure 4: Proximity Extension Analysis (PEA) of LEVs in COVID-19 patients.** LEVs from Healthy (n=6), Asymptomatic (n=6), Moderate-No O2 (n=13), Moderate-On O2 (n=14) and Severe (n=9) COVID-19 patients were analyzed using Olink multiplex PEA platform. **A)** Venn diagram represents the total number of differentially expressed common or unique proteins in different groups when compared with heathy controls (p<0.05). **B)** Heat map of hierarchical clustering of all differentially expressed proteins (p<0.05). **C)** Pairwise post hoc comparisons of differentially expressed proteins in various groups. The volcano plot was constructed using −log_10_ (p value) against the mean difference. In all the plots, the downregulated and up-regulated proteins (>= +/-1.5 or +/-2 fold, P<0.05) with FDR less than 0.05 are represented by blue and red diamond symbols, respectively, while proteins with FDR > 0.05 are represented by circle symbols.


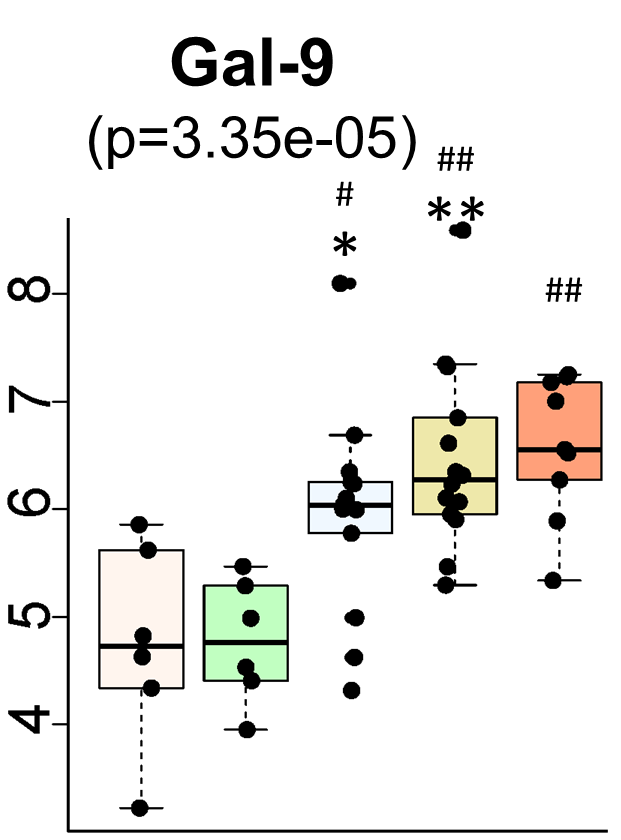

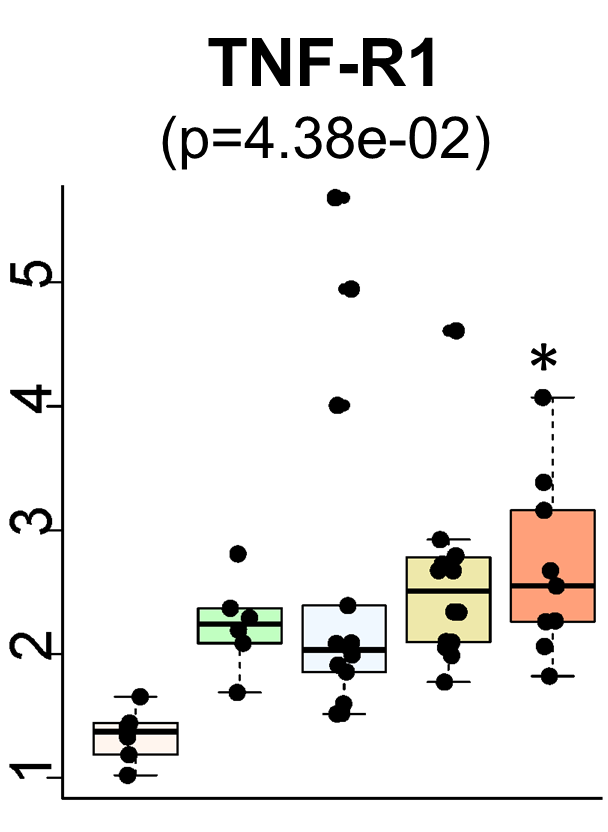

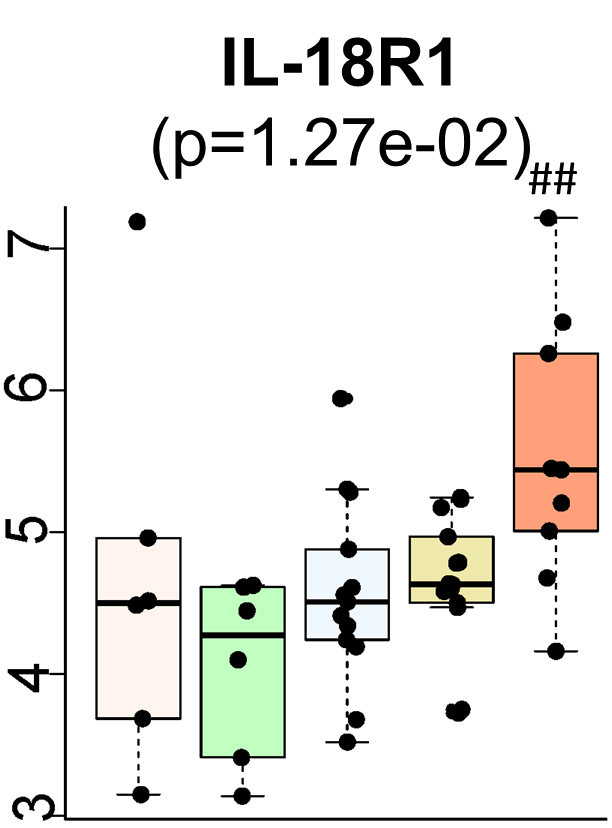

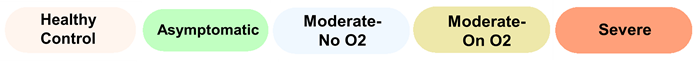

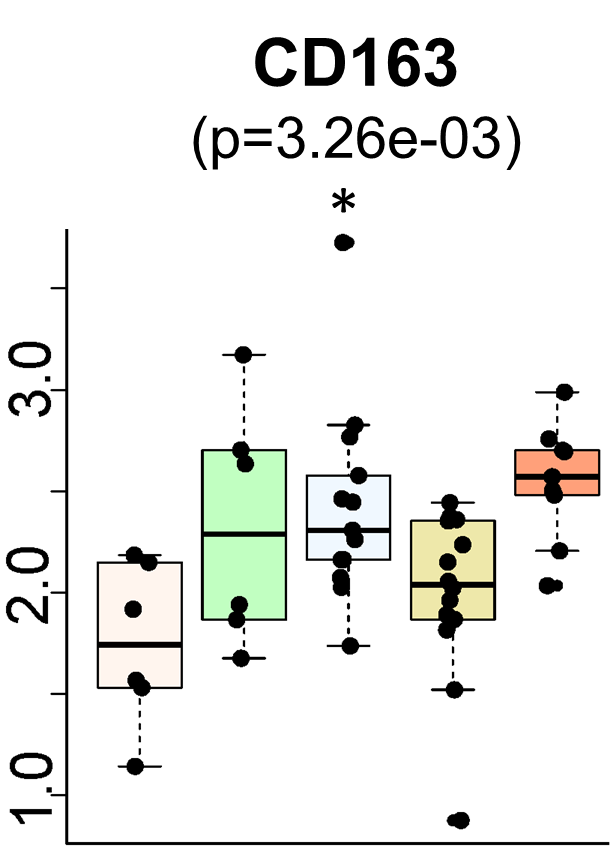

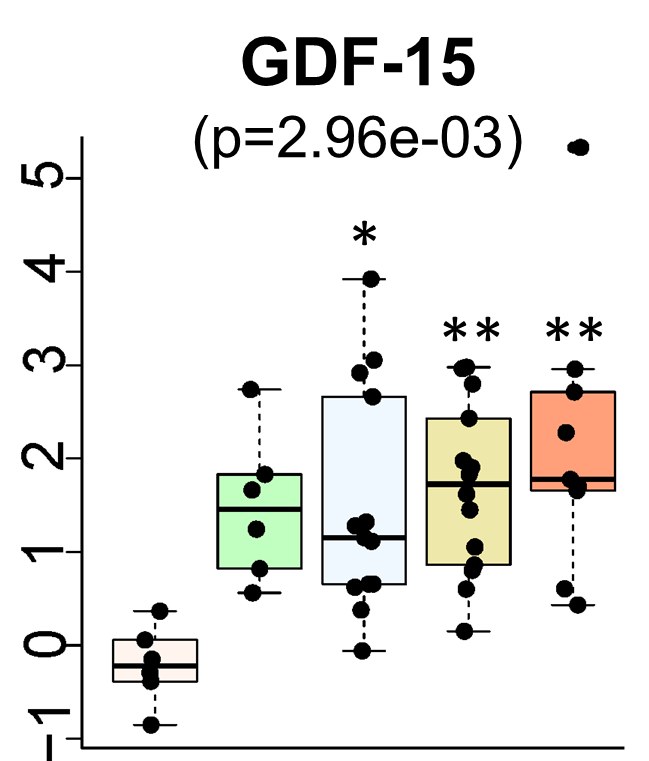

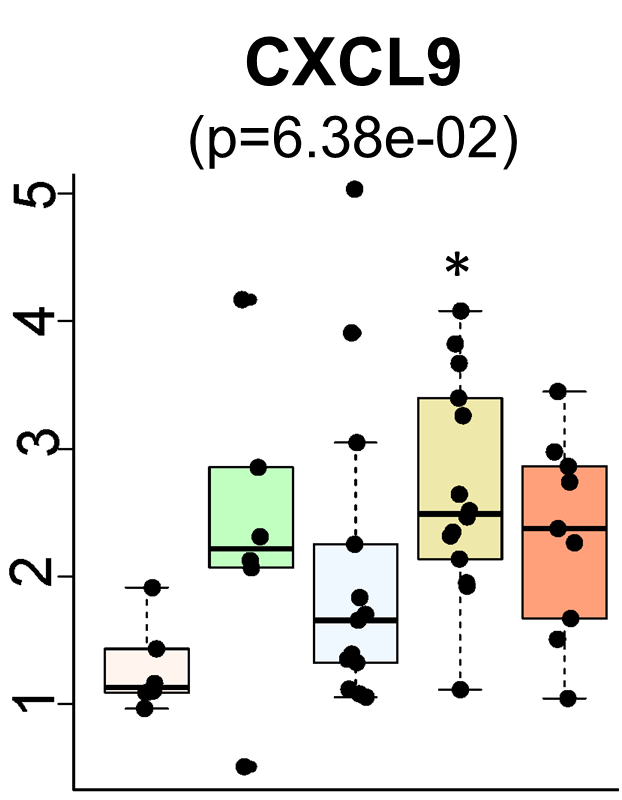

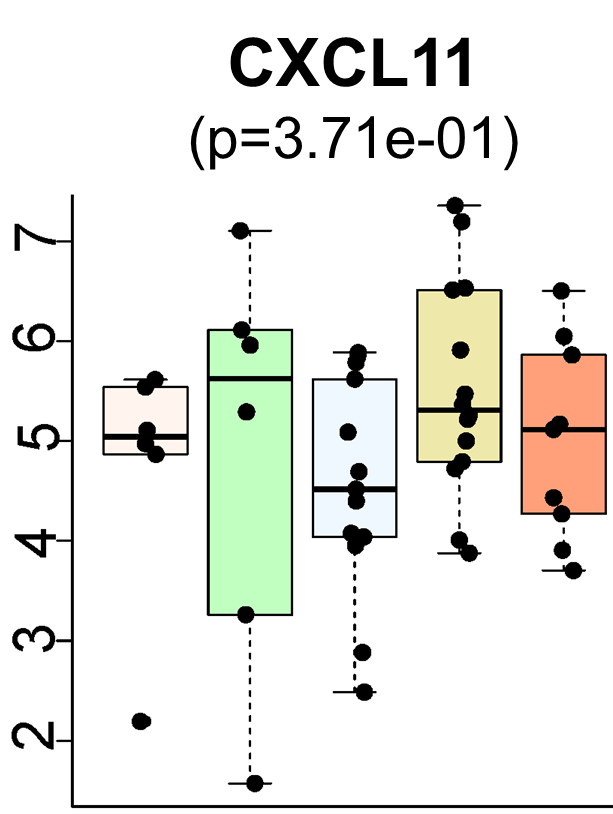

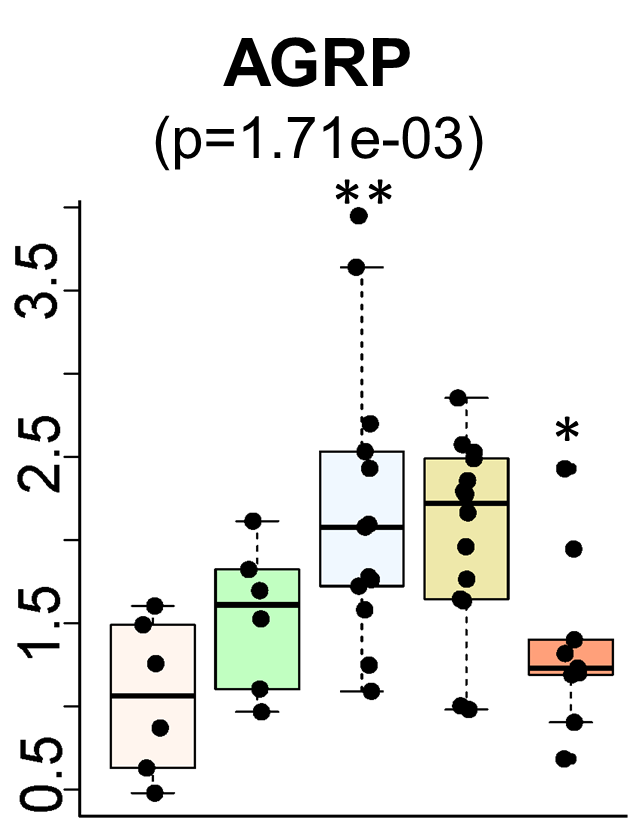

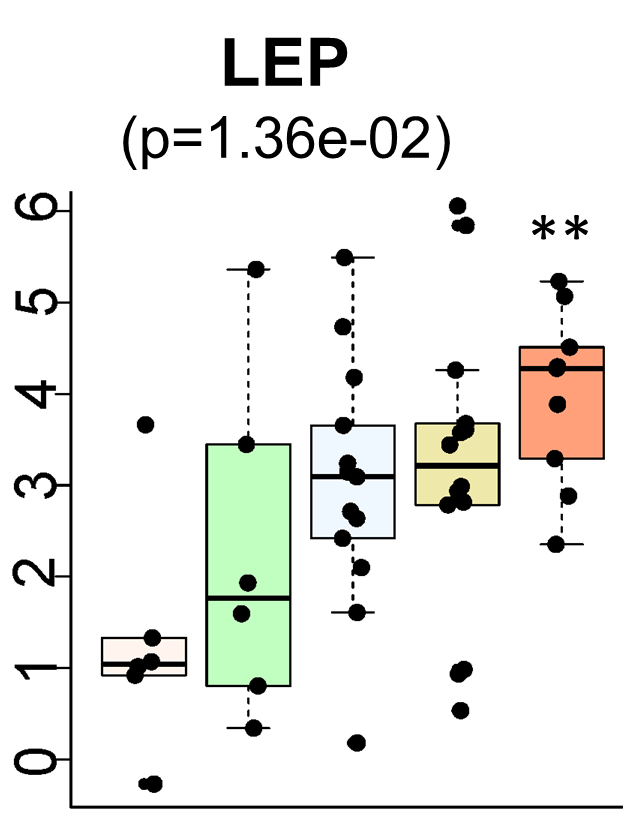

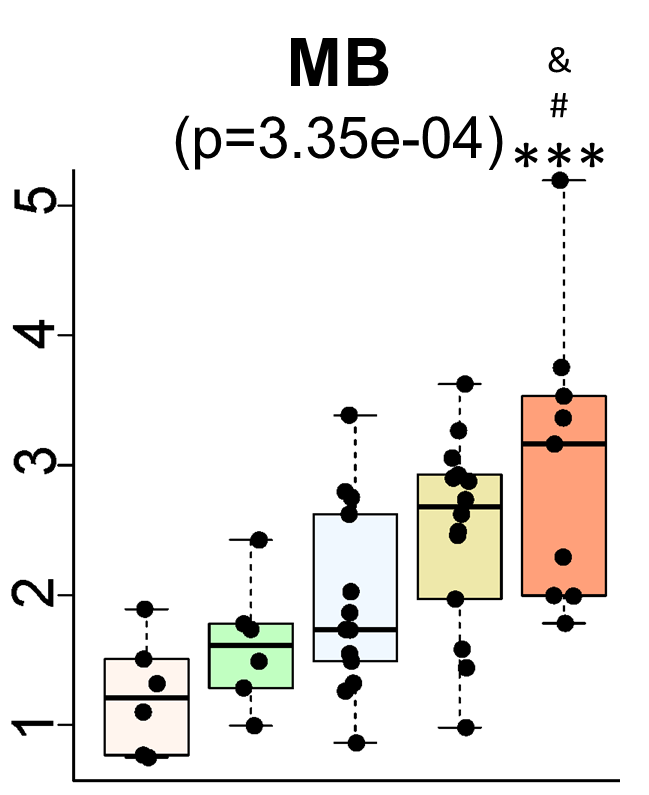

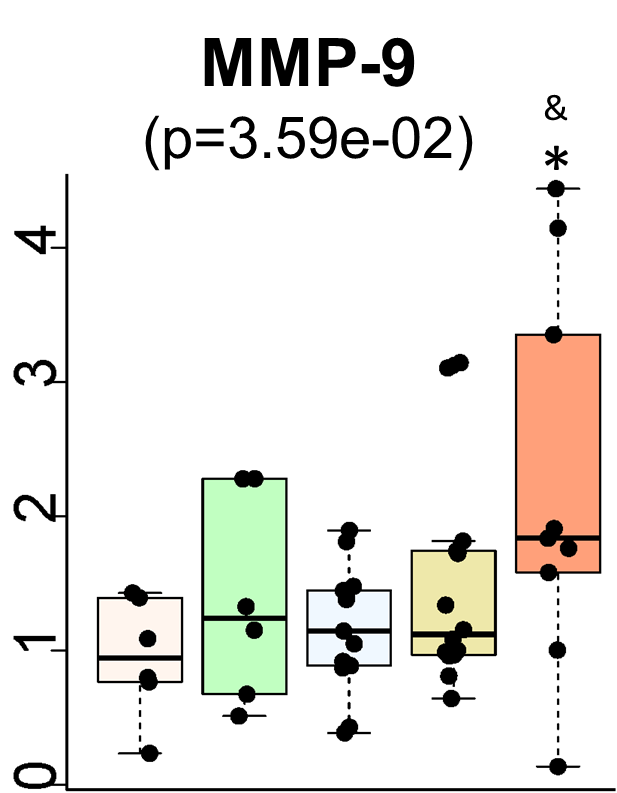

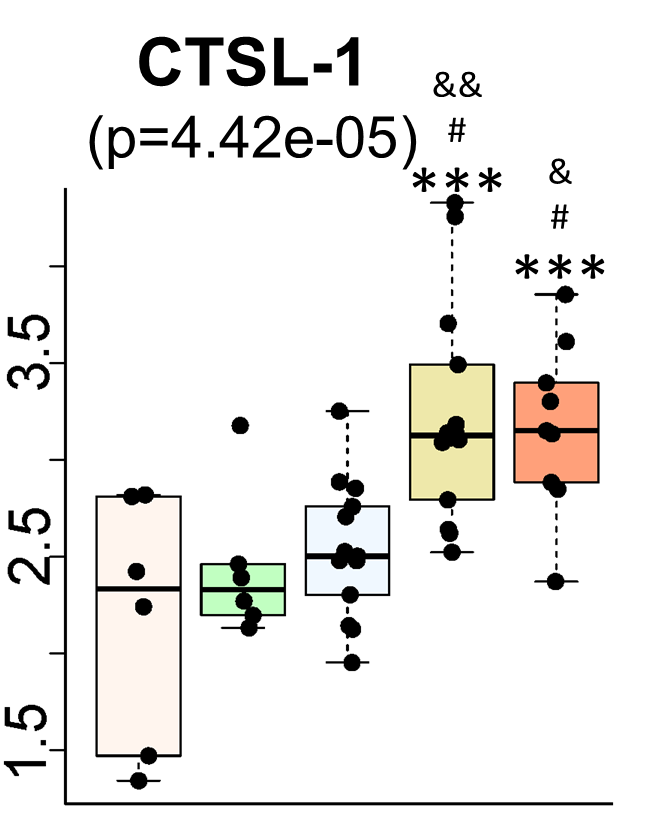

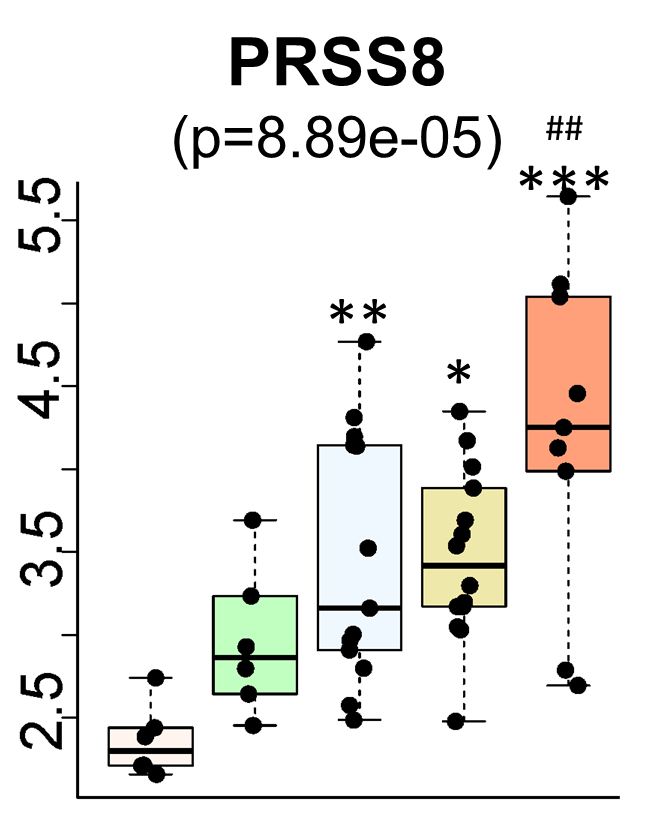

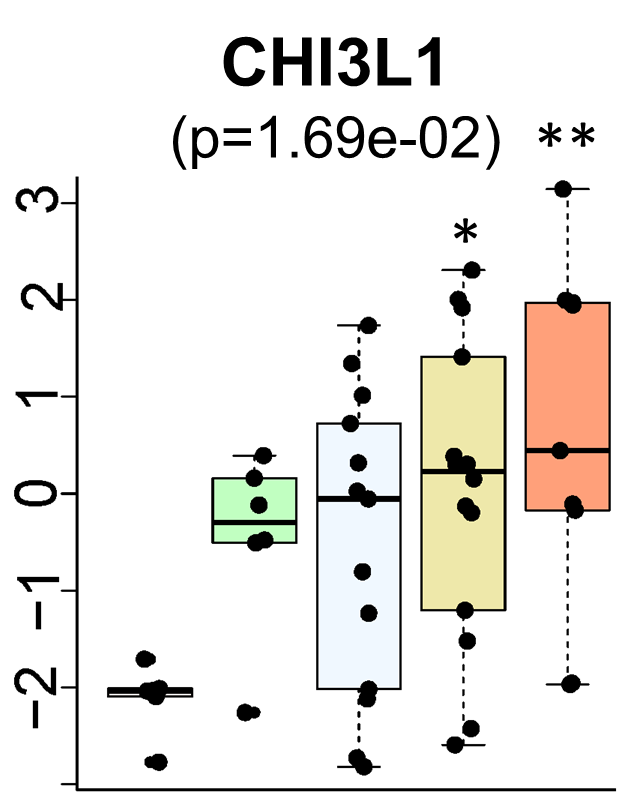

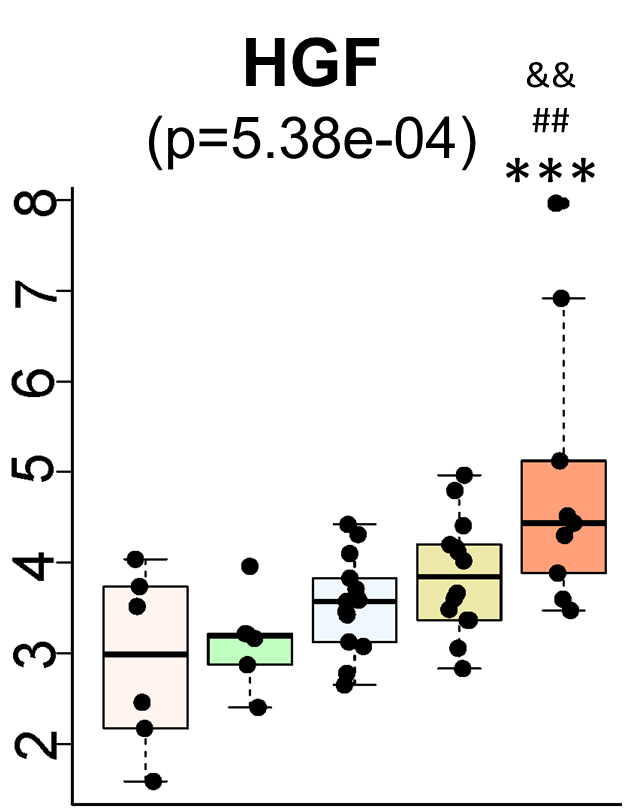

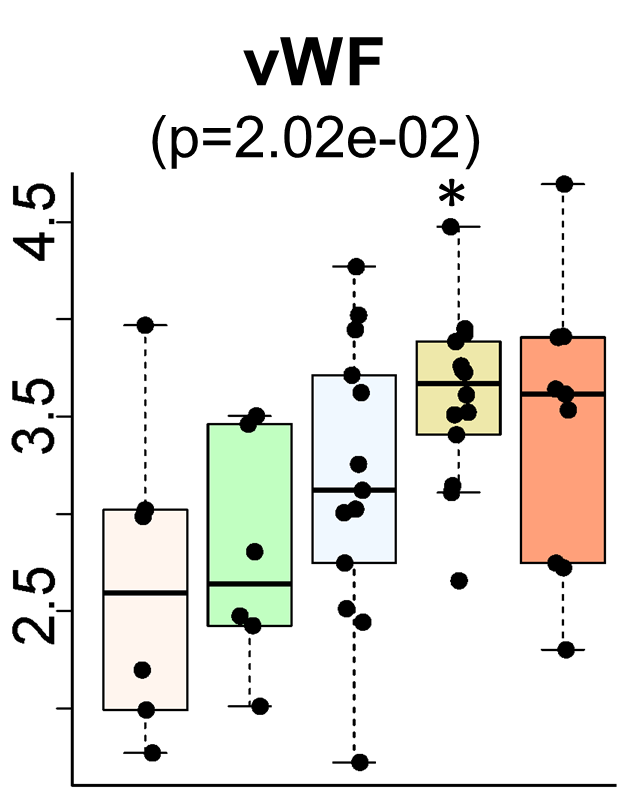

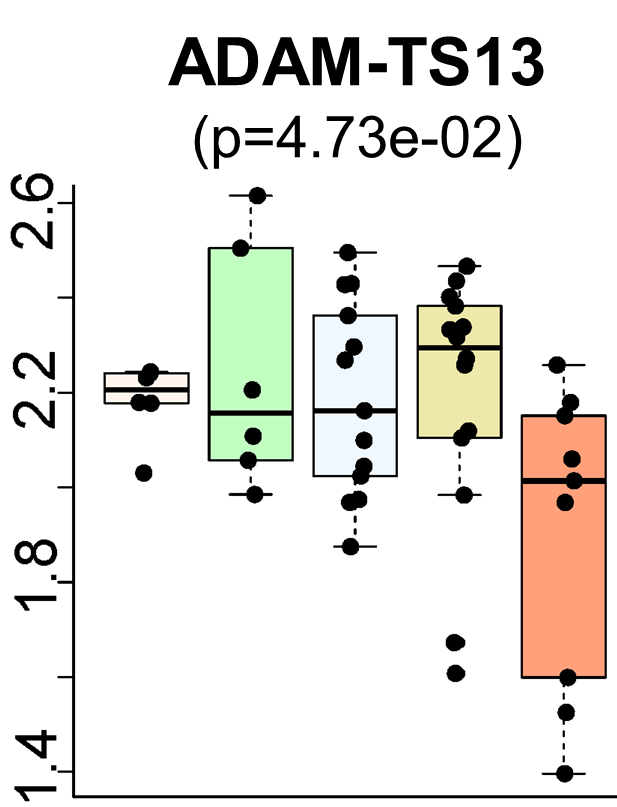

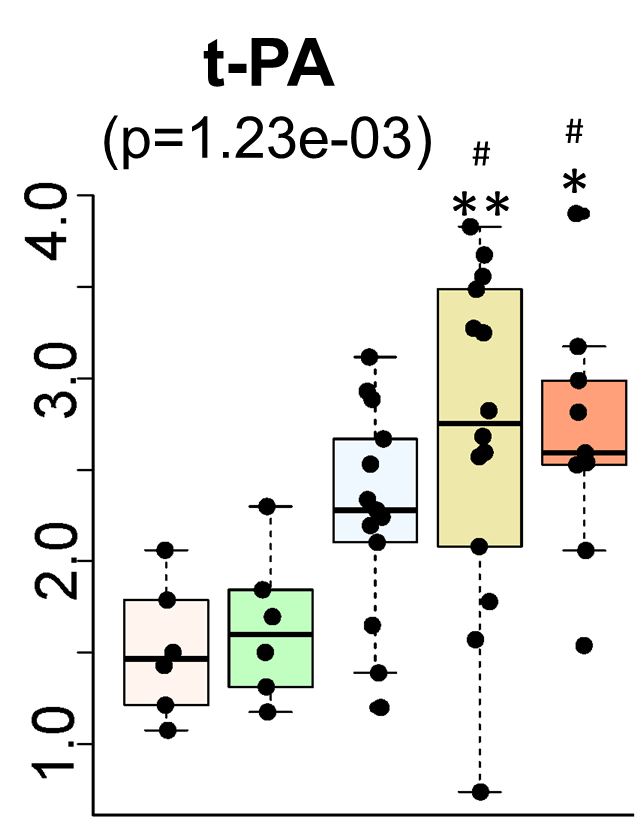

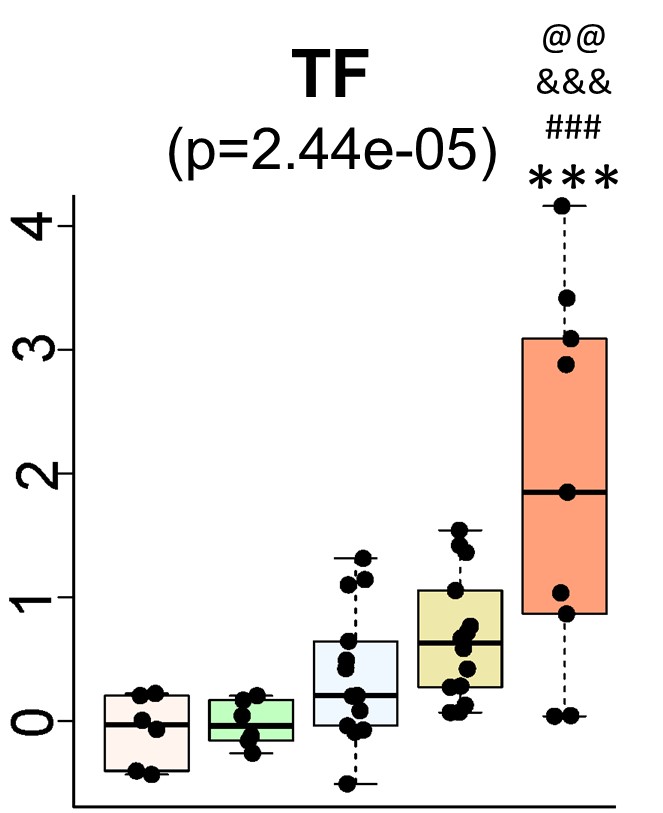

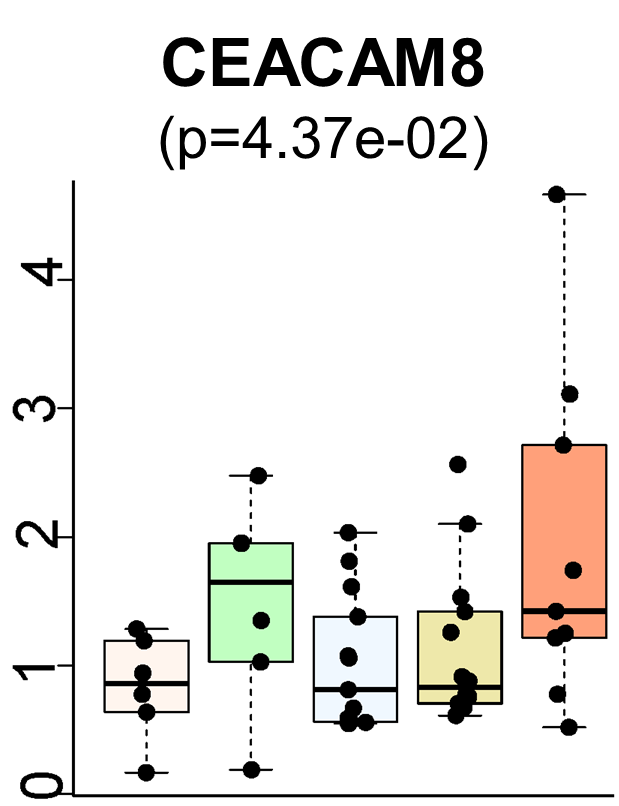

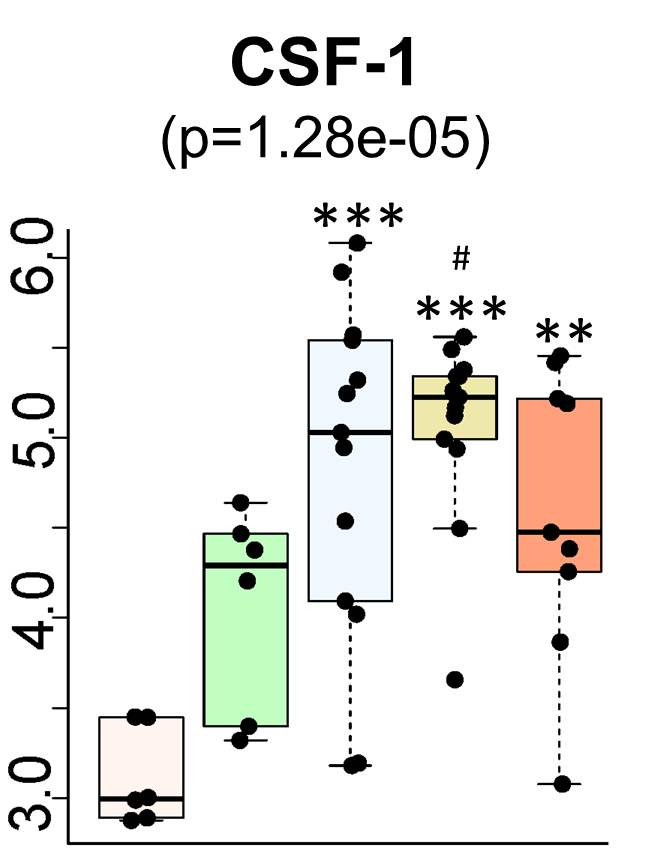

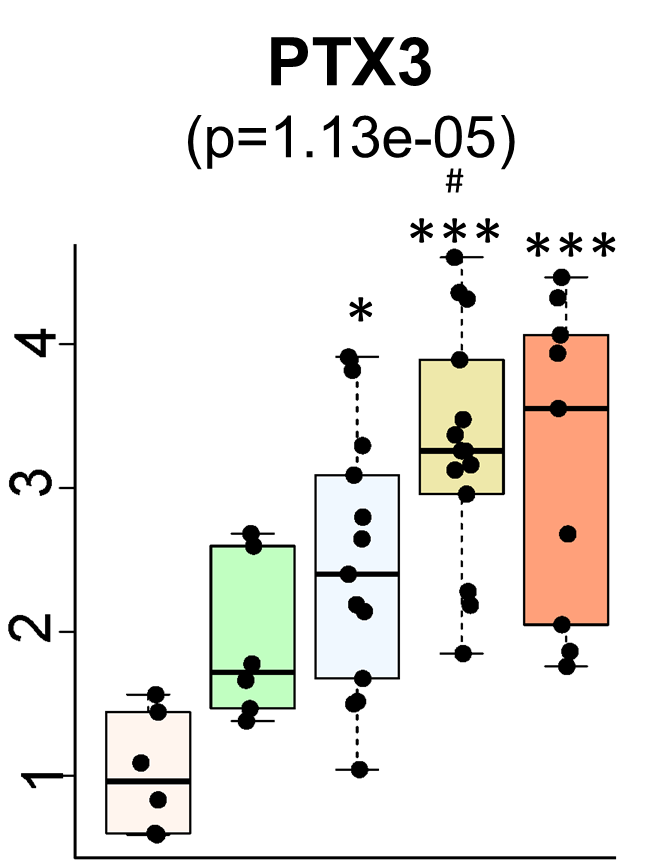

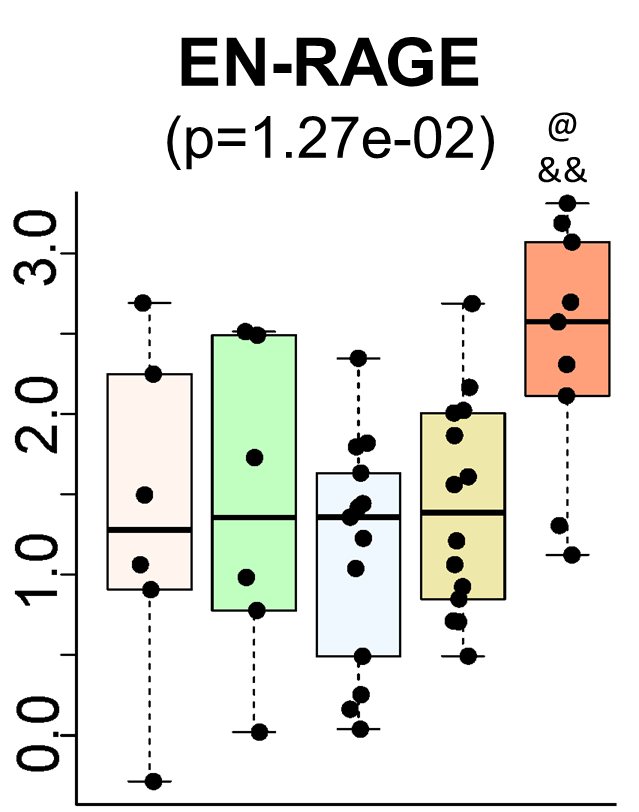

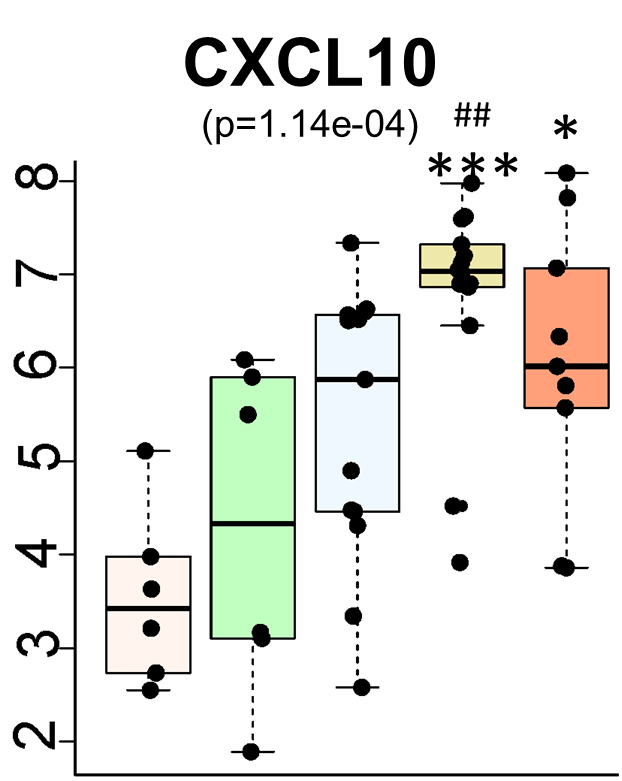

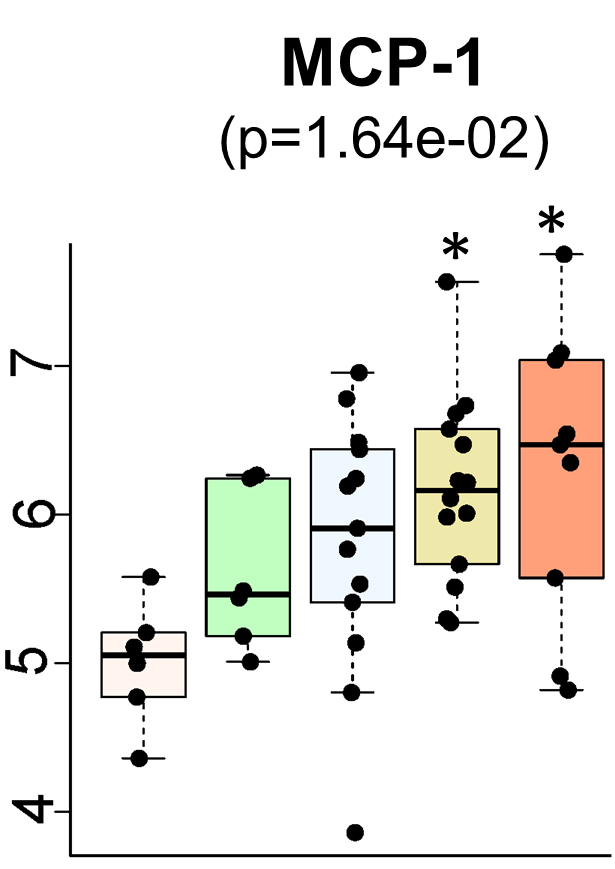

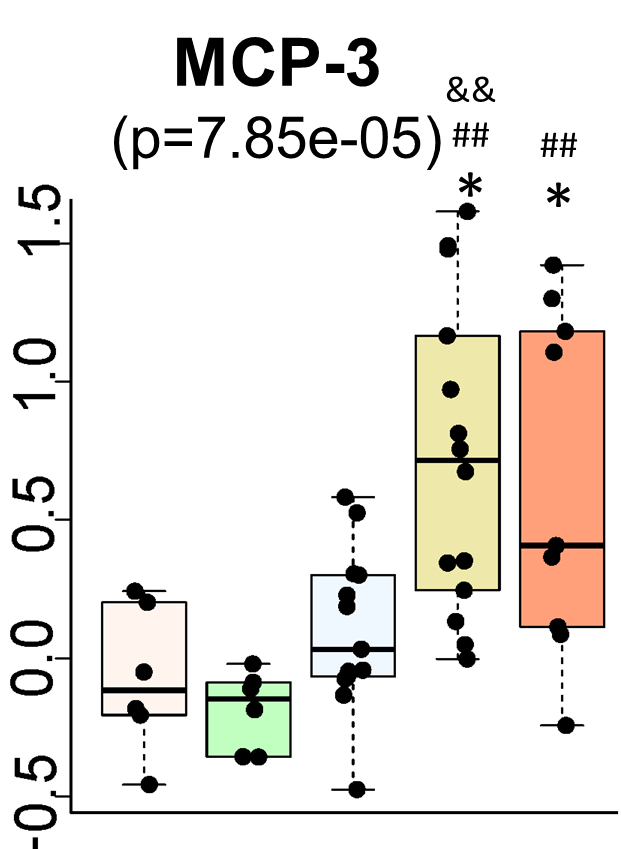

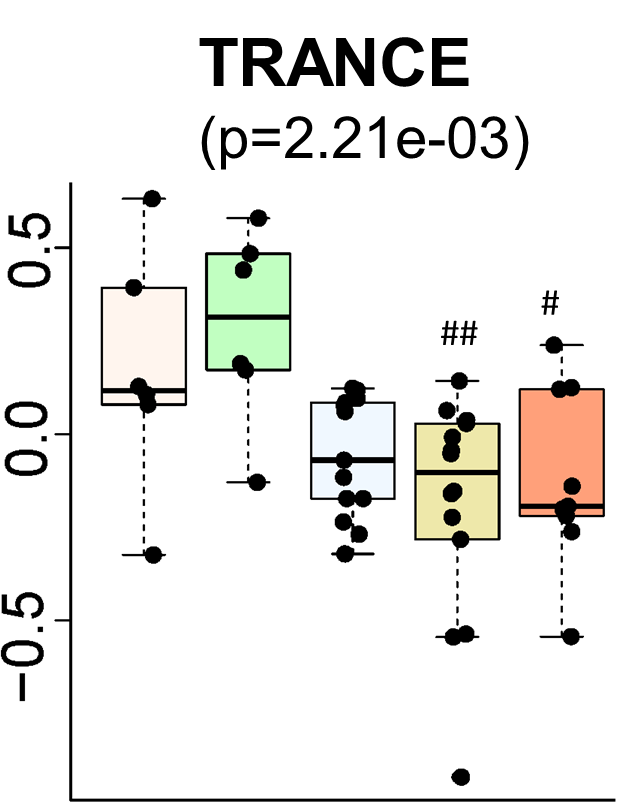

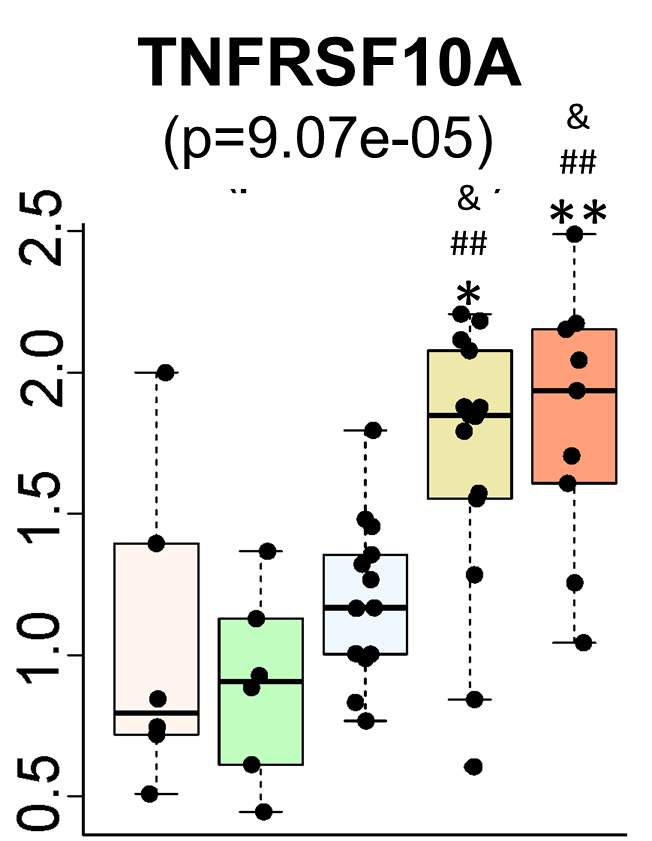

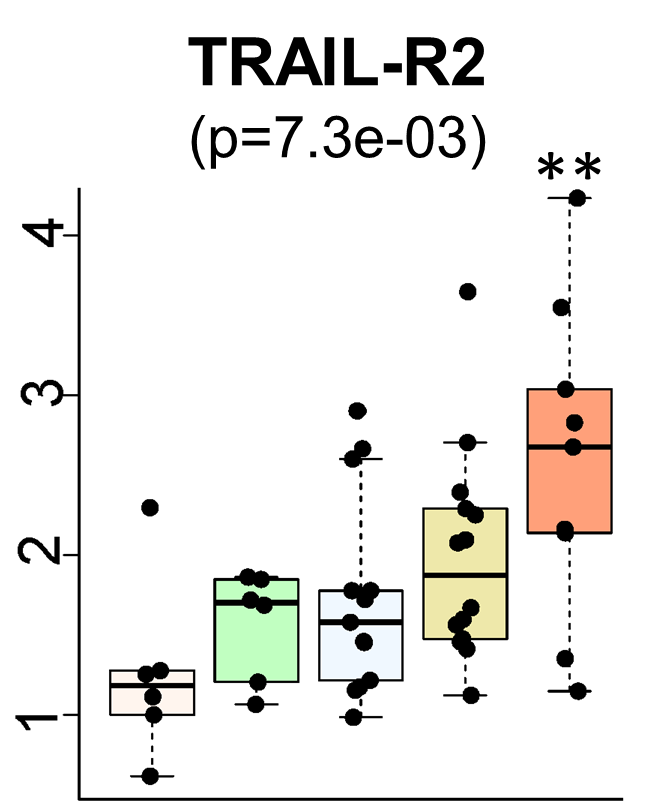

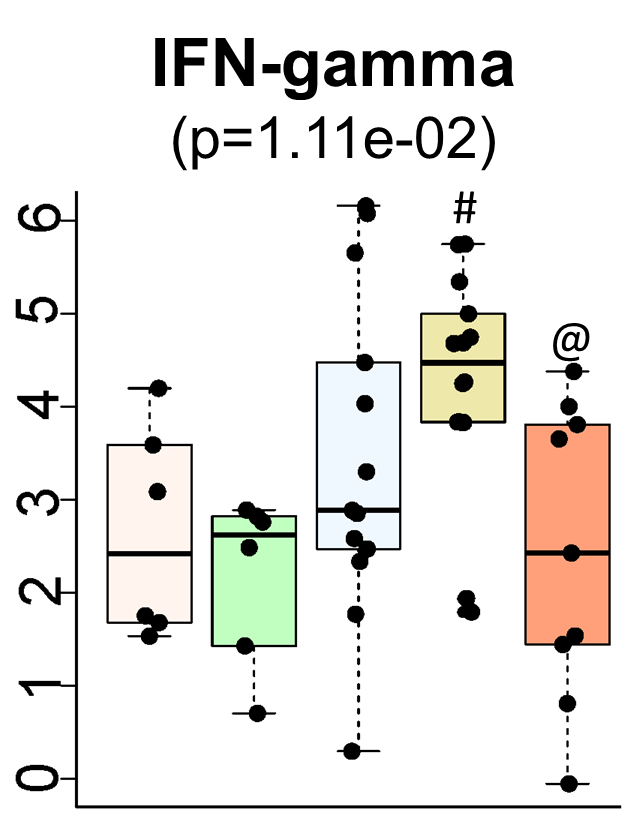

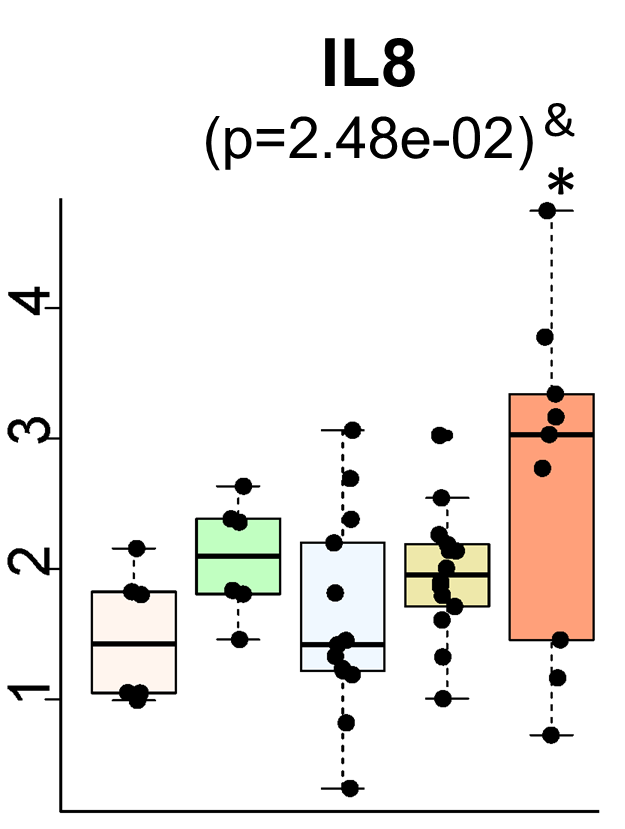

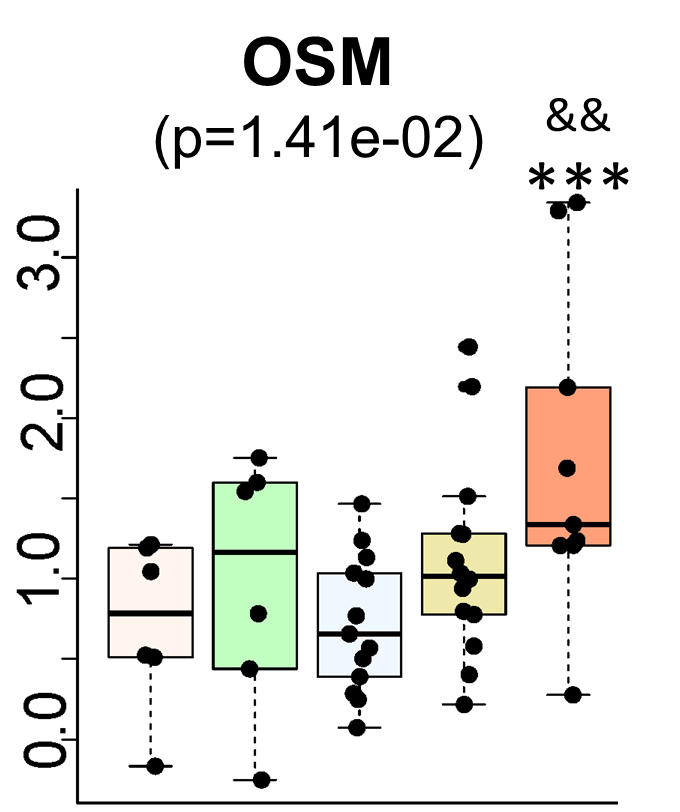

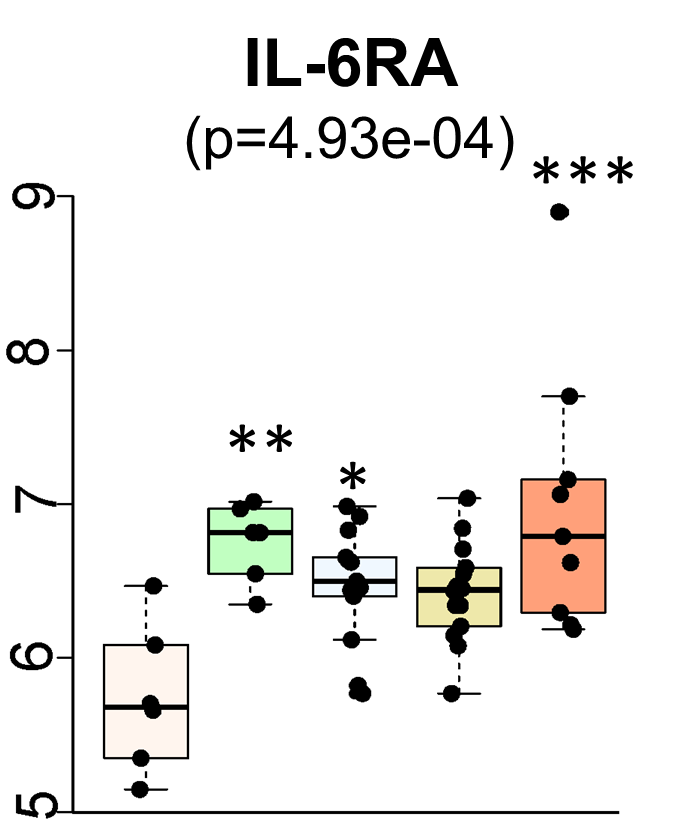

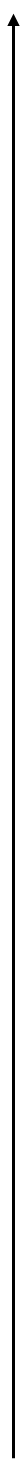

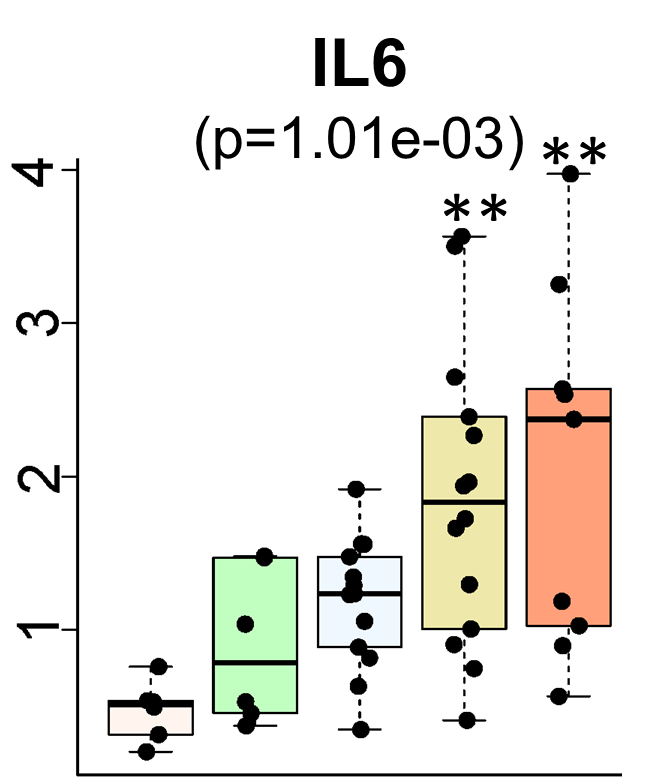


**NPX (log2)**

**Supplementary Figure 5: Box-whisker plots showing selected differentially expressed inflammatory and cardiovascular proteins in LEVs from COVID-19 patients.** Boxes span from quartile 1 and 3 with median showing in the middle and whiskers extend to 1.5 times the IQR from the box.* p<0.05, **p<0.01, ***p<0.001 vs. UI-Control, # p<0.05, ##p<0.01 ### p<0.001 vs. Asymptomatic, & p<0.05, && p<0.01, &&& p<0.001 vs. Moderate-No O2, @ p<0.05,@@ p<0.01 vs. Moderate-On O2.


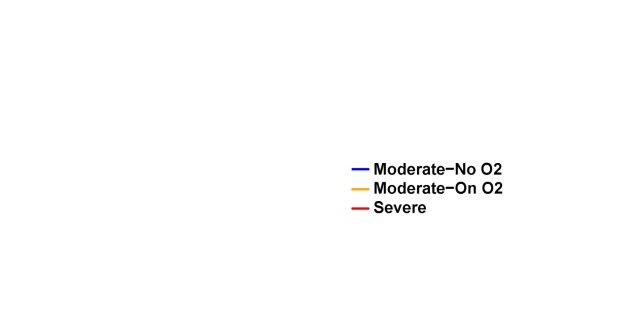

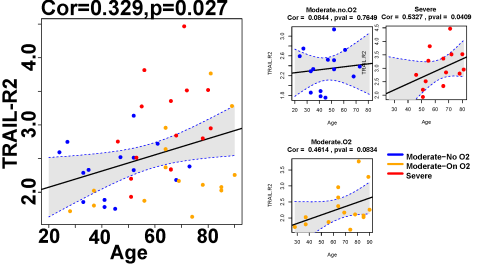

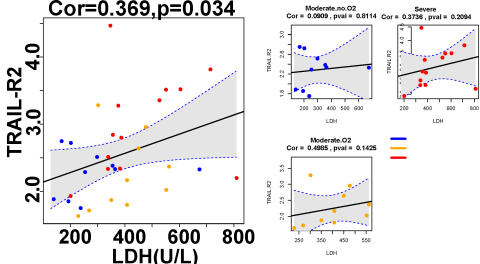

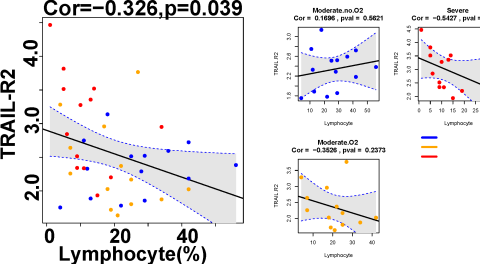

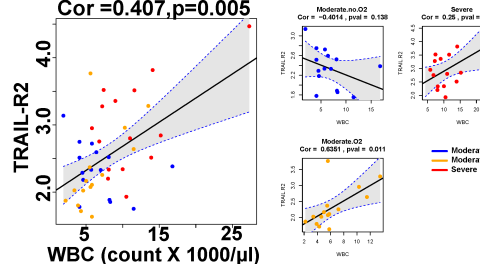

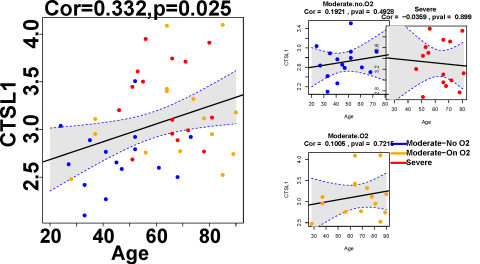

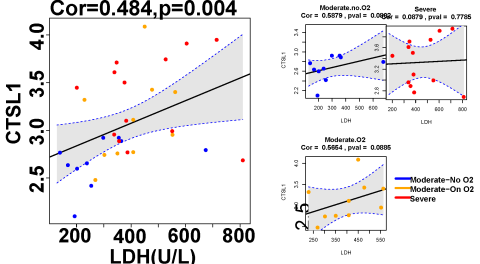

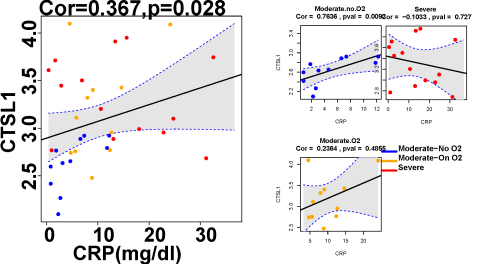

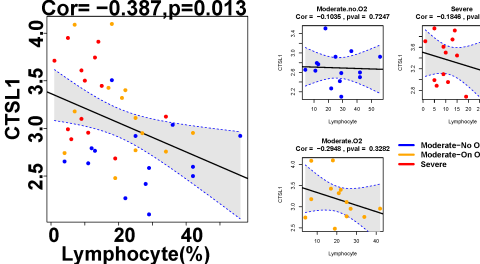

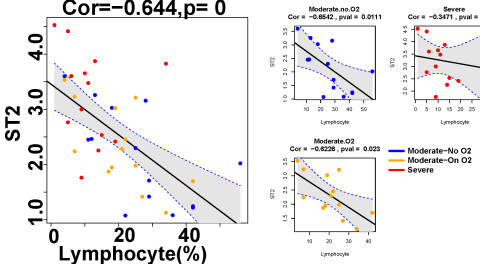

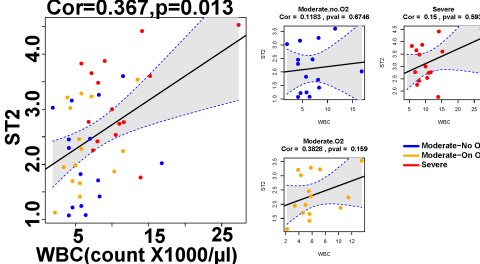

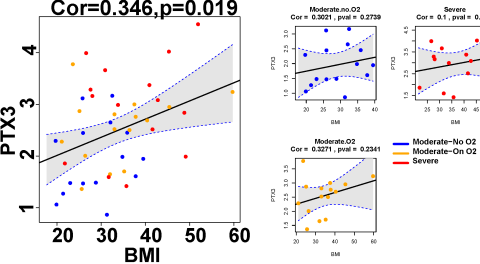

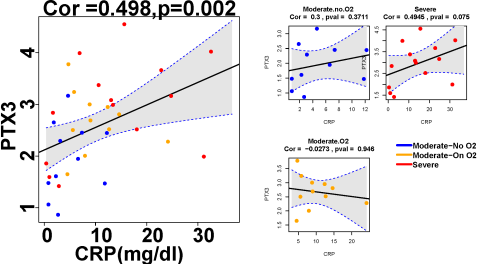

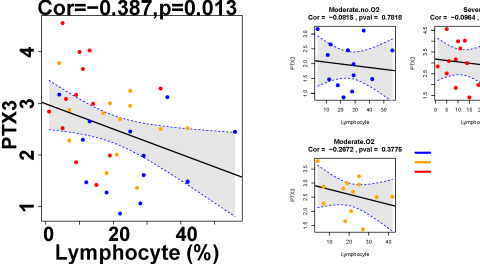

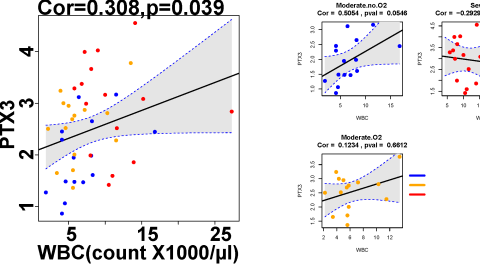

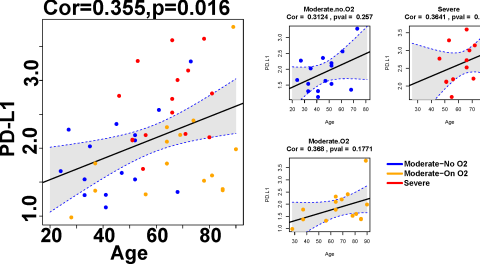

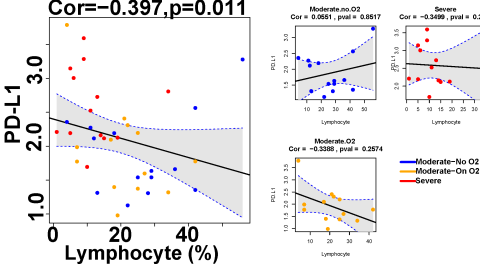

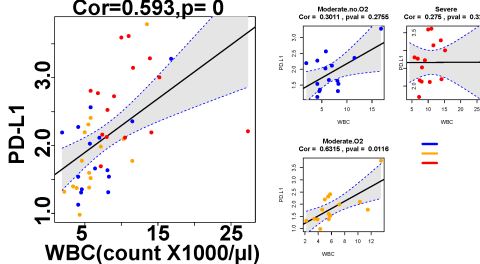

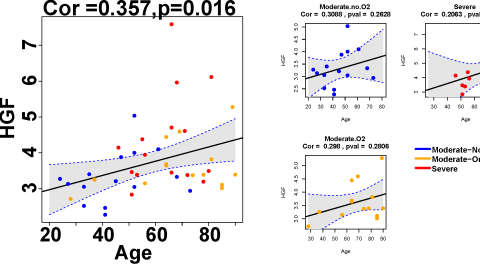

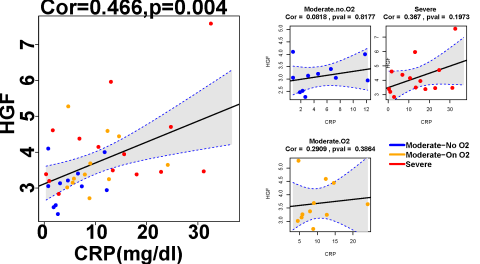

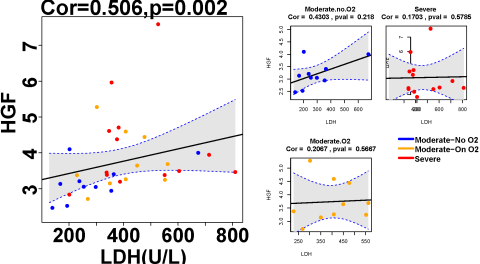

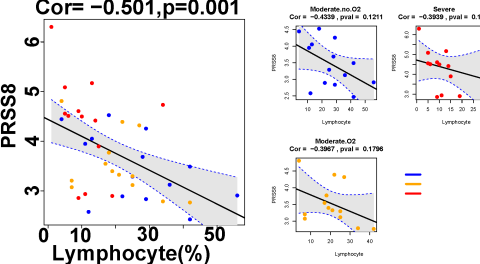

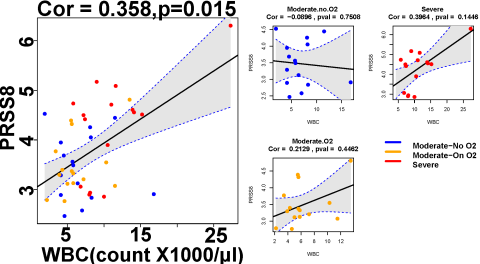

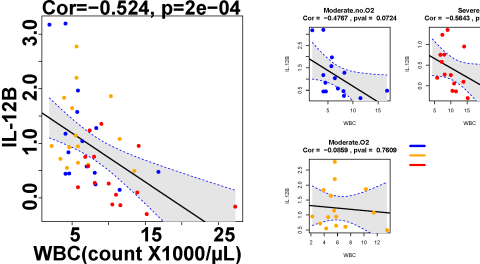

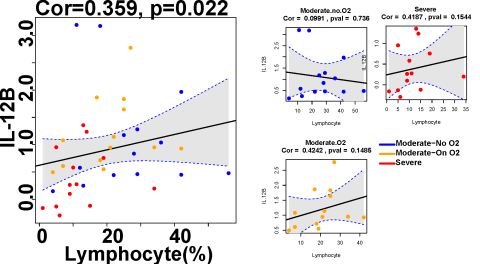

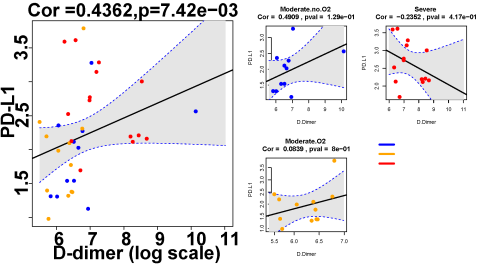

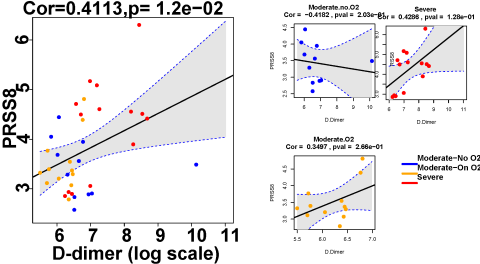

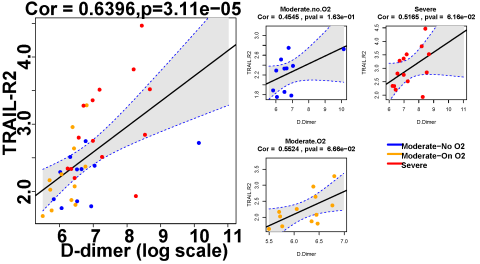

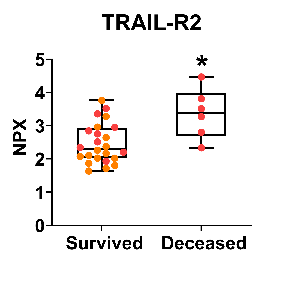

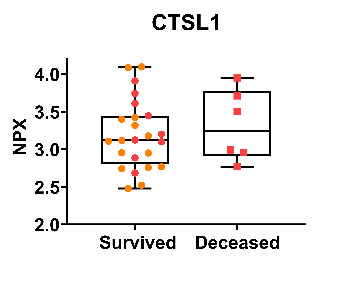

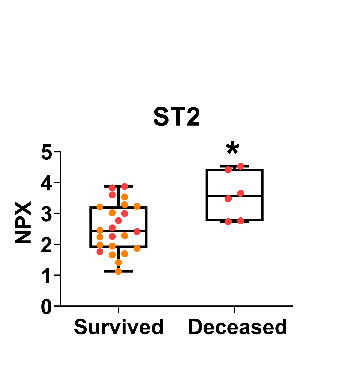

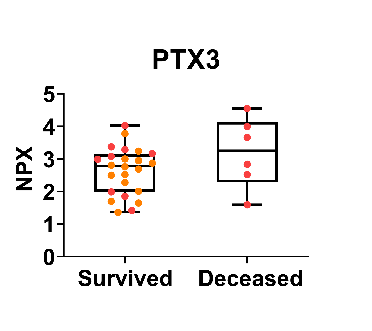

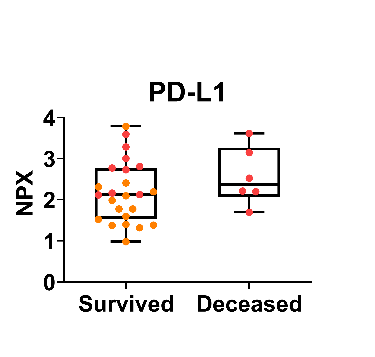


**A)**

**B)**

**C)**

**D)**

**E)**

**F)**

**G)**

**H)**

**I)**

**J)**

**K)**

**L)**

**M)**

**N)**

**O)**

**P)**

**Supplementary Figure 6**: **A-H)** Individual spearman correlation plots of selected molecules on SEVs that showed stronger correlation with disease severity when compared with other established parameters of COVID-19 outcomes. **I-P)** Comparison of EV linked levels of selected molecules in patients on oxygen support who survived (n=24) with patients on oxygen support who died (n=6) *p<0.05, vs Survived.

**A)**

**D)**

**E)**

**H)**

**I)**

**B)**

**C)**

**G)**

**F)**

**Supplementary Figure 7**: **Spearman’s correlation analysis of significantly altered LEV-linked proteins in COVID-19 symptomatic patients with age , BMI and clinical parameters** **A)** Correlation matrix of LEV-linked selected proteins ( NPX, Log2 ) with age, BMI, length of hospitalization (LH), disease severity (DS) based on WHO Clinical Progression Scale score, D-dimer (ng/ml), lactate dehydrogenase (LDH, units/L) , C-reactive protein (CRP,mg/dl) and lymphocytes (%) and WBC count (count x1000/µl) in symptomatic COVD-19 patients ( n= 36). The right inclined blue colored ellipses represent positive correlation and left inclined red colored ellipses represent negative correlation. Slimmer ellipse represents stronger correlation while circular shape indicating weaker correlation. (* p<0.05, ** p<0.01, *** p<0.001). **B-I)** Individual spearman correlation plots of selected molecules that showed stronger correlation with disease severity length of hospitalization or other parameters of disease outcomes.

**B)**

**C)**

**A)**

**Supplementary Figure 8: A)** Schematic representation of the EV-capturing experiment. Biotinylated antibodies against IL-18R1 and IL-6RA or IgG antibody were used to capture EVs. **B)** Flow cytometry of captured EVs stained with S/P Green dye. The bottom panels show the percentage of S/P Green dye stained EVs. **C)** Western blot showing the presence of EV markers in the captured population.

**Supplementary Table 1: List of antibodies and reagents used.**

| **Antibodies** | **Manufacturer** | **Catalogue #** | **Dilution** |
| --- | --- | --- | --- |
| **Primary** |  |  |  |
| Alix | CST | 2171T | 1:500 |
| Flotillin-1 | CST | 18634T | 1:500 |
| GM130 | CST | 12480T | 1:500 |
| CD9 | CST | 13174T/ 13174S | 1:500 |
| CD81 | Novus | NB100-65805SS | 1:500 |
| TSG101 | Novus | NBP2-77452SS | 1:500 |
| CD63 | Novus | NB100-77913SS | 1:500 |
| ApoE | Santa Cruz Biotechnology | SC-390925 | 1:500 |
| Integrin beta 1 | Abcam | ab52971 | 1:500 |
| **Secondary** |  |  |  |
| Goat Anti-Mouse IgG Antibody, (H+L) HRP conjugate | Millipore sigma | AP308P | 1:2500 |
| Goat Anti-Rabbit IgG Antibody, (H+L) HRP conjugate | Millipore sigma | AP307P | 1:2500 |
| **Biotinylated** |  |  |  |
| Biotin anti-human CD218a (IL-18Rα) Antibody | BioLegend | 313806 |  |
| Biotin anti-human CD126 (IL-6Rα) Antibody | BioLegend | 352808 |  |
| Biotin Mouse IgG1, Antibody | BioLegend | 400103 |  |
| Reagents & Kits |  |  |  |
| Blotting Grade blocker | Bio-Rad | 170-6404 |  |
| Resolving Gel Buffer (1.5M Tris HCl, pH 8.8) | Bio-Rad | 161-0798 |  |
| 0.5M Tris-HCl buffer | Bio-Rad | 161-0799 |  |
| 30% Acrylamide/Bis Solution 37.5:1 | Bio-Rad | 161-0158 |  |
| SDS | Fisher Scientific | BP166-500 |  |
| APS | Fisher Scientific | BP179-25 |  |
| TEMED | Sigma | T9281 |  |
| Tween 20 | Fisher Scientific | BP337-100 |  |
| Immobilon membrane | Millipore Sigma | IPVH00010 |  |
| 10X Tris/Glycine/SDS Buffer | Bio-Rad | 1610732 |  |
| 10X Tris/Glycine Buffer | Bio-Rad | 1610734 |  |
| Restore Western blot Stripping buffer | Thermo-Scientific | 21059 |  |
| Classic Autoradiography Film | MidSci | X199X |  |
| Methanol | Fisher Scientific | A452-1 |  |
| Piercing ECL WB substrate | Thermo Scientific | 32106 |  |
| RIPA Lysis Buffer System | Santa Cruz Biotechnology | sc-24948A |  |
| Tris Base | Fisher Scientific | BP152-1 |  |
| Sodium chloride | Fisher Scientific | BP358-212 |  |
| Sodium deoxycholate | Sigma | D6750 |  |
| EDTA | Fisher Scientific | BP120-500 |  |
| Exo-Flow™ 2.0 Basic Kit without antibody (Streptavidin beads + reagents) - for Serum or Plasma | System Biosciences | EXOFLOW2-BASICA-SP |  |
| Human S100A12 ELISA Kit | Abcam | ab49561 |  |
| Human Tissue Factor ELISA Kit | Abcam | ab108903 |  |
| Human Tissue Factor (TF) Chromogenic AssaySense Activity Assay Kit | Assay Pro | CT1002b |  |
| Vivaspin 300kDa filters | Sartorius | VS0151 |  |
| PKH67 Green Fluorescent Cell Linker Midi Kit for General Cell Membrane Labeling | Millipore Sigma | MIDI67 |  |
| ExoGlowTM-Membrane EV Labeling Kit | System Biosciences | EXOGM600A-1 |  |
| PBS | Corning | 21-040-CV |  |
| Carbon Film 300 Mesh, Copper Grid | Electron Microscopy Sciences | CF300-CU |  |
| Uranyl acetate | Electron Microscopy Sciences | 22400-3 |  |
| SEC Column | IZON qEV/35nm | SP5 |  |
| Pierce BCA protein Assay | Thermo scientific | 23225 |  |
| Alexa Fluor 594 Phallodin | Thermo-Scientific | A12381 |  |
| Prolong Gold Antifade Mountant with DAPI | Thermo-Scientific | P36935 |  |
| Amicon Ultra-4 centrifugal filter unit | Millipore Sigma | UFC801024 |  |
| Formaldehyde | Thermo-Scientific | 28908 |  |
| Triton X | Sigma | X100 |  |
| BSA | Sigma | A4503 |  |
| FITC Annexin V/Dead cell apoptosis Kit | Thermo-Scientific | V13242 |  |
| Cell meter Caspase 3/7 Assay apoptosis assay kit | AAT-Bio | 22796 |  |
| 96-well plate | Corning | 3603 |  |
| 24-well plate | Thermo Scientific | 142475 |  |
| 96-well PCR plate | Thermo Scientific | 4306737 |  |
| Endothelial Cell Medium | ScienCell | 1001 |  |
| Human Pulmonary Microvascular Endothelial Cells | ScienCell | 3000 |  |

**Supplementary Table 2: Total number of EVs corresponding to 3µg of protein used for the apoptosis experiment.**

| Groups | LEVs number for 3ug used for Apoptosis experiments×10^6^ | SEVs number for 3ug used for Apoptosis experiments ×10^6^ |
| --- | --- | --- |
| UI-Control-1 | 16.4 | 35.4 |
| UI-Control-2 | 30.2 | 37.2 |
| UI-Control-3 | 28.7 | 32.1 |
| UI-Control-4 | 139.8 | 167.1 |
| UI-Control-5 | 50.1 | 65.4 |
| UI-Control-6 | 50.7 | 98.1 |
| UI-Control-7 | 83.6 | 90.8 |
| UI-Control-8 | 129.8 | 116.0 |
| UI-Control-9 | 95.4 | 83.9 |
| UI-Control-10 | 50.5 | 28.7 |
| UI-Control-11 | 132.1 | 133.1 |
| UI-Control-12 | 26.8 | 59.3 |
| UI-Control-13 | 40.1 | 39.7 |
| UI-Control-14 | 50.5 | 60.6 |
| UI-Control-15 | 91.6 | 93.0 |
| Healthy Control-1 | 123.4 | 37.7 |
| Healthy Control-2 | 28.5 | 99.7 |
| Healthy Control-3 | 100.2 | 135.4 |
| Healthy Control-4 | 139.3 | 84.1 |
| Healthy Control-5 | 140.0 | 57.7 |
|  |  |  |
| Asymptomatic-1 | 104.4 | 70.4 |
| Asymptomatic-2 | 116.1 | 54.6 |
| Asymptomatic-3 | 209.1 | 150.6 |
| Asymptomatic-4 | 13.5 | 30.6 |
| Asymptomatic-5 | 175.3 | 94.0 |
| Asymptomatic-6 | 153.4 | 46.8 |
| Asymptomatic-7 | 18.6 | 37.4 |
| Asymptomatic-8 | 15.7 | 54.2 |
| Asymptomatic-9 | 74.3 | 64.6 |
|  |  |  |
| Moderate-No O2-1 | 294.2 | 127.7 |
| Moderate-No O2-2 | 159.0 | 80.3 |
| Moderate-No O2-3 | 67.8 | 144.8 |
| Moderate-No O2-4 | 272.9 | 54.7 |
| Moderate-No O2-5 | 189.8 | 95.6 |
| Moderate-No O2-6 | 272.7 | 93.1 |
| Moderate-No O2-7 | 190.6 | 124.1 |
| Moderate-No O2-8 | 148.0 | 78.1 |
| Moderate-No O2-9 | 137.9 | 83.1 |
| Moderate-No O2-10 | 233.2 | 91.3 |
| Moderate-No O2-11 | 55.2 | 52.1 |
| Moderate-No O2-12 | 66.2 | 219.0 |
| Moderate-No O2-13 | 86.1 | 44.5 |
| Moderate-No O2-14 | 178.7 | 136.2 |
| Moderate-No O2-15 | 134.0 | 283.0 |
|  |  |  |
| Moderate-On O2 -1 | 121.8 | 51.6 |
| Moderate-On O2 -2 | 23.9 | 31.5 |
| Moderate-On O2 -3 | 42.4 | 46.4 |
| Moderate-On O2 -4 | 242.6 | 88.2 |
| Moderate-On O2 -5 | 32.6 | 35.1 |
| Moderate-On O2 -6 | 148.8 | 49.9 |
| Moderate-On O2 -7 | 84.6 | 54.6 |
| Moderate-On O2 -8 | 50.4 | 19.3 |
| Moderate-On O2 -9 | 126.8 | 86.1 |
| Moderate-On O2 -10 | 71.2 | 62.3 |
| Moderate-On O2 -11 | 109.2 | 92.8 |
| Moderate-On O2 -12 | 23.3 | 46.0 |
| Moderate-On O2 -13 | 141.8 | 69.9 |
| Moderate-On O2 -14 | 104.0 | 47.8 |
| Moderate-On O2 -15 | 222.6 | 175.4 |
|  |  |  |
| Severe-1 | 185.3 | 114.7 |
| Severe-2 | 248.7 | 72.3 |
| Severe-3 | 166.0 | 102.1 |
| Severe-4 | 158.8 | 55.4 |
| Severe-5 | 132.6 | 79.8 |
| Severe-6 | 146.8 | 229.8 |
| Severe-7 | 92.1 | 35.8 |
| Severe-8 | 83.7 | 70.0 |
| Severe-9 | 176.5 | 88.0 |
| Severe-10 | 87.6 | 197.7 |
| Severe-11 | 84.2 | 142.5 |
| Severe-12 | 234.7 | 295.3 |
| Severe-13 | 94.1 | 319.2 |
| Severe-14 | 47.6 | 144.7 |
| Severe-15 | 127.4 | 132.0 |
